# Unraveling the genomic mosaic of a ubiquitous genus of marine cyanobacteria

**DOI:** 10.1186/gb-2008-9-5-r90

**Published:** 2008-05-28

**Authors:** Alexis Dufresne, Martin Ostrowski, David J Scanlan, Laurence Garczarek, Sophie Mazard, Brian P Palenik, Ian T Paulsen, Nicole Tandeau de Marsac, Patrick Wincker, Carole Dossat, Steve Ferriera, Justin Johnson, Anton F Post, Wolfgang R Hess, Frédéric Partensky

**Affiliations:** 1 Université Paris 6 and CNRS, UMR 7144, Station Biologique, 29682 Roscoff, France; 2 Université Rennes 1, UMR 6553 EcoBio, IFR90/FR2116, CAREN, 35042 Rennes, France; 3 Department of Biological Sciences, University of Warwick, Coventry CV4 7AL, UK; 4 Scripps Institution of Oceanography, UCSD, San Diego, CA 92093, USA; 5 Department of Chemistry and Biomolecular Sciences, Macquarie University, Sydney, NSW, Australia 2109; 6 Institut Pasteur, Dépt de Microbiologie, Unité des Cyanobactéries, URA 2172 CNRS, Paris, France; 7 Genoscope (CEA) and UMR 8030 CNRS-Genoscope-Université d'Evry, 91057 Evry, France; 8 J Craig Venter Institute, Rockville, MD 20850, USA; 9 The Interuniversity Institute for Marine Science, Hebrew University, Eilat 88103, Israel; 10 University of Freiburg, Faculty of Biology, D-79104 Freiburg, Germany

## Abstract

**Background:**

The picocyanobacterial genus *Synechococcus *occurs over wide oceanic expanses, having colonized most available niches in the photic zone. Large scale distribution patterns of the different *Synechococcus *clades (based on 16S rRNA gene markers) suggest the occurrence of two major lifestyles ('opportunists'/'specialists'), corresponding to two distinct broad habitats ('coastal'/'open ocean'). Yet, the genetic basis of niche partitioning is still poorly understood in this ecologically important group.

**Results:**

Here, we compare the genomes of 11 marine *Synechococcus *isolates, representing 10 distinct lineages. Phylogenies inferred from the core genome allowed us to refine the taxonomic relationships between clades by revealing a clear dichotomy within the main subcluster, reminiscent of the two aforementioned lifestyles. Genome size is strongly correlated with the cumulative lengths of hypervariable regions (or 'islands'). One of these, encompassing most genes encoding the light-harvesting phycobilisome rod complexes, is involved in adaptation to changes in light quality and has clearly been transferred between members of different *Synechococcus *lineages. Furthermore, we observed that two strains (RS9917 and WH5701) that have similar pigmentation and physiology have an unusually high number of genes in common, given their phylogenetic distance.

**Conclusion:**

We propose that while members of a given marine *Synechococcus *lineage may have the same broad geographical distribution, local niche occupancy is facilitated by lateral gene transfers, a process in which genomic islands play a key role as a repository for transferred genes. Our work also highlights the need for developing picocyanobacterial systematics based on genome-derived parameters combined with ecological and physiological data.

## Abstract 2

Local niche occupancy of marine *Synechococcus* lineages is facilitated by lateral gene transfers. Genomic islands act as repositories for these transferred genes.

## Background

Unicellular picocyanobacteria of the genera *Synechococcus *and *Prochlorococcus *contribute significantly to global oceanic chlorophyll biomass and primary production and play an important role in biogeochemical cycles [1-3]. Despite their close phylogenetic relatedness, these two groups differ markedly in their light-harvesting apparatus and nutrient physiology and, thus, ecological performance [4]. *Synechococcus *is ubiquitous, since cells of this genus are found in estuarine, coastal or offshore waters over a large range of latitudes [5,6], whereas *Prochlorococcus *is confined to warm (45°N-40°S) and mostly nutrient-poor oceanic areas [7-9]. Genetically distinct clades displaying different vertical depth distributions occur in the latter genus, explaining its wider vertical distribution in oceanic waters relative to *Synechococcus *[10]. These high light- (HL) and low light- (LL) adapted clades have been further subdivided into at least six ecotypes exhibiting distinct light and/or temperature optima as well as distributions in the field [11]. In *Synechococcus*, at least 10 [12], and as many as 16 [13-15], clades have been defined based on different phylogenetic markers and physiological characteristics [16]. For several of these clades, distinct broad spatial and seasonal distribution patterns have been described, mainly over horizontal scales [17-19]. Some clades are confined to high latitude, temperate waters (for example, clades I and IV), while others preferentially thrive at lower latitudes in warm, permanently stratified oceanic waters (for example, clades II and III [19-21]).

Examination of the relationships between ecology, gene content and genome structure in the *Prochlorococcus *genus has revealed evidence for drastic genome reduction in several *Prochlorococcus *clades [22,23], a process clearly started prior to the differentiation of HL and LL clades [24]. This sequential loss of genes, including some involved in nutrient uptake or photosynthesis, appears to have affected HL and LL clades differently, since HL isolates share 95 clade-specific genes and LL isolates 48 [23]. Pair-wise comparison of two closely related *Prochlorococcus *isolates (MED4 and MIT9312) revealed that gene losses are partially compensated by gains from lateral gene transfer (LGT) events [25]. Many of these horizontally acquired genes were found to be located in highly variable genomic regions or 'islands'. More generally, it seems that much of the genomic diversity between *Prochlorococcus *isolates occurs in 'the leaves of the tree', that is, between the most closely related strains, and that gene islands are important in maintaining this diversity as reservoirs for laterally transferred genes [23].

Less is known about the extent and causes of genome diversity in marine *Synechococcus*. Strain WH8102 was also shown to possess genomic regions comparable to 'pathogenicity islands' and containing many glycosyltransferases [26]. A pair-wise comparison between this oligotrophic strain and a coastal isolate (CC9311) showed that LGT may have an important role in niche differentiation in this group, for example, by allowing acquisition of novel metal utilization capacity [27].

With the aim of further understanding the evolutionary processes driving genome divergence and niche adaptation in marine *Synechococcus*, we obtained sequences of nine additional genomes. By comparing them alongside three representative *Prochlorococcus *genomes, we calculated the relative sizes of the core and accessory genomes, estimated the importance and relative contribution of vertical inheritance and LGT for the core and accessory gene complements and examined the distributions of accessory genes with regard to genomic islands. In so doing, we identified a major influence of these islands in genome flexibility and found evidence that at least one of them plays a major role in colonization of new light niches. Moreover, by exploring the picocyanobacterial species concept, through study of the relationships between ribotype and genome diversity, we significantly advance our understanding of the phylogeny and evolution of this major group of marine photosynthetic prokaryotes.

## Results and discussion

### General features of the *Synechococcus *genomes

The 11 *Synechococcus *strains analyzed here include isolates from the Mediterranean Sea, the Red Sea, and the Pacific and Atlantic Oceans (Table 1). This set of strains covers nine of the ten clades defined by Fuller and co-workers [12] in marine sub-cluster 5.1, and also includes one sub-cluster 5.2 representative, the euryhaline, phycocyanin-rich strain WH5701. Though some of these genomes are incomplete, the estimated genome coverage is above 99.8% and, therefore, only a few genes are potentially missing, making global genome comparisons legitimate. Genomes range in size from 2.22 to approximately 2.86 Mbp and GC contents vary from 52.5% to 66.0%. This relatively small range of variation in genome characteristics is strikingly different from that observed in the *Prochlorococcus *genus, in which genome size varies between 1.64 and 2.68 Mbp, whilst GC content varies between 30.8% and 50.7% [23]. This observation suggests that, in sharp contrast to what has occurred in *Prochlorococcus *[22,24], no extensive genome streamlining, concomitant with a drop in GC content, has occurred during the evolution of *Synechococcus*.

**Table 1:** Summary of genome sequences used in this study

Strain	Sub-cluster	Clade	Location of isolation	Coordinates	Depth (m)	Genome size (Mbp)	Status	Scaffolds	Contigs	rRNA	Genes	%GC	References
*Synechococcus*													
CC9311	5.1	I	California current, Pacific (coastal)	32°00'N, 124°30'W	95	2.61	Complete: CP000435	1	1	2	2,944	52.5	[27]
CC9605	5.1	II	California current, Pacific (oligotrophic)	-	51	2.51	Complete: CP000110	1	1	2	2,645	59.2	This work
WH8102	5.1	III	Tropical Atlantic	22°30'N, 65°36'W		2.43	Complete: BX548020	1	1	2	2,583	59.4	[26]
CC9902	5.1	IV	California current, Pacific (oligotrophic)	-	5	2;23	Complete: CP000097	1	1	2	2,358	54.2	This work
BL107	5.1	IV	Blanes Bay, Mediterranean Sea	41°43'N, 3°33'E	1,800	2.28	WGS: AATZ00000000	1	6	2	2,553	54.2	This work
WH7803	5.1	V	Sargasso Sea	33°45'N, 67°30'W	25	2,37	Complete: CT971583	1	1	2	2,586	60.2	This work
WH7805	5.1	VI	Sargasso Sea	33°45'N, 67°30'W		2.62	WGS: AAOK00000000	1	13	2	2,934	57.5	This work
RS9917	5.1	VIII	Gulf of Aqaba, Red Sea	29°28'N, 34°55'E	10	2.58	WGS: AANP00000000	1	9	2	2,820	64.8	This work
RS9916	5.1	IX	Gulf of Aqaba, Red Sea	29°28'N, 34°55'E	10	2.66	WGS: AAUA00000000	1	4	2	3,009	59.8	This work
WH5701	5.2	-	Long Island Sound	-	-	2.86*	WGS: AANO00000000	2	9	2	3,129	66.0	This work
RCC307	5.3^†^	-	Mediterranean Sea	39°10'N, 6°17'E	15	2.22	Complete: CT978603	1	1	1	2,583	60.8	This work
													
*Prochlorococcus*													
MIT9313		LL	Northern Atlantic	37°05'N, 68°02'W	135	2.41	Complete: BX548175	1	1	2	2,330	50.7	[22]
SS120		LL	Sargasso Sea	28°59'N, 64°21'W	120	1.75	Complete: AE017126	1	1	1	1,930	36.4	[48]
MED4		HLI	Mediterranean Sea	43°12'N, 06°52'E	5	1.66	Complete: BX548174	1	1	1	1,763	30.8	[22]

*The WGS release includes 135 contigs; many of these are very small and are likely to be from a co-cultured contaminant. ^† ^This work; formerly subcluster 5.1, clade X [12].

### Core genome

**Figure 1:**
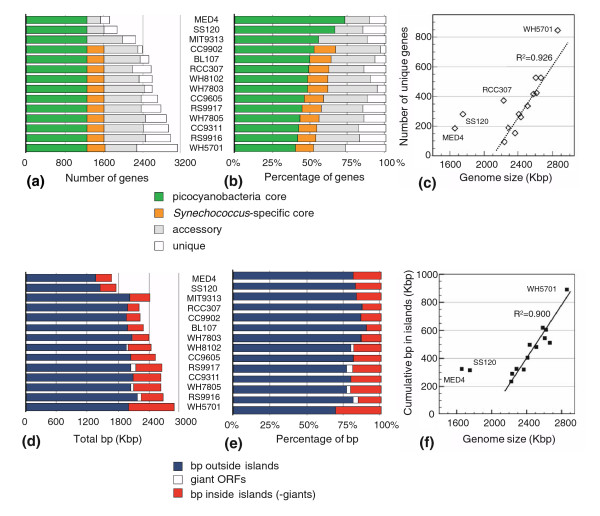
The core and accessory genomes of marine picocyanobacteria. **(a) **Number of genes distributed between the core and accessory components of each of the 11 *Synechococcus *and 3 *Prochlorococcus *genomes used in this study. The core genome common to all picocyanobacteria is indicated as green bars. The *Synechococcus*-specific core genome includes an additional set of genes shown as orange bars. The accessory genome is split between unique genes, indicated as white bars, and genes shared between 2-13 genomes, indicated as light grey bars. Note that when considering marine picocyanobacteria, genes shown in orange are part of the accessory genome. **(b) **Same as (a) but showing percentage of genes. **(c) **Number of unique genes relative to genome size. **(d) **Cumulative size of islands (red bars) and giant open reading frames (ORFs; white bars) relative to total genome size. **(e) **Same as (d) but showing percentage of base-pairs. **(f) **Cumulative length of islands versus size of *Synechococcus *genomes.

As a framework for comparative analyses and annotation, we constructed clusters of protein-coding genes for the 14 genomes analyzed in this study. From a set of 35,946 protein-coding genes, 7,826 distinct groups of homologous proteins were identified. The estimated core genome of marine *Synechococcus *is composed of 1,572 gene families (Figure 1a) which represent from as low as 52% of the total genome of WH5701 to as high as 67% in CC9902 (Figure 1b). Most families (93.4%) of the core genome contain only one gene from each strain, indicating a low level of paralogy. When adding three *Prochlorococcus *strains in the comparative analysis, the core genome is reduced to 1,228 gene families (Figure 1a). This number can be compared with the cyanobacterial core genome (that is, including both freshwater and marine cyanobacteria), which comprises 892 families of orthologs [28]. As expected, the streamlined *P. marinus *MED4 and SS120 genomes have the highest percentage of core genes (Figure 1b).

Only 70 gene families of the marine *Synechococcus *core genome are not present in any of the three *Prochlorococcus *genomes, including 23 linked to photosynthesis (Additional data file 1). Among these, there are nine gene families encoding allophycocyanin and phycocyanin components, which are shared with freshwater cyanobacteria [29]. Indeed, *Prochlorococcus *have lost all phycobilisome genes except those encoding phycoerythrin, with LL ecotypes having kept many of the latter genes and HL ecotypes only a few [30,31]. The RubisCo gene region includes three genes involved in low affinity carbon transport (*ndhD4*, *ndhF4 *and *chpX *homologs) that are missing in *Prochlorococcus*, confirming earlier results on a limited set of picocyanobacterial genomes [32]. Also notable in this *Synechococcus*-specific set are *ftrC *and *ftrV*, two genes encoding subunits of ferredoxin:thioredoxin reductase, an enzyme involved in a redox system between thioredoxin and ferredoxin [33]. All *Synechococcus *also have one gene coding for a thioredoxin and another for a [2Fe-2S] ferredoxin that have no orthologs in *Prochlorococcus *and it is tempting to speculate that their products might specifically be involved in the interaction with ferredoxin:thioredoxin reductase. This system could ensure the regulation by light of photosynthetic CO_2 _assimilation enzymes, a capacity that could have been lost (or evolved into a less iron-dependent form) in *Prochlorococcus*.

### Accessory genome and gene islands

The accessory genome of marine *Synechococcus *comprises a fairly constant number (748 ± 85) of genes shared by 2-10 genomes (Additional data file 2). Among the most notable genes are *isiA *and *isiB *(encoding the photosystem I-associated antenna protein CP43' and the soluble electron transport protein flavodoxin, respectively), which are systematically found associated in an iron-stress inducible operon in freshwater cyanobacteria but which in marine *Synechococcus *are found separated and present in only four strains (BL107, CC9311, CC9605 and CC9902). The absence of these genes in the oligotrophic strain WH8102 is particularly surprising, given their potential importance in the adaptation to low iron environments [34,35]. Interestingly, the four aforementioned *Synechococcus *strains also have a specific ferredoxin gene (among four to five gene copies in total) and it is possible, therefore, that this form is functionally interchangeable with flavodoxin, when cells are shifted from an iron-replete to an iron-limited environment [36].

**Figure 2:**
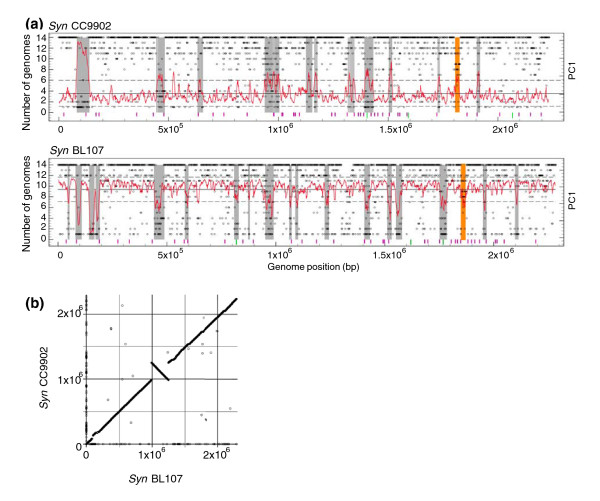
Genome plot of recently acquired genomic islands in *Synechococcus *spp. BL107 and CC9902 and whole genome alignment showing the positions of orthologous genes. **(a) **Genome plot with predicted islands highlighted in grey, except the phycobilisome gene cluster, which is highlighted in orange. The frequency with which a gene appears among the 14 genomes analyzed is represented by an open circle (that is, a core gene is present in 14 genomes). Deviation in tetranucleotide frequency is plotted in red as the first principal component in overlapping six gene intervals relative to the mean of the entire genome (black line) and standard deviation (broken black lines). The position of tRNA genes (purple bars) and mobility genes, such as those encoding phage integrases and transposases, are also indicated (green bars). **(b) **Whole genome alignment of *Synechococcus *BL107 and CC9902 showing the positions of orthologous genes.

**Figure 3:**
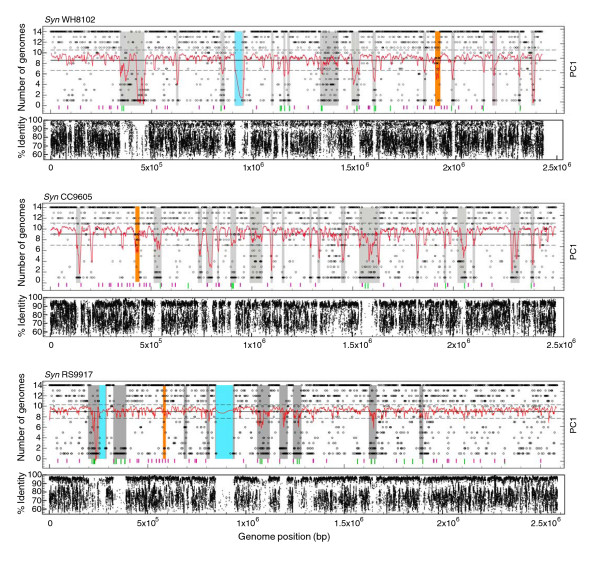
Genome plots of recently acquired islands in *Synechococcus *spp. WH8102, CC9605 and RS9917 and recruitment plots of environmental DNA fragments sampled during the GOS expedition [56]. Predicted islands are highlighted in grey, except the phycobilisome gene cluster which is highlighted in orange, and the giant open reading frames which are highlighted in blue. The frequency with which a gene appears among the 14 genomes analyzed is represented by an open circle (that is, a core gene is present in 14 genomes). Deviation in tetranucleotide frequency is plotted in red as the first principal component in overlapping six gene intervals relative to the mean of the entire genome (black line) and standard deviation (broken black lines). The position of tRNA genes (purple bars) and mobility genes, such as those encoding phage integrases and transposases, are also indicated (green bars). Note the good match (in most cases) between the location of islands (mainly predicted by deviation of tetranucleotide frequency) and a dramatic decrease of the frequency of hits from natural samples. This observation clearly demonstrates the strong variability of the gene content of islands.

The number of unique genes - that is, genes specific to one genome - is much more variable (91-845; Figure 1a). The latter number is strongly correlated with genome size (Figure 1c), except for the streamlined genomes of *P. marinus *MED4 and SS120 and the two most distantly related *Synechococcus *genomes, RCC307 and WH5701 (see phylogenetic analyses below), which all have an apparent excess of unique genes relative to their size. A large proportion (51-80%) of these unique genes are localized in 'islands' (Figure 1d), as predicted chiefly via deviation in tetranucleotide frequency. These islands (illustrated in Figures 2a and 3 and Additional data files 3 and 4) represent a very variable part of the total genome sequence (10.6-31.2%; Figure 1e). In addition, the average size of intergenic regions is higher here than in the rest of the genome (for example, >105 bp within islands and approximately 50 bp outside islands in WH7803). This, added to the high variability of island size, results in a strong correlation between the cumulative length of islands and the size of *Synechococcus *genomes (r^2 ^= 0.90; Figure 1f).

Island size and position are very variable among genomes (Additional data file 3), except for the closely related strains BL107 and CC9902 (Figure 2a), which show a high degree of co-linearity (Figure 2b). Even so, related islands can be identified in different genomes by the fact they are surrounded by homologous genes or gene regions (an example of such related islands is provided in Additional data file 4). Some islands are present in a large subset of strains and are likely ancient while others are present in only one or very few genomes, suggesting that they have been more recently acquired. We cannot exclude, however, that some of the islands present in few genomes could have been present in ancestral *Synechococcus *genomes but lost during subsequent speciation associated with colonization of new niches.

Gene composition of islands is also highly variable among *Synechococcus *genomes. A high percentage (37-79%) of island genes are shared by several genomes (though this is most often a small subset of the 11 genomes), suggesting that many genes acquired by LGT are maintained over time periods long enough to be disseminated within the host clade and eventually to more recently diverged *Synechococcus *lineages. The high variability of gene composition in these genomic regions is further demonstrated by comparing *Synechococcus *genomes with the Global Ocean Sampling (GOS) expedition database [37]. Environmental sequences from oceanic areas showed highest similarity to the WH8102 and CC9605 genomes whereas sequences from a hypersaline lagoon were most similar to RS9917. For all three genomes, there was generally a low recruitment of environmental sequences to island regions (Figure 3), giving us strong confidence in the reliability of our island predictions. This low recruitment raises questions about the origin of genes present in islands. Indeed, it may suggest that these genes are rare in the environment (that is, not belonging to any abundant groups of organisms) and, hence, that such islands are hypervariable. However, it is also possible that the source organisms may have been missed by the sampling strategy used to acquire the GOS data, either because they were too large (for example, bacteria retained on the 0.8 μm pre-filter) or too small (for example, phages passing through the 0.2 μm collecting filter). More metagenomic data, acquired using different sampling strategies, are clearly needed to resolve this important issue.

Altogether, our data suggest that, like for *Prochlorococcus *[23], genomic islands have a key role in the variability of *Synechococcus *genome sizes (and, therefore, their diversity), acting as a repository for novel genes. Those genes providing a sufficient selective advantage can be kept long term while others are more or less rapidly eliminated, depending on their effect on cell fitness. However, the underlying mechanism leading to preferential insertion of laterally transferred genes into these regions still needs to be elucidated.

### Function of island genes

Most island genes (60-78%) cannot be assigned to functional categories based on homology. Among island genes with known function (Additional data file 5), the predominant category comprises members of the glycosyltransferases and glycoside hydrolase gene families, potentially involved in outer membrane or cell wall biogenesis. As suggested previously [26,27], they may have a key role in grazer and phage avoidance. Other major categories include genes encoding enzymes involved in carbohydrate modification, ABC transport, mobility of DNA (for example, phage integrases and transposases) or transcriptional regulators (Additional data file 5). Also, putative genes of unusually large size (ranging from 5,016-84,534 bp), so-called 'giant open reading frames'; highlighted in blue in Figure 3 and Additional data files 3 and 5), frequently exhibit a significant deviation in tetranucleotide frequency and, according to recruitment plots against GOS data, appear to be very unevenly represented in the *Synechococcus *genomes (Figure 3). Only one of these giant proteins has been characterized so far in marine *Synechococcus*, the SwmB protein, which in WH8102 is required for a unique form of swimming motility [38].

**Figure 4:**
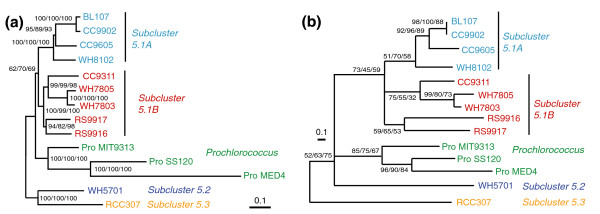
Phylogenetic relationships of marine *Synechococcus *and *Prochlorococcus*. **(a) **Unrooted distance tree based on concatenated alignments of 1,129 core proteins (307,756 amino acid positions) excluding families with paralogs. **(b) **16S rRNA gene phylogeny constructed with NJ. Numbers at nodes indicate bootstrap values for distance, parsimony and ML trees, respectively.

In a recent study, we described a region that gathers most genes encoding phycobilisome rod components (Figure 4 in [29]). Here, we show that in all *Synechococcus *genomes except the phycoerythrin II-lacking strains WH5701, RS9917 and WH7805, this region, ranging from 9-28.5 kb, depending on strain, displays a significant deviation in tetranucleotide frequency (region highlighted in orange in Figures 2 and 3 and Additional data file 5) and, therefore, it has the properties of an island. This finding is consistent with the fact that phylogenetic trees inferred from genes contained in this region (encoding phycocyanin or phycoerythrin proteins) are incongruent with trees made with concatenated alignments of ribosomal proteins [29] or core proteins (Figure 4a). Thus, we hypothesize that this region, which is crucial in defining light absorption capacity and, therefore, the optimal light niche of *Synechococcus *genotypes, has been laterally transferred between *Synechococcus *lineages after the major diversification event that has occurred in this group (see below). In this context, it has been suggested that cyanomyoviruses infecting marine *Synechococcus *strains (like S-PM2) may encapsidate randomly selected host DNA fragments having a similar size to the phage genome, that is, 194 kb, and transduce them to another *Synechococcus *strain [39].

### Phylogenomics of marine picocyanobacteria

The availability of numerous complete genomes of marine picocyanobacteria allowed us to refine the phylogenetic relationships between members of this group. An unrooted distance tree using 1,129 concatenated alignments of core proteins is shown in Figure 4a. The same topology is found for parsimony and maximum likelihood (ML) trees as well as for the consensus tree obtained from individual ML trees of core proteins (not shown). This tree shares many characteristics with the 16S rRNA gene tree (Figure 4b), but allows a better resolution of some internal branches. In particular, one can clearly distinguish two main sub-groups within sub-cluster 5.1, one including WH8102, CC9605, CC9902 and BL107 (sub-group A) and the second one including WH7803, WH7805, CC9311, RS9916 and RS9917 (sub-group B), whereas the positions of the latter two strains are uncertain in the 16S rRNA tree. Another important observation emerging from the core protein tree is that RCC307 appears to be located outside sub-cluster 5.1 (with a high bootstrap support), whereas its position is again not well supported in the 16S rRNA gene phylogeny (Figure 4b). Instead, this strain is likely part of a new sub-cluster, which could be called sub-cluster 5.3 (*sensu *[40]), although more genomes from the former clade X [12] are needed to fully support this assignment. The core protein neighbor joining (NJ) tree rooted with the freshwater cyanobacterium *Synechocystis *sp. PCC 6803 (Additional data file 6) suggests that the ancestor of sub-cluster 5.3 diverged before the split between sub-cluster 5.2 and the group gathering sub-cluster 5.1 and all *Prochlorococcus*. Members of sub-cluster 5.1 appear to have quickly differentiated into a number of different clades, as indicated by the short branch lengths at the base of this sub-cluster, and this event has seemingly occurred almost concomitantly with the appearance of the *Prochlorococcus *lineage. This confirms the hypothesis of a rapid diversification of marine picocyanobacteria suggested by Urbach and colleagues [41], based on low bootstrap confidence in the branching of these lineages in 16S rRNA gene trees. This diversification is likely related to the colonization of new marine environments and may partially explain the dominance of *Prochlorococcus *and *Synechococcus *sub-cluster 5.1 over the apparently less diversified sub-clusters 5.2 and 5.3. The differentiation of CC9902 and BL107 (two members of clade IV) on the one hand, and of WH7803 and WH7805 on the other hand, appears to be much more recent.

**Figure 5:**
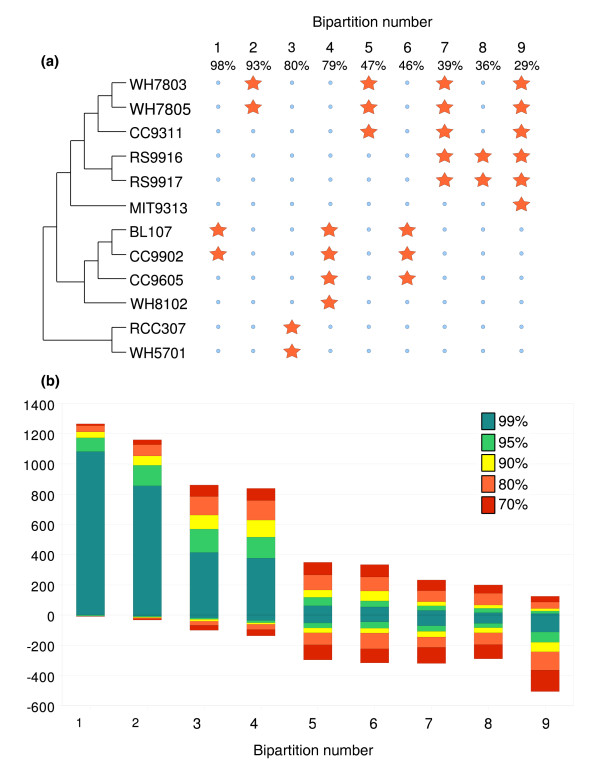
Analyses of bipartition spectra for 12 genomes of marine picocyanobacteria. **(a) **Out of 2,037 bipartitions, 155 were found to be supported with 70% or higher bootstrap values. Percentage values indicate the proportion of gene families that support each consensus bipartition. Only nine consensus bipartitions were found with the Condense software. These bipartitions, represented by orange stars and numbered from 1 to 9, do not conflict with one another and can be combined in a single consensus tree that has the same topology as the tree of core proteins (Figure 4a) except for the position of *Prochlorococcus *sp. MIT9313. Some consensus bipartitions are supported by a low percentage of gene families. This is likely an effect of the rapid divergence between marine *Synechococcus *and *Prochlorococcus *leading to very small internal branches in phylogenetic trees. **(b) **Modified Lento plot for bipartitions with at least 70% bootstrap support. For each bipartition (numbered from 1 to 9), positive values on the y axis give the number of gene families that support the bipartition for a given bootstrap value (color coded). Negative values give the number of families that conflict with this bipartition. A given gene family can conflict with several bipartitions.

Although Figure 4a represents well the evolutionary history of the majority of the core genome (that is, the organism phylogeny), some core genes do not follow this phylogeny, suggesting that they could have been subject to LGT. Using a phylogenetic approach based on the analysis of bipartition spectra [42,43], we identified 122 protein families, including 11 involved in photosynthesis (such as the photosystem I minor subunits PsaL and PsaI, the large subunit of the RubisCo RbcL, several proteins of the Calvin cycle, and so on), that strongly conflict (with bootstrap values higher than 99%) with the bipartitions of the consensus tree (Figure 5 and Additional data file 7). For these protein families, the distorted topology can be explained by at least one transfer of an ortholog from a different lineage followed by the displacement of the original gene by the orthologous copy, which therefore formed a 'xenolog'. Thus, at least 9.3% of the core genes appear to have been laterally transferred between the different *Synechococcus *lineages or between *Synechococcus *and *Prochlorococcus *lineages. An example of such lateral gene transfer, the ferredoxin-dependent glutamate synthase (an enzyme of the GS/GOGAT pathway that is involved in ammonium assimilation), is illustrated in Additional data file 8. This tree suggests that at least two LGTs between clades V and III and between clades IX and II have occurred (Table 1).

### Phyletic patterns

**Figure 6:**
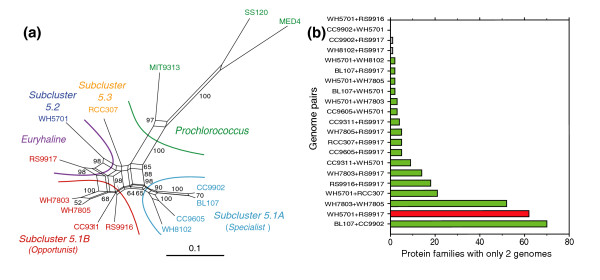
Relationships between genomes based on accessory gene content. **(a) **Phylogenetic network constructed using genes shared by 2-13 genomes with a ML distance estimator and represented as a neighbor net with bootstrap values as implemented by SplitsTree 4.8. **(b) **Number of occurrences of different genome pairs (indicated as 'x+y') among protein families containing only two genomes. Only those pairs including either WH5701 or RS9917 (or both) are shown, as well as the two most related genome pairs BL107/CC9902 and WH7803/WH7805, shown here for comparison.

In order to analyze the relationships between phylogeny based on protein sequences and genome composition further, we constructed a phylogenetic network based on shared gene content (Figure 6a). The relationships between strains in this network are very similar to those observed in the core protein tree (Figure 4a), with the notable exception of the position of RS9917, which clearly groups together with WH5701, indicating that these strains have an unexpected number of genes in common, given their phylogenetic distance. Indeed, WH5701 and RS9917 specifically share almost as many protein families as do the two clade IV strains CC9902 and BL107 and even more than the closely related strains WH7803 and WH7805 (Figure 6b). All other pairs of strains made with either WH5701 or RS9917 have far fewer families in common. Though WH5701 and RS9917 are both euryhaline, examination of the set of 61 protein families specific to both strains (Additional data file 2, lines 403-463) shows that most of them have no known function or general predicted function only, and further characterization (for example by gene knockout) is therefore needed to confirm the potential role of these genes in conferring this specificity. The genes shared by these two strains are notably conserved, however, with a higher level of sequence similarity than with any homolog found in another bacterial lineage. Furthermore, a number of these genes are gathered into islands or smaller clusters, ranging in size from 2-17 genes ('islets'), and with the same gene order in both strains. This suggests that these genes have been transferred between members of sub-cluster 5.2 and clade VIII (5.1B). Finally, these two strains also share a common pigmentation, and this can be attributed to their similar phycobilisome gene complement [29], including two specific phycocyanin rod linkers, CpcC and CpcD (Additional data file 2).

### Towards a better systematics of marine picocyanobacteria

**Figure 7:**
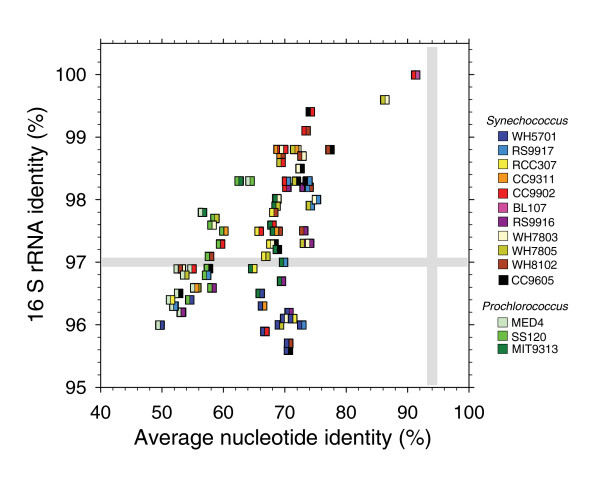
Genome-wide ANI versus percentage of 16S rRNA gene identity. Note that because of the close relatedness between CC9902 and BL107, their respective ANI with any other *Synechococcus *strains are very similar, so only CC9902 is shown on the graph except when compared to BL107 itself.

The availability of numerous complete genome sequences of marine picocyanobacteria provides an opportunity to compare ribotype diversity with protein-coding gene diversity and test the applicability of the bacterial species concept for this set of strains. Although 16S rRNA gene identity is greater than 95.5% across the *Synechococcus *group, the average nucleotide identity (ANI) of genes shared between every pair of genomes is significantly lower than the threshold value of approximately 94%, which, according to Konstantinidis and Tiedje [44], is equivalent to the currently accepted species threshold of 70% DNA-DNA hybridization [45]. Indeed, when considering the picocyanobacterial core proteins, the ANI value ranges from 65.7% between CC9902 (or BL107, clade IV) and RCC307 (clade X) up to only 91.3% between strains BL107 and CC9902 (both clade IV), though the latter strains have identical 16S rRNA gene sequences (Figure 7). ANI values are even lower when considering the larger set of *Synechococcus *core proteins (data not shown). We detected a clear limit (ANI approximately 80-84%) that differentiates *Synechococcus *isolates belonging to the same clade (CC9902/BL107) or to closely related clades (WH7803/WH7805) from members of more divergent clades. In contrast, there is no such clear limit for 16S rRNA gene sequence identity but rather a continuum of values ranging from 95.5-100%. Thus, ANI appears to be a better estimator than 16S rRNA gene identity for assigning strains to a given clade. Nevertheless, one may question whether or not *Synechococcus *'clades' are equivalent to 'species' or 'ecotypes' *sensu *Cohan and Perry [46], that is, groups of microorganisms that are genetically and ecologically similar to one another. BL107 and CC9902, though isolated from very different areas (the California current and the Western Mediterranean Sea, respectively), share the same pigmentation (and both chromatically adapt) and, therefore, likely occupy the same niche, at least with regard to light. In contrast, WH7803 and WH7805 have a different pigmentation (pigment types 2 and 3a, respectively) [29] and, therefore, appear to be genetically adapted to occupy distinct niches. Thus, the first two strains could be members of the same 'species'/'ecotype', whereas the second pair would not, despite the fact that they are 99.6% identical at the 16S rRNA gene sequence level. It must be noted that several clades defined by Fuller and co-workers [18] contain strains that are 100% identical at the 16S rRNA gene sequence level but belong to a different pigment type, so cannot be considered the same 'species'/ecotypes (also see [47]). As a corollary, many currently defined *Synechococcus *'clades' [12-15] might represent some intermediate level between 'species' and 'genus'. If, as we propose, CC9902 and BL107 are members of the same 'species', then the threshold for delineating 'species' in the marine *Synechococcus *complex would lie somewhere between 87 and 91% ANI, that is, lower than for Proteobacteria [44]. This percentage should, however, be refined in future as more *Synechococcus *strains belonging to the same clade and/or species are sequenced.

It is important to note that neither ANI nor 16S rRNA gene sequence identity make it possible to completely resolve the *Prochlorococcus *and *Synechococcus *genera from one another. As a result of their biased GC content and accelerated evolution [22,24,48], *Prochlorococcus *strains with streamlined genomes (SS120 and MED4) fall well apart from *Synechococcus *spp., with ANI values generally below 65% (Figure 7). However, *Prochlorococcus *sp. MIT9313 (and likely other members of this clade such as MIT9303 [23]) cannot be distinguished from *Synechococcus *spp. based on this criterion, since most ANI values between MIT9313 and each of the *Synechococcus *strains range from 64.7-69.7, that is, values not significantly different from those obtained between pairs of *Synechococcus *strains (range 65.7-77.9, excluding the two aforementioned pairs). Nevertheless, it seems taxonomically valid to consider these two genera separately, since representatives of the genus *Prochlorococcus *display a number of unique traits, such as the presence of an intrinsic light-harvesting antenna binding divinyl derivatives of chlorophylls *a *and *b *[8,49,50]. Moreover, even though there are few *Prochlorococcus*-specific genes [23], members of this genus lack 70 protein families common to all the *Synechococcus *strains under study (Additional data file 1).

### Ecological implications

**Figure 8:**
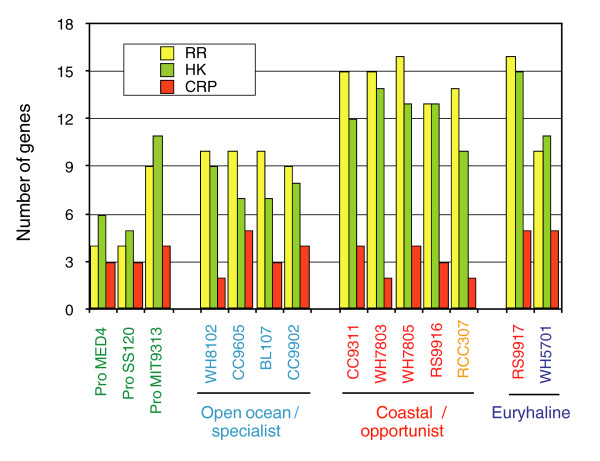
Genome encoded regulatory capacity reflects general life strategies of marine picocyanobacteria. The number of response regulators (RR) and sensor histidine kinases (HK) of two-component regulatory systems, and cAMP-receptor protein (CRP) family regulators encoded in each genome are presented.

The distribution and relative abundance of sub-cluster 5.1 clades in the natural environment suggests the existence of two major lifestyle strategies for marine *Synechococcus*: 'open ocean/specialists' that dominate in warm-oligotrophic or temperate/polar-mesotrophic waters; and 'coastal/opportunists' that can be found either in coastal areas or across a broad range of ecosystems in relatively low numbers, but occasionally reaching higher numbers in the vicinity of upwelling areas or following environmental perturbations [21]. The newly proposed sub-groups A and B within sub-cluster 5.1 may reflect this dichotomy and correspond to ecologically coherent groups. Separation between these two lifestyles, reminiscent of the distinction between HL and LL *Prochlorococcus *clades [10], could have occurred early in the evolutionary history of marine *Synechococcus*. The partition of these two 'eco-groups' is further supported by the number of genes encoding two-component system histidine kinases and response regulators. *Synechococcus *clades II, III, IV (≡ sub-group A), which are prevalent in open-ocean waters, exhibit characteristically low numbers of regulatory systems (Figure 8). In contrast, the regulatory capacity of the sub-group B (clades I, V, VI, VIII IX) and sub-cluster 5.2, which have to cope with much more variable environments, is higher. Future sequencing of environmental genomes should allow confirmation and refinement of the molecular basis of this partitioning.

## Conclusion

Comparative genomics on a large set of *Synechococcus *isolates allowed us to precisely define the core genome and enlightened us to the considerable flexibility of the accessory genome in this group, which is due in large part to a highly variable number of unique genes, preferentially located in islands. The large genomic and physiological diversity occurring between and within current *Synechococcus *'clades' [12-15] suggests the use of smaller, ecologically distinct fundamental units (that is, 'species' or 'ecotypes') for evaluating taxonomic diversity within this group. Since the identification of populations of marine *Synechococcus *adapted to different ecological niches is now relatively well advanced [12,18,19,21,22,51], the incorporation of such ecological data, together with robust DNA sequence clusters resulting from genome comparisons of cultured strains and of environmental isolates, will undoubtedly make it possible to establish an ecologically meaningful systematics for marine picocyanobacteria.

Even though the distribution of *Synechococcus *clades within broad habitats (that is, over large spatial or temporal scales) can be defined using core gene markers, for example, the 16S rRNA gene [19,21] or *rpoC1 *gene [17], adaptation to narrow ecological niches (that is, at local scales) is most likely made possible by the flexibility and/or diversity of the accessory genome. The most obvious illustration of this is the absence of congruence between cell pigmentation and phylogeny that can be related to lateral transfer between *Synechococcus *lineages of the gene region encoding phycobilisome rod components. Thus, horizontal transfer of novel genes (or homologs of existing genes) within genomic islands appears to be a key mechanism for acquiring novel phenotypes and ecological functions. The apparently high turnover of many of these laterally transferred genes increases the probability that they may be useful for cells to adapt to local environmental conditions.

## Materials and methods

### Sequencing, assembly and genome annotation

Whole genome sequencing was performed, starting from DNA of axenic strains or strains with low bacterial contamination, either by the Genoscope for *Synechococcus *spp. WH7803 and RCC307, by the J Craig Venter Institute for WH7805, BL107, RS9916, RS9917 and WH5701, or by the Joint Genome Institute for CC9902 and CC9605, according to the standard protocols used by these different sequencing centers [23,48]. The genome sequences reported in this paper have been deposited in the GenBank database.

### Delineation of protein families

Protein families were delineated using all-against-all BLASTp comparison [52] and the TRIBEMCL clustering algorithm [53] with an e-value threshold of 10^-12^. Open reading frames encoding proteins smaller than 100 amino acids were compared against the whole protein set using a less stringent threshold (10^-5^) and those with significant hits were added to protein families. A number of missing genes were added to the data set by searching intergenic regions with TBLASTN [52] against the whole proteome of the 14 genomes and then against the NCBI non-redundant protein database. An in-house database system (Cyanorak), accessible to all annotators through a web front-end, was set up to manually refine the annotation of protein families. A read-only version of this database is publicly accessible [54].

### Determination of islands

In a previous study [25], islands were identified by breaks in synteny in closely related *Prochlorococcus*. However, this approach was not applicable for the strains analyzed here, due to the simultaneous comparison of multiple genomes and a high background of numerous genomic rearrangements that interrupt synteny. Instead, we used methods modified from Hsiao and co-workers [55] and Rusch and co-workers [56] that were less dependent on genome comparisons. Briefly, gene islands of ≥ 6 genes were suggested by deviation in tetranucleotide frequency greater than 1 standard deviation in the 1st principal component as compared to the genome average. The borders of individual islands were determined with the aid of: proximity of mobility genes (that is, insertion sequence elements or phage integrases) or tRNA genes; and/or the occurrence of blocks of core genes. Finally, a few contiguous blocks of unique and accessory genes that did not display significant deviation in tetranucleotide frequency were manually added to the predicted set of islands for several genomes.

### Phylogenetic analysis

Phylogenetic reconstructions were based on manually aligned full-length 16S rRNA gene sequences using previous alignments [12]. Where possible, 16S rRNA gene sequences were obtained from complete or draft genome sequences, otherwise they were assembled from whole genome shotgun (WGS) sequence reads using Phred, Phrap and Consed [57]. For the 16S rRNA gene phylogenies, the confidence of branch points was determined by three separate analyses (NJ, ML and maximum parsimony), with multifurcations indicating branch points that were collapsed until supported in a majority of analyses.

Core protein families containing only one gene copy per genome (1,129 families) were used to make a refined analysis of the phylogeny of marine picocyanobacteria. Amino acid sequences were aligned using MUSCLE [58] with default parameters. After exclusion of ambiguously aligned regions with Gblocks [59], individual alignments were concatenated in one super-alignment of 307,757 amino acid sites. ML pairwise distances between sequences of the super-alignment were computed with TREE-PUZZLE 5.2 [60] using the JTT model of amino acid substitution and a gamma distribution parameter alpha of 0.34 (estimated from data set). A least-square tree was constructed from the distance matrix using the Fitch program of the Phylip package [61]. Parsimony and ML trees were constructed with Protpars [61] and TREE-PUZZLE, respectively. Bootstrap analyses were performed by sampling 1% of the sites of the original super-alignment to produce 100 alignments of 3,007 positions with the SeqBoot program [61]. Distance, parsimony and ML trees were also constructed for individual alignments of protein families. For ML trees, we used the PhyML program [62] using the JTT model and an alpha parameter estimated from the data set. A majority rule consensus tree was constructed from these individual trees with the Consense program [61]. Gene content phylogeny was built with the phyletic distribution of sequences in orthologous clusters, using genes shared between 2-13 genomes, with the ML estimator of Huson and Steel [63] and bootstrapping of 100 replicates as implemented in SplitsTree 4.8.

Analysis of bipartition spectra was used to detect transfer and replacement of orthologous genes in lineages of marine *Synechococcus *and in *Prochlorococcus *sp. MIT9313. A bipartition corresponds to the splitting of a phylogenetic tree in two parts linked by a single internal branch. ML trees (100 replicates) were built using the PhyML program [62] for 1,317 families of 12 protein-coding genes (that is, excluding *P. marinus *MED4 and SS120). A consensus tree was built with the Consense program from the 1,317 ML trees. Bipartitions supported with 70% or higher bootstrap values were extracted from the set of phylogenetic trees. The method of detection of horizontally transferred genes is based on the identification of protein families showing one or more bipartitions that conflict significantly with plurality bipartitions of the consensus tree.

### Average nucleotide identity between orthologous genes

Pairwise ANI was calculated across the entire genome, as described by Konstantinidis and Tiedje [44], resulting in a majority of values clustered in a narrow band between 70% and 73%. An additional, unrestrained estimate of ANI was calculated across the conserved core component of each genome, with gene families containing paralogs ignored and the minimum blast percentage identity threshold (60%) removed, to provide an alternative estimate of the sequence divergence of this more restricted set of conserved orthologues.

## Abbreviations

ANI, average nucleotide identity; GOS, Global Ocean Sampling; HL, high light; LGT, lateral gene transfer; LL, low light; ML, maximum likelihood; NJ, neighbor joining; WGS, whole genome shotgun.

## Authors' contributions

AD, MO, DJS and FP conceived the study. They also wrote the paper together with LG, NT, BPP, AFP and WRH. MO, SM and BPP grew cultures and provided the DNA used to sequence the nine unpublished *Synechococcus *strains described in this study. PW, CD, JJ and SF worked on the sequencing and/or assembly of, altogether, seven strains, and DJS, FP and BPP coordinated their manual genome annotation. AD performed the clustering of orthologous proteins and set-up a web site for annotating these clusters. AD, MO, DJS, LG, SM, BPP, ITP, NT, AFP, WRH and FP contributed to manual annotation of these protein families. AD and MO did most of the bioinformatic and phylogenetic analyses. All authors read and approved the final manuscript.

## Additional data files

The following additional data files are available with the online version of this paper. Additional data file 1 is a table listing the 70 *Synechococcus*-specific genes. Additional data file 2 is a table listing all accessory protein families found in 2-10 *Synechococcus *strains, including the 61 families shared only by the euryhaline *Synechococcus *spp. strains WH5701 and RS9917. Additional data file 3 shows genome plots of recently acquired islands in the nine genomes not shown in Figures 2 or 3. Additional data file 4 shows an example of a gene island shared by several picocyanobacterial genomes. Additional data file 5 is a table listing island coordinates and island gene composition in the 14 genomes of marine picocyanobacteria used in this study. Additional data file 6 is a NJ tree based on concatenated alignment of the core genome rooted with the freshwater cyanobacterium *Synechocystis *sp. PCC6803 (863 proteins, 263,424 amino acid positions, gene families with paralogs excluded). Additional data file 7 is a table listing the 122 core protein families showing a phylogeny divergent from the consensus core protein distance tree shown in Figure 4a (that is, for which at least one event of LGT has occurred), with bipartition supported by bootstrap values ≥ 99%. Additional data file 8 is an example ML tree of a core gene subjected to LGT, the ferredoxin-dependent glutamate synthase.

## Supplementary Material

Additional data file 1 The 70 *Synechococcus*-specific genes. Click here for file

Additional data file 2 All accessory protein families found in 2-10 *Synechococcus *strains, including the 61 families shared only by the euryhaline *Synechococcus *spp. strains WH5701 and RS9917. Click here for file

Additional data file 3 Genomes have been re-aligned so that they all start at *dnaN*. For other details, see the legend of Figure 3. Click here for file

Additional data file 4 Core genome segments surrounding the islands are connected by yellow shading. Genes shared specifically by *Synechococcus *spp. WH7803 and CC9902 are connected by gray shading. Click here for file

Additional data file 5 Island coordinates and island gene composition in the 14 genomes of marine picocyanobacteria used in this study. Click here for file

Additional data file 6 NJ tree based on concatenated alignment of the core genome rooted with the freshwater cyanobacterium *Synechocystis *sp. PCC6803 (863 proteins, 263,424 amino acid positions, gene families with paralogs excluded). Click here for file

Additional data file 7 The 122 core protein families showing a phylogeny divergent from the consensus core protein distance tree shown in Figure 4a (that is, for which at least one event of LGT has occurred), with bipartition supported by bootstrap values ≥ 99%. Click here for file

Additional data file 8 This enzyme is part of the GS/GOGAT pathway, which is involved in the assimilation of NH_4_^+^. This tree suggests at least two transfers between clades III and V (represented by WH7803 and WH8102, respectively) and between clades II and X (represented by RS9916 and CC9605, respectively). Click here for file

## References

[B1] Goericke R, Welschmeyer NA (1993). The marine prochlorophyte *Prochlorococcus *contributes significantly to phytoplankton biomass and primary production in the Sargasso Sea.. Deep-Sea Res.

[B2] Li WKW (1994). Primary productivity of prochlorophytes, cyanobacteria, and eucaryotic ultraphytoplankton: measurements from flow cytometric sorting.. Limnol Oceanogr.

[B3] Moran XAG, Fernandez E, Perez V (2004). Size-fractionated primary production, bacterial production and net community production in subtropical and tropical domains of the oligotrophic NE Atlantic in autumn.. Mar Ecol Prog Ser.

[B4] Scanlan DJ, West NJ (2002). Molecular ecology of the marine cyanobacterial genera *Prochlorococcus *and *Synechococcus*.. FEMS Microbiol Ecol.

[B5] Olson RJ, Zettler ER, Armbrust EV, Chisholm SW (1990). Pigment, size and distribution of *Synechococcus *in the North Atlantic and Pacific oceans.. Limnol Oceanogr.

[B6] Partensky F, Blanchot J, Vaulot D, Charpy L, Larkum A (1999). Differential distribution and ecology of *Prochlorococcus *and *Synechococcus *in oceanic waters: a review.. Marine Cyanobacteria.

[B7] Olson RJ, Zettler ER, Altabet MA, Dusenberry JA, Chisholm SW (1990). Spatial and temporal distributions of prochlorophyte picoplankton in the North Atlantic Ocean.. Deep-Sea Res.

[B8] Partensky F, Hess WR, Vaulot D (1999). *Prochlorococcus*, a marine photosynthetic prokaryote of global significance.. Microbiol Mol Biol Rev.

[B9] Tarran GA, Zubkov MV, Sleigh MA, Burkill PH, Yallop M (2001). Microbial community structure and standing stocks in the NE Atlantic in June and July of 1996.. Deep-Sea Res II.

[B10] Moore LR, Rocap G, Chisholm SW (1998). Physiology and molecular phylogeny of coexisting *Prochlorococcus *ecotypes.. Nature.

[B11] Johnson ZI, Zinser ER, Coe A, McNulty NP, Woodward EM, Chisholm SW (2006). Niche partitioning among *Prochlorococcus *ecotypes along ocean-scale environmental gradients.. Science.

[B12] Fuller NJ, Marie D, Partensky F, Vaulot D, Post AF, Scanlan DJ (2003). Clade-specific 16S ribosomal DNA oligonucleotides reveal the predominance of a single marine *Synechococcus *clade throughout a stratified water column in the Red Sea.. Appl Environ Microbiol.

[B13] Ahlgren NA, Rocap G (2006). Culture isolation and culture-independent clone libraries reveal new marine *Synechococcus *ecotypes with distinctive light and N physiologies.. Appl Environ Microbiol.

[B14] Muhling M, Fuller NJ, Somerfield PJ, Post AF, Wilson WH, Scanlan DJ, Joint I, Mann NH (2006). High resolution genetic diversity studies of marine *Synechococcus *isolates using *rpoC1*-based restriction fragment length polymorphism.. Aquat Microb Ecol.

[B15] Penno S, Lindell D, Post AF (2006). Diversity of *Synechococcus *and *Prochlorococcus *populations determined from DNA sequences of the N-regulatory gene *ntcA*.. Environ Microbiol.

[B16] Toledo G, Palenik B, Brahamsha B (1999). Swimming marine *Synechococcus *strains with widely different photosynthetic pigment ratios form a monophyletic group.. Appl Environ Microbiol.

[B17] Ferris MJ, Palenik B (1998). Niche adaptation in ocean cyanobacteria.. Nature.

[B18] Fuller NJ, Tarran GA, Yallop M, Orcutt KM, Scanlan DJ (2006). Molecular analysis of picocyanobacterial community structure along an Arabian Sea transect reveals distinct spatial separation of lineages.. Limnol Oceanogr.

[B19] Zwirglmaier K, Heywood JL, Chamberlain K, Woodward EMS, Zubkov MV, Scanlan DJ (2007). Basin-scale distribution patterns of picocyanobacterial lineages in the Atlantic Ocean.. Environ Microbiol.

[B20] Toledo G, Palenik B (2003). A *Synechococcus *serotype is found preferentially in surface marine waters.. Limnol Oceanogr.

[B21] Zwirglmaier K, Jardillier L, Ostrowski M, Mazard S, Garczarek L, Vaulot D, Not F, Massana R, Ulloa O, Scanlan DJ (2008). Global phylogeography of marine *Synechococcus *and *Prochlorococcus *reveals a distinct partitioning of lineages among oceanic biomes.. Environ Microbiol.

[B22] Rocap G, Larimer FW, Lamerdin J, Malfatti S, Chain P, Ahlgren NA, Arellano A, Coleman M, Hauser L, Hess WR, Johnson ZI, Land M, Lindell D, Post AF, Regala W, Shah M, Shaw SL, Steglich C, Sullivan MB, Ting CS, Tolonen A, Webb EA, Zinser ER, Chisholm SW (2003). Genome divergence in two *Prochlorococcus *ecotypes reflects oceanic niche differentiation.. Nature.

[B23] Kettler G, Martiny AC, Huang K, Zucker J, Coleman ML, Rodrigue S, Chen F, Lapidus A, Ferriera S, Johnson J, Steglich C, Church G, Richardson P, Chisholm SW (2007). Patterns and implications of gene gain and loss in the evolution of *Prochlorococcus*.. PLoS Genet.

[B24] Dufresne A, Garczarek L, Partensky F (2005). Accelerated evolution associated with genome reduction in a free-living prokaryote.. Genome Biol.

[B25] Coleman ML, Sullivan MB, Martiny AC, Steglich C, Barry K, Delong EF, Chisholm SW (2006). Genomic islands and the ecology and evolution of *Prochlorococcus*.. Science.

[B26] Palenik B, Brahamsha B, Larimer FW, Land M, Hauser L, Chain P, Lamerdin J, Regala W, Allen EE, McCarren J, Paulsen I, Dufresne A, Partensky F, Webb EA, Waterbury J (2003). The genome of a motile marine *Synechococcus*.. Nature.

[B27] Palenik B, Ren QH, Dupont CL, Myers GS, Heidelberg JF, Badger JH, Madupu R, Nelson WC, Brinkac LM, Dodson RJ, Durkin AS, Daugherty SC, Sullivan SA, Khouri H, Mohamoud Y, Halpin R, Paulsen IT (2006). Genome sequence of *Synechococcus *CC9311: Insights into adaptation to a coastal environment.. Proc Natl Acad Sci USA.

[B28] Mulkidjanian AY, Koonin EV, Makarova KS, Mekhedov SL, Sorokin A, Wolf YI, Dufresne A, Partensky F, Burd H, Kaznadzey D, Haselkorn R, Galperin MY (2006). The cyanobacterial genome core and the origin of photosynthesis.. Proc Natl Acad Sci USA.

[B29] Six C, Thomas J-C, Garczarek L, Ostrowski M, Dufresne A, Blot N, Scanlan DJ, Partensky F (2007). Diversity and evolution of phycobilisomes in marine *Synechococcus *spp. - a comparative genomics study.. Genome Biol.

[B30] Hess WR, Rocap G, Ting CS, Larimer F, Stilwagen S, Lamerdin J, Chisholm SW (2001). The photosynthetic apparatus of *Prochlorococcus*: Insights through comparative genomics.. Photosynth Res.

[B31] Steglich C, Frankenberg-Dinkel N, Penno S, Hess WR (2005). A green light-absorbing phycoerythrin is present in the high-light-adapted marine cyanobacterium *Prochlorococcus *sp. MED4.. Environ Microbiol.

[B32] Badger MR, Price GD (2003). CO2 concentrating mechanisms in cyanobacteria: molecular components, their diversity and evolution.. J Exp Bot.

[B33] Schürmann P, Jacquot JP (2000). Plant thioredoxin systems revisited.. Ann Rev Plant Physiol Plant Mol Biol.

[B34] Geiss U, Vinnemeier J, Kunert A, Lindner I, Gemmer B, Lorenz M, Hagemann M, Schoor A (2001). Detection of the *isiA *gene across cyanobacterial strains: Potential for probing iron deficiency.. Appl Environ Microbiol.

[B35] Zurbriggen MD, Tognetti VB, Carrillo N (2007). Stress-inducible flavodoxin from photosynthetic microorganisms. The mystery of flavodoxin loss from the plant genome.. Iubmb Life.

[B36] Erdner DL, Price NM, Doucette GJ, Peleato ML, Anderson DM (1999). Characterization of ferredoxin and flavodoxin as markers of iron limitation in marine phytoplankton.. Mar Ecol Progr Ser.

[B37] Global Ocean Sampling (GOS) Web Site. http://www.jcvi.org/cms/research/projects/gos.

[B38] McCarren J, Brahamsha B (2007). SwmB, a 1.12-megadalton protein that is required for nonflagellar swimming motility in *Synechococcus*.. J Bacteriol.

[B39] Clokie MR, Millard AD, Wilson WH, Mann NH (2003). Encapsidation of host DNA by bacteriophages infecting marine *Synechococcus *strains.. FEMS Microbiol Ecol.

[B40] Herdman M, Castenholz RW, Waterbury JB, Rippka R, Boone DR, Castenholz RW (2001). Form-genus XIII. *Synechococcus*.. Bergey's Manual of Systematic Bacteriology.

[B41] Urbach E, Scanlan DJ, Distel DL, Waterbury JB, Chisholm SW (1998). Rapid diversification of marine picophytoplankton with dissimilar light-harvesting structures inferred from sequences of *Prochlorococcus *and *Synechococcus *(Cyanobacteria).. J Mol Evol.

[B42] Lento GM, Hickson RE, Chambers GK, Penny D (1995). Use of spectral analysis to test hypotheses on the origin of pinnipeds.. Mol Biol Evol.

[B43] Zhaxybayeva O, Lapierre P, Gogarten JP (2004). Genome mosaicism and organismal lineages.. Trends Genet.

[B44] Konstantinidis KT, Tiedje JM (2005). Genomic insights that advance the species definition for prokaryotes.. Proc Natl Acad Sci USA.

[B45] Wayne LG, Brenner DJ, Colwell RR, Grimont PAD, Kandler O, Krichevsky MI, Moore LH, Murray RGE, Stackebrandt E, Starr MP, Trüper HG (1987). Report of the *ad hoc *commitee on reconciliation of approaches to bacterial systematics.. Intl J Syst Bacteriol.

[B46] Cohan FM, Perry EB (2007). A systematics for discovering the fundamental units of bacterial diversity.. Curr Biol.

[B47] Haverkamp T, Acinas SG, Doeleman M, Stomp M, Huisman J, Stal LJ (2008). Diversity and phylogeny of Baltic Sea picocyanobacteria inferred from their ITS and phycobiliprotein operons.. Environ Microbiol.

[B48] Dufresne A, Salanoubat M, Partensky F, Artiguenave F, Axmann IM, Barbe V, Duprat S, Galperin MY, Koonin EV, Le Gall F, Makarova KS, Ostrowski M, Oztas S, Robert C, Rogozin IB, Scanlan DJ, Tandeau de Marsac N, Weissenbach J, Wincker P, Wolf YI, Hess WR (2003). Genome sequence of the cyanobacterium *Prochlorococcus marinus *SS120, a nearly minimal oxyphototrophic genome.. Proc Natl Acad Sci USA.

[B49] Bibby TS, Nield J, Chen M, Larkum AW, Barber J (2003). Structure of a photosystem II supercomplex isolated from *Prochloron didemni *retaining its chlorophyll *a/b *light-harvesting system.. Proc Natl Acad Sci USA.

[B50] Garczarek L, Dufresne A, Rousvoal S, West NJ, Mazard S, Marie D, Claustre H, Raimbault P, Post AF, Scanlan DJ, Partensky F (2007). High vertical and low horizontal diversity of *Prochlorococcus *ecotypes in the Mediterranean Sea in summer.. FEMS Microbiol Ecol.

[B51] Rocap G, Distel DL, Waterbury JB, Chisholm SW (2002). Resolution of *Prochlorococcus *and *Synechococcus *ecotypes by using 16S-23S ribosomal DNA internal transcribed spacer sequences.. Appl Environ Microbiol.

[B52] Altschul SF, Madden TL, Schaffer AA, Zhang J, Zhang Z, Miller W, Lipman DJ (1997). Gapped BLAST and PSI-BLAST: a new generation of protein database search programs.. Nucleic Acids Res.

[B53] Enright AJ, van Dongen S, Ouzounis CA (2002). An efficient algorithm for large-scale detection of protein families.. Nucleic Acids Res.

[B54] Cyanorak Database. http://www.sb-roscoff.fr/Phyto/cyanorak.

[B55] Hsiao WWL, Ung K, Aeschliman D, Bryan J, Finlay BB, Brinkman FSL (2005). Evidence of a large novel gene pool associated with prokaryotic genomic islands.. PLoS Genet.

[B56] Rusch DB, Halpern AL, Sutton G, Heidelberg KB, Williamson S, Yooseph S, Wu D, Eisen JA, Hoffman JM, Remington K, Beeson K, Tran B, Smith H, Baden-Tillson H, Stewart C, Thorpe J, Freeman J, Andrews-Pfannkoch C, Venter JE, Li K, Kravitz S, Heidelberg JF, Utterback T, Rogers Y-H, Falcon LI, Souza V, Bonilla-Rosso G, Eguiarte LE, Karl DM, Sathyendranath S (2007). The Sorcerer II global ocean sampling expedition: Northwest Atlantic through eastern tropical Pacific.. PLoS Biol.

[B57] Phrap, Phred, Consed Web Site. http://www.phrap.org/phredphrapconsed.html.

[B58] Edgar RC (2004). MUSCLE: multiple sequence alignment with high accuracy and high throughput.. Nucleic Acids Res.

[B59] Castresana J (2000). Selection of conserved blocks from multiple alignments for their use in phylogenetic analysis.. Mol Biol Evol.

[B60] Schmidt HA, Strimmer K, Vingron M, von Haeseler A (2002). TREE-PUZZLE: maximum likelihood phylogenetic analysis using quartets and parallel computing.. Bioinformatics.

[B61] Felsenstein J (1989). PHYLIP - Phylogeny inference package (version 3.2).. Cladistics.

[B62] Guindon S, Gascuel O (2003). A simple, fast, and accurate algorithm to estimate large phylogenies by maximum likelihood.. Syst Biol.

[B63] Huson DH, Steel M (2004). Phylogenetic trees based on gene content.. Bioinformatics.

