# Supplementary material for: Unraveling the genomic mosaic of a ubiquitous genus of marine cyanobacteria
Source: Genome Biol. 2008 May 28;9(5):R90. doi: 10.1186/gb-2008-9-5-r90 (PMC2441476; doi:10.1186/gb-2008-9-5-r90)
Supplement: Additional data file 1 — The 70 Synechococcus-specific genes. [file gb-2008-9-5-r90-S1.pdf]

						Synechococcus											Prochlorococcus		
						Subcluster 5.1A					Subcluster 5.1B				5.2	5.3	HL	LL	
Line No.	Cluster No. in cyanorak	Gene Name	Product	No. of *Synechococcus* strains per cluster	No. of sequ. per cluster (14 genomes)	BL107	CC9902	CC9605	WH8102	CC9311	WH7803	WH7805	RS9916	RS9917	WH5701	RCC307	MED4	SS120	MIT9313
1	8006	*apcA*	Allophycocyanin, alpha chain	11	11	1	1	1	1	1	1	1	1	1	1	1	0	0	0
2	8007	*apcB*	Allophycocyanin, beta chain	11	11	1	1	1	1	1	1	1	1	1	1	1	0	0	0
3	1646	*apcC*	Phycobilisome 7.8 kDa linker polypeptide, allophycocyanin-associated	11	11	1	1	1	1	1	1	1	1	1	1	1	0	0	0
4	8008	*apcD*	Allophycocyanin alpha-B chain	11	11	1	1	1	1	1	1	1	1	1	1	1	0	0	0
5	1645	*apcE*	Phycobilisome core-membrane linker polypeptide (LCM)	11	11	1	1	1	1	1	1	1	1	1	1	1	0	0	0
6	4	*apcF*	Allophycocyanin, beta-18 chain	11	11	1	1	1	1	1	1	1	1	1	1	1	0	0	0
7	9072	*cpcGI*	Phycobilisome rod-core linker polypeptide CpcGI	11	11	1	1	1	1	1	1	1	1	1	1	1	0	0	0
8	44	*cpcGII*	Phycobilisome rod-core linker polypeptide CpcGII	11	11	1	1	1	1	1	1	1	1	1	1	1	0	0	0
9	1528	*cpcS*	Allophycocyanin and phycocyanin Cys-84 phycocyanobilin lyase	11	12	1	1	1	1	1	1	1	1	1	2	1	0	0	0
10	1547		Phycobiliprotein lyase or activator, similar to CotB	11	11	1	1	1	1	1	1	1	1	1	1	1	0	0	0
11	1699		Carbonic anhydrase/acetyltransferase	11	11	1	1	1	1	1	1	1	1	1	1	1	0	0	0
12	1423	*chpX*	CO2 hydration protein	11	12	1	1	1	1	1	1	1	1	1	2	1	0	0	0
13	8090	*ndhD*	NADH dehydrogenase I subunit, NdhD4 homolog	11	12	1	1	1	1	1	1	1	1	1	2	1	0	0	0
14	1422	*ndhF*	NADH dehydrogenase I subunit, NdhF4 homolog	11	12	1	1	1	1	1	1	1	1	1	2	1	0	0	0
15	1574		Glycerol-3-phosphate dehydrogenase	11	11	1	1	1	1	1	1	1	1	1	1	1	0	0	0
16	1648	*ftrC*	Ferredoxin-thioredoxin reductase, catalytic subunit	11	11	1	1	1	1	1	1	1	1	1	1	1	0	0	0
17	1644	*ftrV*	Ferredoxin-thioredoxin reductase, variable chain	11	11	1	1	1	1	1	1	1	1	1	1	1	0	0	0
18	1583		Ferredoxin	11	11	1	1	1	1	1	1	1	1	1	1	1	0	0	0
19	47	*trxA*	Thioredoxin	11	11	1	1	1	1	1	1	1	1	1	1	1	0	0	0
20	67	*hli*	High light inducible protein	11	12	1	1	1	1	1	1	1	1	1	2	1	0	0	0
21	969	*hli*	High light inducible protein	11	16	1	1	1	1	3	2	1	1	1	2	2	0	0	0
22	1609	*hli*	High light inducible protein	11	11	1	1	1	1	1	1	1	1	1	1	1	0	0	0
23	9	*psbA*	Photosystem II protein D1.1	11	36	4	3	3	2	3	3	5	4	3	3	3	0	0	0
24	1641	*kaiA*	Circadian clock protein KaiA	11	11	1	1	1	1	1	1	1	1	1	1	1	0	0	0
25	1565	*thyX*	Alternative thymidylate synthase	11	11	1	1	1	1	1	1	1	1	1	1	1	0	0	0
26	1628	*acyP*	Acylphosphatase	11	11	1	1	1	1	1	1	1	1	1	1	1	0	0	0
27	1584		Deoxyribodipyrimidine photolyase	11	11	1	1	1	1	1	1	1	1	1	1	1	0	0	0
28	1540		FAD binding domain of DNA photolyase	11	11	1	1	1	1	1	1	1	1	1	1	1	0	0	0
29	1529		Phosphatidylcholine-hydrolyzing phospholipase D family protein	11	12	1	1	1	1	1	1	2	1	1	1	1	0	0	0
30	1328		Phosphorylase kinase alpha/beta	11	12	1	1	1	1	1	1	1	1	1	2	1	0	0	0
31	1610	*ggpS*	Glucosylglycerol-phosphate synthase	11	11	1	1	1	1	1	1	1	1	1	1	1	0	0	0
32	1575		Glycerol kinase	11	12	1	1	1	1	1	1	2	1	1	1	1	0	0	0
33	1404		trehalose synthase	11	11	1	1	1	1	1	1	1	1	1	1	1	0	0	0
34	1573		Possible universal stress protein with 2 USP-like domains	11	11	1	1	1	1	1	1	1	1	1	1	1	0	0	0
35	1797		Possible cation efflux transporter (CDF family)	11	11	1	1	1	1	1	1	1	1	1	1	1	0	0	0
36	18		Possible RND family multidrug efflux transporter	11	11	1	1	1	1	1	1	1	1	1	1	1	0	0	0
37	1561		Molecular chaperone (small heat shock protein)	11	11	1	1	1	1	1	1	1	1	1	1	1	0	0	0
38	1593		Two-component sytem response regulator	11	11	1	1	1	1	1	1	1	1	1	1	1	0	0	0
39	1537		Conserved hypothetical membrane protein	11	11	1	1	1	1	1	1	1	1	1	1	1	0	0	0
40	1568		Conserved hypothetical membrane protein	11	11	1	1	1	1	1	1	1	1	1	1	1	0	0	0
41	1578		Conserved hypothetical membrane protein	11	11	1	1	1	1	1	1	1	1	1	1	1	0	0	0
42	1581		Conserved hypothetical membrane protein	11	11	1	1	1	1	1	1	1	1	1	1	1	0	0	0
43	1639		Conserved hypothetical membrane protein	11	11	1	1	1	1	1	1	1	1	1	1	1	0	0	0
44	1647		Conserved hypothetical membrane protein	11	11	1	1	1	1	1	1	1	1	1	1	1	0	0	0
45	1909		Conserved hypothetical membrane protein	11	11	1	1	1	1	1	1	1	1	1	1	1	0	0	0
46	1545		Conserved hypothetical membrane protein	11	11	1	1	1	1	1	1	1	1	1	1	1	0	0	0
47	1541		Conserved hypothetical protein	11	11	1	1	1	1	1	1	1	1	1	1	1	0	0	0
48	1550		Conserved hypothetical protein	11	11	1	1	1	1	1	1	1	1	1	1	1	0	0	0
49	1567		Conserved hypothetical protein	11	11	1	1	1	1	1	1	1	1	1	1	1	0	0	0
50	1569		Conserved hypothetical protein	11	11	1	1	1	1	1	1	1	1	1	1	1	0	0	0
51	1586		Conserved hypothetical protein	11	11	1	1	1	1	1	1	1	1	1	1	1	0	0	0
52	1592		Conserved hypothetical protein	11	11	1	1	1	1	1	1	1	1	1	1	1	0	0	0
53	1594		Conserved hypothetical protein	11	13	1	1	1	2	1	1	2	1	1	1	1	0	0	0
54	1597		Conserved hypothetical protein	11	11	1	1	1	1	1	1	1	1	1	1	1	0	0	0
55	1607		Conserved hypothetical protein	11	11	1	1	1	1	1	1	1	1	1	1	1	0	0	0
56	1611		Conserved hypothetical protein	11	11	1	1	1	1	1	1	1	1	1	1	1	0	0	0
57	1613		Conserved hypothetical protein	11	11	1	1	1	1	1	1	1	1	1	1	1	0	0	0
58	1631		Conserved hypothetical protein	11	11	1	1	1	1	1	1	1	1	1	1	1	0	0	0
59	1638		Conserved hypothetical protein	11	11	1	1	1	1	1	1	1	1	1	1	1	0	0	0
60	1643		Conserved hypothetical protein	11	11	1	1	1	1	1	1	1	1	1	1	1	0	0	0
61	1721		Conserved hypothetical protein	11	11	1	1	1	1	1	1	1	1	1	1	1	0	0	0
62	1795		Conserved hypothetical protein	11	11	1	1	1	1	1	1	1	1	1	1	1	0	0	0
63	2054		Conserved hypothetical protein	11	11	1	1	1	1	1	1	1	1	1	1	1	0	0	0
64	2058		Conserved hypothetical protein	11	11	1	1	1	1	1	1	1	1	1	1	1	0	0	0
65	2066		Conserved hypothetical protein	11	11	1	1	1	1	1	1	1	1	1	1	1	0	0	0
66	2133		Conserved hypothetical protein	11	11	1	1	1	1	1	1	1	1	1	1	1	0	0	0
67	2205		Conserved hypothetical protein	11	11	1	1	1	1	1	1	1	1	1	1	1	0	0	0
68	2915		Conserved hypothetical protein	11	11	1	1	1	1	1	1	1	1	1	1	1	0	0	0
69	1977		Conserved hypothetical protein	11	11	1	1	1	1	1	1	1	1	1	1	1	0	0	0
70	1591		Conserved hypothetical protein	11	11	1	1	1	1	1	1	1	1	1	1	1	0	0	0

Green background: genes linked to photosynthesis
