# Supplementary material for: Unraveling the genomic mosaic of a ubiquitous genus of marine cyanobacteria
Source: Genome Biol. 2008 May 28;9(5):R90. doi: 10.1186/gb-2008-9-5-r90 (PMC2441476; doi:10.1186/gb-2008-9-5-r90)
Supplement: Additional data file 2 — All accessory protein families found in 2-10 Synechococcus strains, including the 61 families shared only by the euryhaline Synechococcus spp. strains WH5701 and RS9917. [file gb-2008-9-5-r90-S2.pdf]

Synechococcus accessory genome (minus unique genes)

						Synechococcus											Prochlorococcus		
						Subcluster 5.1A					Subcluster 5.1B				5.2	5.3	HL		LL
Line No.	Cluster No. in cyanorak	Gene Name	Product	No. of *Synechococcus* strains per cluster	No. of sequ. per cluster (14 genomes)	BL107	CC9902	CC9605	WH8102	CC9311	WH7803	WH7805	RS9916	RS9917	WH5701	RCC307	MED4	SS120	MIT9313
1	6479		Conserved hypothetical membrane protein	2	2	1	1	0	0	0	0	0	0	0	0	0	0	0	0
2	6505		Conserved hypothetical membrane protein	2	2	1	1	0	0	0	0	0	0	0	0	0	0	0	0
3	6517		Conserved hypothetical membrane protein	2	2	1	1	0	0	0	0	0	0	0	0	0	0	0	0
4	6519		Conserved hypothetical membrane protein	2	2	1	1	0	0	0	0	0	0	0	0	0	0	0	0
5	2521		Conserved hypothetical protein	2	2	1	1	0	0	0	0	0	0	0	0	0	0	0	0
6	2533		Conserved hypothetical protein	2	2	1	1	0	0	0	0	0	0	0	0	0	0	0	0
7	2538		Conserved hypothetical protein	2	2	1	1	0	0	0	0	0	0	0	0	0	0	0	0
8	2544		Conserved hypothetical protein	2	2	1	1	0	0	0	0	0	0	0	0	0	0	0	0
9	2547		Conserved hypothetical protein	2	2	1	1	0	0	0	0	0	0	0	0	0	0	0	0
10	2550		Conserved hypothetical protein	2	2	1	1	0	0	0	0	0	0	0	0	0	0	0	0
11	2553		Conserved hypothetical protein	2	2	1	1	0	0	0	0	0	0	0	0	0	0	0	0
12	2561		Conserved hypothetical protein	2	2	1	1	0	0	0	0	0	0	0	0	0	0	0	0
13	2563		Conserved hypothetical protein	2	2	1	1	0	0	0	0	0	0	0	0	0	0	0	0
14	2564		Conserved hypothetical protein	2	2	1	1	0	0	0	0	0	0	0	0	0	0	0	0
15	2571		Conserved hypothetical protein	2	2	1	1	0	0	0	0	0	0	0	0	0	0	0	0
16	2572		Conserved hypothetical protein	2	2	1	1	0	0	0	0	0	0	0	0	0	0	0	0
17	2575		Conserved hypothetical protein	2	2	1	1	0	0	0	0	0	0	0	0	0	0	0	0
18	2577		Conserved hypothetical protein	2	2	1	1	0	0	0	0	0	0	0	0	0	0	0	0
19	2579		Conserved hypothetical protein	2	2	1	1	0	0	0	0	0	0	0	0	0	0	0	0
20	2580		Conserved hypothetical protein	2	2	1	1	0	0	0	0	0	0	0	0	0	0	0	0
21	2583		Conserved hypothetical protein	2	2	1	1	0	0	0	0	0	0	0	0	0	0	0	0
22	2588		Conserved hypothetical protein	2	2	1	1	0	0	0	0	0	0	0	0	0	0	0	0
23	2589		Conserved hypothetical protein	2	2	1	1	0	0	0	0	0	0	0	0	0	0	0	0
24	2590		Conserved hypothetical protein	2	2	1	1	0	0	0	0	0	0	0	0	0	0	0	0
25	2595		Conserved hypothetical protein	2	2	1	1	0	0	0	0	0	0	0	0	0	0	0	0
26	2597		Conserved hypothetical protein	2	2	1	1	0	0	0	0	0	0	0	0	0	0	0	0
27	2598		Conserved hypothetical protein	2	2	1	1	0	0	0	0	0	0	0	0	0	0	0	0
28	2599		Conserved hypothetical protein	2	2	1	1	0	0	0	0	0	0	0	0	0	0	0	0
29	2602		Conserved hypothetical protein	2	2	1	1	0	0	0	0	0	0	0	0	0	0	0	0
30	2603		Conserved hypothetical protein	2	2	1	1	0	0	0	0	0	0	0	0	0	0	0	0
31	2608		Conserved hypothetical protein	2	2	1	1	0	0	0	0	0	0	0	0	0	0	0	0
32	2609		Conserved hypothetical protein	2	2	1	1	0	0	0	0	0	0	0	0	0	0	0	0
33	2611		Conserved hypothetical protein	2	2	1	1	0	0	0	0	0	0	0	0	0	0	0	0
34	2613		Conserved hypothetical protein	2	2	1	1	0	0	0	0	0	0	0	0	0	0	0	0
35	2617		Conserved hypothetical protein	2	2	1	1	0	0	0	0	0	0	0	0	0	0	0	0
36	2621		Conserved hypothetical protein	2	2	1	1	0	0	0	0	0	0	0	0	0	0	0	0
37	2623		Conserved hypothetical protein	2	2	1	1	0	0	0	0	0	0	0	0	0	0	0	0
38	3107		Conserved hypothetical protein	2	2	1	1	0	0	0	0	0	0	0	0	0	0	0	0
39	3172		Conserved hypothetical protein	2	2	1	1	0	0	0	0	0	0	0	0	0	0	0	0
40	3179		Conserved hypothetical protein	2	2	1	1	0	0	0	0	0	0	0	0	0	0	0	0
41	3190		Conserved hypothetical protein	2	2	1	1	0	0	0	0	0	0	0	0	0	0	0	0
42	3193		Conserved hypothetical protein	2	2	1	1	0	0	0	0	0	0	0	0	0	0	0	0
43	3208		Conserved hypothetical protein	2	2	1	1	0	0	0	0	0	0	0	0	0	0	0	0
44	3227		Conserved hypothetical protein	2	2	1	1	0	0	0	0	0	0	0	0	0	0	0	0
45	3235		Conserved hypothetical protein	2	2	1	1	0	0	0	0	0	0	0	0	0	0	0	0
46	3237		Conserved hypothetical protein	2	2	1	1	0	0	0	0	0	0	0	0	0	0	0	0
47	3242		Conserved hypothetical protein	2	2	1	1	0	0	0	0	0	0	0	0	0	0	0	0
48	3250		Conserved hypothetical protein	2	2	1	1	0	0	0	0	0	0	0	0	0	0	0	0
49	3251		Conserved hypothetical protein	2	2	1	1	0	0	0	0	0	0	0	0	0	0	0	0
50	3261		Conserved hypothetical protein	2	2	1	1	0	0	0	0	0	0	0	0	0	0	0	0
51	3264		Conserved hypothetical protein	2	2	1	1	0	0	0	0	0	0	0	0	0	0	0	0
52	3265		Conserved hypothetical protein	2	2	1	1	0	0	0	0	0	0	0	0	0	0	0	0
53	3275		Conserved hypothetical protein	2	2	1	1	0	0	0	0	0	0	0	0	0	0	0	0
54	3279		Conserved hypothetical protein	2	2	1	1	0	0	0	0	0	0	0	0	0	0	0	0
55	3280		Conserved hypothetical protein	2	2	1	1	0	0	0	0	0	0	0	0	0	0	0	0
56	3284		Conserved hypothetical protein	2	2	1	1	0	0	0	0	0	0	0	0	0	0	0	0
57	3285		Conserved hypothetical protein	2	2	1	1	0	0	0	0	0	0	0	0	0	0	0	0
58	3293		Conserved hypothetical protein	2	2	1	1	0	0	0	0	0	0	0	0	0	0	0	0
59	3296		Conserved hypothetical protein	2	2	1	1	0	0	0	0	0	0	0	0	0	0	0	0
60	3298		Conserved hypothetical protein	2	2	1	1	0	0	0	0	0	0	0	0	0	0	0	0
61	3300		Conserved hypothetical protein	2	2	1	1	0	0	0	0	0	0	0	0	0	0	0	0
62	3310		Conserved hypothetical protein	2	2	1	1	0	0	0	0	0	0	0	0	0	0	0	0
63	3316		Conserved hypothetical protein	2	2	1	1	0	0	0	0	0	0	0	0	0	0	0	0
64	3317		Conserved hypothetical protein	2	2	1	1	0	0	0	0	0	0	0	0	0	0	0	0
65	3321		Conserved hypothetical protein	2	2	1	1	0	0	0	0	0	0	0	0	0	0	0	0
66	3322		Conserved hypothetical protein	2	2	1	1	0	0	0	0	0	0	0	0	0	0	0	0
67	3329		Conserved hypothetical protein	2	2	1	1	0	0	0	0	0	0	0	0	0	0	0	0
68	3331		Conserved hypothetical protein	2	2	1	1	0	0	0	0	0	0	0	0	0	0	0	0
69	3337		Conserved hypothetical protein	2	2	1	1	0	0	0	0	0	0	0	0	0	0	0	0
70	2559		Two component system sensor histidine kinase	2	2	1	1	0	0	0	0	0	0	0	0	0	0	0	0
71	3150		AraC-type DNA-binding domain-containing proteins	2	2	1	0	0	0	1	0	0	0	0	0	0	0	0	0
72	2285		Conserved hypothetical protein	2	3	1	0	2	0	0	0	0	0	0	0	0	0	0	0
73	2524		Conserved hypothetical protein	2	2	1	0	1	0	0	0	0	0	0	0	0	0	0	0
74	2566		Conserved hypothetical protein	2	2	1	0	1	0	0	0	0	0	0	0	0	0	0	0
75	2620		Conserved hypothetical protein	2	2	1	0	1	0	0	0	0	0	0	0	0	0	0	0
76	3185		Conserved hypothetical protein	2	2	1	0	1	0	0	0	0	0	0	0	0	0	0	0
77	2531		conserved hypothetical protein distantly related to sialidases	2	2	1	0	1	0	0	0	0	0	0	0	0	0	0	0
78	2569		DNA breaking-rejoining enzymes, integrase/recombinase, C-terminal catalytic domain	2	2	1	0	1	0	0	0	0	0	0	0	0	0	0	0
79	2568		DNA-directed RNA polymerase	2	2	1	0	1	0	0	0	0	0	0	0	0	0	0	0
80	9132		Possible beta-glycosyltransferase, family 2	2	2	1	0	1	0	0	0	0	0	0	0	0	0	0	0
81	2567		Similar to RecB ATP-dependent exoDNAse (exonuclease V) beta subunit	2	2	1	0	1	0	0	0	0	0	0	0	0	0	0	0
82	2526		Conserved hypothetical protein	2	2	1	0	0	1	0	0	0	0	0	0	0	0	0	0
83	2570		Conserved hypothetical protein	2	2	1	0	0	1	0	0	0	0	0	0	0	0	0	0
84	2585		Conserved hypothetical protein	2	2	1	0	0	1	0	0	0	0	0	0	0	0	0	0
85	2605		Conserved hypothetical protein	2	2	1	0	0	1	0	0	0	0	0	0	0	0	0	0
86	2614		Conserved hypothetical protein	2	2	1	0	0	1	0	0	0	0	0	0	0	0	0	0
87	5221	hisFII	Imidazole glycerol phosphate synthase subunit HisF	2	3	1	0	0	1	0	0	0	0	0	0	0	0	0	1
88	9070	hisH	Imidazole glycerol phosphate synthase subunit hisH	2	3	1	0	0	1	0	0	0	0	0	0	0	0	0	1
89	2527		Possible oxidoreductase, GFO/Idh/MocA family protein	2	2	1	0	0	1	0	0	0	0	0	0	0	0	0	0
90	2528		Possible acylneuraminate cytidylyltransferase	2	2	1	0	0	1	0	0	0	0	0	0	0	0	0	0
91	2532		Possible hexapeptide transferase family protein	2	2	1	0	0	1	0	0	0	0	0	0	0	0	0	0
92	2525		Possible short-chain dehydrogenase family protein	2	2	1	0	0	1	0	0	0	0	0	0	0	0	0	0
93	2584		Transglutaminase-like protein	2	2	1	0	0	1	0	0	0	0	0	0	0	0	0	0
94	3149		Conserved hypothetical protein	2	2	1	0	0	0	1	0	0	0	0	0	0	0	0	0
95	3178		Conserved hypothetical protein	2	2	1	0	0	0	1	0	0	0	0	0	0	0	0	0
96	3257		Conserved hypothetical proteir	2	2	1	0	0	0	1	0	0	0	0	0	0	0	0	0
97	3328		Conserved hypothetical protein	2	2	1	0	0	0	1	0	0	0	0	0	0	0	0	0
98	7761		Conserved hypothetical protein	2	5	3	0	0	0	2	0	0	0	0	0	0	0	0	0
99	8133		Conserved hypothetical protein	2	3	1	0	0	0	2	0	0	0	0	0	0	0	0	0
100	3305		Possible flavoprotein	2	2	1	0	0	0	0	1	0	0	0	0	0	0	0	0
101	2586		Conserved hypothetical protein	2	2	1	0	0	0	0	0	1	0	0	0	0	0	0	0
102	2556		Conserved hypothetical protein	2	2	1	0	0	0	0	0	0	1	0	0	0	0	0	0
103	2558		Conserved hypothetical protein	2	2	1	0	0	0	0	0	0	1	0	0	0	0	0	0
104	2618		Conserved hypothetical protein	2	2	1	0	0	0	0	0	0	1	0	0	0	0	0	0
105	4370		Conserved hypothetical protein	2	2	1	0	0	0	0	0	0	1	0	0	0	0	0	0
106	2557		Sulfotransferase	2	2	1	0	0	0	0	0	0	1	0	0	0	0	0	0
107	2529		Conserved hypothetical protein	2	2	1	0	0	0	0	0	0	0	1	0	0	0	0	0
108	2541		Possible oxidoreductase	2	2	1	0	0	0	0	0	0	0	1	0	0	0	0	0
109	2522		Conserved hypothetical protein	2	2	1	0	0	0	0	0	0	0	0	1	0	0	0	0
110	2265		Conserved hypothetical membrane protein	2	3	1	0	0	0	0	0	0	0	0	2	0	0	0	0

						Synechococcus											Prochlorococcus		
						Subcluster 5.1A					Subcluster 5.1B				5.2	5.3	HL	LL	
Line No.	Cluster No. in cyanorak	Gene Name	Product	No. of *Synechococcus* strains per cluster	No. of sequ. per cluster (14 genomes)	BL107	CC9902	CC9605	WH8102	CC9311	WH7803	WH7805	RS9916	RS9917	WH5701	RCC307	MED4	SS120	MIT9313
111	8010		truncated cation efflux transporter (CDF family)	2	5	0	1	4	0	0	0	0	0	0	0	0	0	0	0
112	2093		Conserved hypothetical protein	2	5	0	1	0	4	0	0	0	0	0	0	0	0	0	0
113	2094		Conserved hypothetical protein	2	5	0	1	0	4	0	0	0	0	0	0	0	0	0	0
114	2480		Conserved hypothetical protein	2	9	0	1	0	8	0	0	0	0	0	0	0	0	0	0
115	2882		Conserved hypothetical protein	2	2	0	1	0	1	0	0	0	0	0	0	0	0	0	0
116	2916		Conserved hypothetical protein	2	2	0	1	0	1	0	0	0	0	0	0	0	0	0	0
117	5183		Conserved hypothetical protein	2	2	0	1	0	1	0	0	0	0	0	0	0	0	0	0
118	2881		Possible beta-glycosyltransferase, family 2	2	2	0	1	0	1	0	0	0	0	0	0	0	0	0	0
119	6458		Conserved hypothetical membrane protein	2	2	0	1	0	0	1	0	0	0	0	0	0	0	0	0
120	6498		Conserved hypothetical membrane protein	2	2	0	1	0	0	1	0	0	0	0	0	0	0	0	0
121	2336		Conserved hypothetical protein	2	3	0	1	0	0	0	1	0	0	0	0	0	0	0	1
122	2495		Conserved hypothetical protein	2	3	0	1	0	0	0	2	0	0	0	0	0	0	0	0
123	2496		Conserved hypothetical protein	2	3	0	1	0	0	0	2	0	0	0	0	0	0	0	0
124	2959		Conserved hypothetical protein	2	2	0	1	0	0	0	1	0	0	0	0	0	0	0	0
125	2961		Conserved hypothetical protein	2	2	0	1	0	0	0	1	0	0	0	0	0	0	0	0
126	2962		Conserved hypothetical protein	2	2	0	1	0	0	0	1	0	0	0	0	0	0	0	0
127	2967		Conserved hypothetical protein	2	2	0	1	0	0	0	1	0	0	0	0	0	0	0	0
128	5932		conserved hypothetical protein distanly related to glycosyltransferases	2	2	0	1	0	0	0	1	0	0	0	0	0	0	0	0
129	2966		conserved hypothetical protein distantly related to alpha-glycosyltransferases, family 4	2	2	0	1	0	0	0	1	0	0	0	0	0	0	0	0
130	5935		conserved hypothetical protein distantly related to glycosyltransferases, family 9	2	2	0	1	0	0	0	1	0	0	0	0	0	0	0	0
131	2965		Conserved hypothetical protein with tetratricopeptide repeat domains	2	2	0	1	0	0	0	1	0	0	0	0	0	0	0	0
132	2963		Possible beta-glycosyltransferase, family 41	2	2	0	1	0	0	0	1	0	0	0	0	0	0	0	0
133	2964		Possible beta-glycosyltransferase, family 41	2	2	0	1	0	0	0	1	0	0	0	0	0	0	0	0
134	2960		Possible Zn-dependent metalloprotease	2	3	0	1	0	0	0	1	0	0	0	0	0	0	0	1
135	3002		Possible multicopper oxidase	2	2	0	1	0	0	0	1	0	0	0	0	0	0	0	0
136	4695		Conserved hypothetical protein	2	2	0	1	0	0	0	0	0	1	0	0	0	0	0	0
137	2838		N-acetylneuraminate synthase	2	2	0	1	0	0	0	0	0	0	1	0	0	0	0	0
138	2337		Possible nucleoside-diphosphate sugar epimerase	2	3	0	1	0	0	0	0	0	0	1	0	0	0	0	1
139	2241		Conserved hypothetical protein	2	4	0	0	2	2	0	0	0	0	0	0	0	0	0	0
140	2885		Conserved hypothetical protein	2	2	0	0	1	1	0	0	0	0	0	0	0	0	0	0
141	2891		Conserved hypothetical protein	2	2	0	0	1	1	0	0	0	0	0	0	0	0	0	0
142	2894		Conserved hypothetical protein	2	2	0	0	1	1	0	0	0	0	0	0	0	0	0	0
143	2895		Conserved hypothetical protein	2	2	0	0	1	1	0	0	0	0	0	0	0	0	0	0
144	2896		Conserved hypothetical protein	2	2	0	0	1	1	0	0	0	0	0	0	0	0	0	0
145	2900		Conserved hypothetical protein	2	2	0	0	1	1	0	0	0	0	0	0	0	0	0	0
146	2902		Conserved hypothetical protein	2	2	0	0	1	1	0	0	0	0	0	0	0	0	0	0
147	2903		Conserved hypothetical protein	2	2	0	0	1	1	0	0	0	0	0	0	0	0	0	0
148	2904		Conserved hypothetical protein	2	2	0	0	1	1	0	0	0	0	0	0	0	0	0	0
149	2908		Conserved hypothetical protein	2	2	0	0	1	1	0	0	0	0	0	0	0	0	0	0
150	2912		Conserved hypothetical protein	2	2	0	0	1	1	0	0	0	0	0	0	0	0	0	0
151	2918		Conserved hypothetical protein	2	2	0	0	1	1	0	0	0	0	0	0	0	0	0	0
152	3055		Conserved hypothetical protein	2	3	0	0	2	1	0	0	0	0	0	0	0	0	0	0
153	5272		Conserved hypothetical protein	2	2	0	0	1	1	0	0	0	0	0	0	0	0	0	0
154	6262		Conserved hypothetical protein	2	2	0	0	1	1	0	0	0	0	0	0	0	0	0	0
155	6273		Conserved hypothetical protein	2	2	0	0	1	1	0	0	0	0	0	0	0	0	0	0
156	6425		Conserved hypothetical protein	2	2	0	0	1	1	0	0	0	0	0	0	0	0	0	0
157	2327		Conserved hypothetical protein	2	3	0	0	1	1	0	0	0	0	0	0	0	1	0	0
158	2097		Phage integrase family	2	5	0	0	3	2	0	0	0	0	0	0	0	0	0	0
159	2890		Possible alpha-glycosyltransferase, family 60	2	2	0	0	1	1	0	0	0	0	0	0	0	0	0	0
160	2343		Possible-TPR Domain containing protein	2	3	0	0	1	1	0	0	0	0	0	0	0	0	0	1
161	2911		Possible cytidine/deoxycytidine deaminase	2	2	0	0	1	1	0	0	0	0	0	0	0	0	0	0
162	6180		Conserved hypothetical membrane protein	2	2	0	0	1	0	1	0	0	0	0	0	0	0	0	0
163	6217		Conserved hypothetical membrane protein	2	2	0	0	1	0	1	0	0	0	0	0	0	0	0	0
164	6324		Conserved hypothetical membrane protein	2	2	0	0	1	0	1	0	0	0	0	0	0	0	0	0
165	6368		Conserved hypothetical membrane protein	2	2	0	0	1	0	1	0	0	0	0	0	0	0	0	0
166	6403		Conserved hypothetical membrane protein	2	2	0	0	1	0	1	0	0	0	0	0	0	0	0	0
167	2511		Conserved hypothetical protein	2	6	0	0	3	0	3	0	0	0	0	0	0	0	0	0
168	3068		Alpha/beta superfamily hydrolase	2	2	0	0	1	0	0	0	1	0	0	0	0	0	0	0
169	3053		Conserved hypothetical protein	2	2	0	0	1	0	0	0	1	0	0	0	0	0	0	0
170	3066		Conserved hypothetical protein	2	2	0	0	1	0	0	0	1	0	0	0	0	0	0	0
171	3067		Conserved hypothetical protein	2	2	0	0	1	0	0	0	1	0	0	0	0	0	0	0
172	7397		Conserved hypothetical protein	2	2	0	0	1	0	0	0	1	0	0	0	0	0	0	0
173	8986		Conserved hypothetical protein	2	2	0	0	1	0	0	0	1	0	0	0	0	0	0	0
174	8076		ABC-type multidrug transport system, ATPase and permease components	2	3	0	0	1	0	0	0	0	1	0	0	0	0	0	1
175	6117		Conserved hypothetical membrane protein	2	3	0	0	2	0	0	0	0	1	0	0	0	0	0	0
176	2382		Conserved hypothetical protein	2	3	0	0	2	0	0	0	0	1	0	0	0	0	0	0
177	2741		Conserved hypothetical protein	2	2	0	0	1	0	0	0	0	1	0	0	0	0	0	0
178	2780		Conserved hypothetical protein	2	2	0	0	1	0	0	0	0	1	0	0	0	0	0	0
179	2781		Conserved hypothetical protein	2	2	0	0	1	0	0	0	0	1	0	0	0	0	0	0
180	4413		Conserved hypothetical protein	2	2	0	0	1	0	0	0	0	1	0	0	0	0	0	0
181	4649		Conserved hypothetical protein	2	2	0	0	1	0	0	0	0	1	0	0	0	0	0	0
182	2405		Conserved hypothetical protein	2	3	0	0	2	0	0	0	0	1	0	0	0	0	0	0
183	2739		Glutathione S-transferase	2	2	0	0	1	0	0	0	0	1	0	0	0	0	0	0
184	2363		membrane-fusion protein	2	3	0	0	1	0	0	0	0	1	0	0	0	0	0	1
185	2760		Peptidase family M23	2	2	0	0	1	0	0	0	0	1	0	0	0	0	0	0
186	2748		Possible aminoglycoside phosphotransferase	2	2	0	0	1	0	0	0	0	1	0	0	0	0	0	0
187	2770		Possible molybdopterin synthase sulfurylase	2	2	0	0	1	0	0	0	0	1	0	0	0	0	0	0
188	2738		Sulfotransferase domain fused to a GAF domain	2	2	0	0	1	0	0	0	0	1	0	0	0	0	0	0
189	2821		Conserved hypothetical protein	2	2	0	0	1	0	0	0	0	0	1	0	0	0	0	0
190	2822		Conserved hypothetical protein	2	2	0	0	1	0	0	0	0	0	1	0	0	0	0	0
191	2863		Conserved hypothetical protein	2	2	0	0	1	0	0	0	0	0	1	0	0	0	0	0
192	4861		Conserved hypothetical protein	2	2	0	0	1	0	0	0	0	0	1	0	0	0	0	0
193	9115		Possible P-type ATPase transporter for copper	2	2	0	0	1	0	0	0	0	0	1	0	0	0	0	0
194	3070		Conserved hypothetical protein	2	2	0	0	1	0	0	0	0	0	0	1	0	0	0	0
195	2509		Monoamine oxidase	2	3	0	0	2	0	0	0	0	0	0	1	0	0	0	0
196	3069		Possible acetamidase/formamidas	2	2	0	0	1	0	0	0	0	0	0	1	0	0	0	0
197	2360		Conserved hypothetical membrane protein	2	3	0	0	1	0	0	0	0	0	0	0	1	0	0	1
198	2682		Conserved hypothetical protein	2	6	0	0	2	0	0	0	0	0	0	0	1	1	1	1
199	2929		Conserved hypothetical protein	2	2	0	0	1	0	0	0	0	0	0	0	1	0	0	0
200	2938		Conserved hypothetical protein	2	2	0	0	1	0	0	0	0	0	0	0	1	0	0	0
201	2354		Hexameric replicative helicase RepA-like domain-containing protein	2	3	0	0	1	0	0	0	0	0	0	0	1	0	0	1
202	5422		Conserved hypothetical protein	2	2	0	0	0	1	1	0	0	0	0	0	0	0	0	0
203	2917		Atypical alkaline phosphatase with phytase-like insertion	2	2	0	0	0	1	0	1	0	0	0	0	0	0	0	0
204	2888		Conserved hypothetical protein	2	2	0	0	0	1	0	1	0	0	0	0	0	0	0	0
205	2898		Conserved hypothetical protein	2	2	0	0	0	1	0	0	1	0	0	0	0	0	0	0
206	2910		Conserved hypothetical protein	2	2	0	0	0	1	0	0	1	0	0	0	0	0	0	0
207	1936		phage integrase family	2	6	0	0	0	4	0	0	1	0	0	0	0	0	0	1
208	2897		Possible beta-glycosyltransferase, family 41	2	2	0	0	0	1	0	0	1	0	0	0	0	0	0	0
209	2883		Possible glycosyltransferase	2	2	0	0	0	1	0	0	1	0	0	0	0	0	0	0
210	2749		Conserved hypothetical protein	2	2	0	0	0	1	0	0	0	1	0	0	0	0	0	0
211	2750		Conserved hypothetical protein	2	2	0	0	0	1	0	0	0	1	0	0	0	0	0	0
212	2766		Conserved hypothetical protein	2	2	0	0	0	1	0	0	0	1	0	0	0	0	0	0
213	2843		Conserved hypothetical protein	2	2	0	0	0	1	0	0	0	0	1	0	0	0	0	0
214	2345		Conserved hypothetical protein	2	3	0	0	0	1	0	0	0	0	1	0	0	0	0	1
215	6893		Conserved hypothetical protein	2	2	0	0	0	1	0	0	0	0	0	1	0	0	0	0
216	7193		Conserved hypothetical protein	2	2	0	0	0	1	0	0	0	0	0	1	0	0	0	0
217	1988		Possible beta-glycosyltransferase, family 2	2	4	0	0	0	2	0	0	0	0	0	2	0	0	0	0
218	3		Possible cyanate ABC transporter	2	4	0	0	0	1	0	0	0	0	0	2	0	1	0	0
219	2166		Possible cyanate ABC transporter, permease protein	2	4	0	0	0	1	0	0	0	0	0	2	0	1	0	0
220	2165		Possible cyanate ABC transporter, substrate binding protein	2	4	0	0	0	1	0	0	0	0	0	2	0	1	0	0
221	2919		Conserved hypothetical protein	2	2	0	0	0	1	0	0	0	0	0	0	1	0	0	0

Line No.	Cluster No. in cyanorak	Gene Name	Product	No. of *Synechococcus* strains per cluster	No. of sequ. per cluster (14 genomes)	Synechococcus Subcluster 5.1A					Synechococcus Subcluster 5.1B				Synechococcus 5.2	Synechococcus 5.3	Prochlorococcus HL	Prochlorococcus LL	
						BL107	CC9902	CC9605	WH8102	CC9311	WH7803	WH7805	RS9916	RS9917	WH5701	RCC307	MED4	SS120	MIT9313
222	6067		Conserved hypothetical membrane protein	2	2	0	0	0	0	1	1	0	0	0	0	0	0	0	0
223	8648		Conserved hypothetical protein	2	2	0	0	0	0	1	1	0	0	0	0	0	0	0	0
224	6040		Conserved hypothetical protein	2	5	0	0	0	0	4	1	0	0	0	0	0	0	0	0
225	6079		Possible transcriptional regulator	2	2	0	0	0	0	1	1	0	0	0	0	0	0	0	0
226	7348		Conserved hypothetical protein	2	2	0	0	0	0	1	0	1	0	0	0	0	0	0	0
227	7349		Conserved hypothetical protein	2	2	0	0	0	0	1	0	1	0	0	0	0	0	0	0
228	7383		Conserved hypothetical protein	2	2	0	0	0	0	1	0	1	0	0	0	0	0	0	0
229	7387		Conserved hypothetical protein	2	2	0	0	0	0	1	0	1	0	0	0	0	0	0	0
230	7479		Conserved hypothetical protein	2	2	0	0	0	0	1	0	1	0	0	0	0	0	0	0
231	7504		Conserved hypothetical protein	2	2	0	0	0	0	1	0	1	0	0	0	0	0	0	0
232	7506		Conserved hypothetical protein	2	2	0	0	0	0	1	0	1	0	0	0	0	0	0	0
233	7644		Conserved hypothetical protein	2	2	0	0	0	0	1	0	1	0	0	0	0	0	0	0
234	7703		Conserved hypothetical protein	2	2	0	0	0	0	1	0	1	0	0	0	0	0	0	0
235	7716		Conserved hypothetical protein	2	2	0	0	0	0	1	0	1	0	0	0	0	0	0	0
236	8210		Conserved hypothetical protein	2	2	0	0	0	0	1	0	1	0	0	0	0	0	0	0
237	8419		Conserved hypothetical protein	2	2	0	0	0	0	1	0	1	0	0	0	0	0	0	0
238	8440		Conserved hypothetical protein	2	2	0	0	0	0	1	0	1	0	0	0	0	0	0	0
239	7499		Possible urea transporter, UT family	2	2	0	0	0	0	1	0	1	0	0	0	0	0	0	0
240	7526		Response regulator receiver domain	2	2	0	0	0	0	1	0	1	0	0	0	0	0	0	0
241	7572		Sodium:solute symporter family	2	2	0	0	0	0	1	0	1	0	0	0	0	0	0	0
242	4518		Conserved hypothetical protein	2	2	0	0	0	0	1	0	0	1	0	0	0	0	0	0
243	4612		Conserved hypothetical protein	2	2	0	0	0	0	1	0	0	1	0	0	0	0	0	0
244	4620		Conserved hypothetical protein	2	2	0	0	0	0	1	0	0	1	0	0	0	0	0	0
245	4672		Conserved hypothetical protein	2	2	0	0	0	0	1	0	0	1	0	0	0	0	0	0
246	4688		Conserved hypothetical protein	2	2	0	0	0	0	1	0	0	1	0	0	0	0	0	0
247	4714		Conserved hypothetical protein	2	2	0	0	0	0	1	0	0	1	0	0	0	0	0	0
248	4751		Conserved hypothetical protein	2	2	0	0	0	0	1	0	0	1	0	0	0	0	0	0
249	4761		Conserved hypothetical protein	2	2	0	0	0	0	1	0	0	1	0	0	0	0	0	0
250	8127		Dehydrogenase (related to short-chain alcohol dehydrogenases)	2	2	0	0	0	0	1	0	0	1	0	0	0	0	0	0
251	4599		Possible FAD-dependent oxidoreductase	2	2	0	0	0	0	1	0	0	1	0	0	0	0	0	0
252	4454		Possible transpoter of the major facilitator superfamily	2	2	0	0	0	0	1	0	0	1	0	0	0	0	0	0
253	4853		Conserved hypothetical protein	2	2	0	0	0	0	1	0	0	0	1	0	0	0	0	0
254	4913		Conserved hypothetical protein	2	2	0	0	0	0	1	0	0	0	1	0	0	0	0	0
255	5087		Conserved hypothetical protein	2	2	0	0	0	0	1	0	0	0	1	0	0	0	0	0
256	4837		Possible permease of the major facilitator superfamily	2	2	0	0	0	0	1	0	0	0	1	0	0	0	0	0
257	6683		Conserved hypothetical membrane protein	2	2	0	0	0	0	1	0	0	0	0	1	0	0	0	0
258	7075		Acyl-coenzyme A synthetases/AMP-(fatty) acid ligases	2	2	0	0	0	0	1	0	0	0	0	1	0	0	0	0
259	6566		Aldo/keto reductase family	2	2	0	0	0	0	1	0	0	0	0	1	0	0	0	0
260	2690		Conserved hypothetical protein	2	3	0	0	0	0	1	0	0	0	0	1	0	0	0	1
261	6930		Conserved hypothetical protein	2	2	0	0	0	0	1	0	0	0	0	1	0	0	0	0
262	6939		Conserved hypothetical protein	2	2	0	0	0	0	1	0	0	0	0	1	0	0	0	0
263	6948		Conserved hypothetical protein	2	2	0	0	0	0	1	0	0	0	0	1	0	0	0	0
264	7319		Conserved hypothetical protein	2	2	0	0	0	0	1	0	0	0	0	1	0	0	0	0
265	6743		Oxidoreductase alpha (molybdopterin) subunit	2	2	0	0	0	0	1	0	0	0	0	1	0	0	0	0
266	6622		RNA polymerase ECF-type (group 3) sigma factor	2	2	0	0	0	0	1	0	0	0	0	1	0	0	0	0
267	2699		Transaldolase	2	3	0	0	0	0	1	0	0	0	0	1	0	0	0	1
268	8138		Aldehyde dehydrogenase family protein	2	3	0	0	0	0	2	0	0	0	0	0	1	0	0	0
269	5642		Conserved hypothetical protein	2	2	0	0	0	0	1	0	0	0	0	0	1	0	0	0
270	5831		Gluconolactonase	2	2	0	0	0	0	1	0	0	0	0	0	1	0	0	0
271	2994		Alcohol dehydrogenase, class IV	2	2	0	0	0	0	0	1	1	0	0	0	0	0	0	0
272	2974		Conserved hypothetical membrane protein	2	2	0	0	0	0	0	1	1	0	0	0	0	0	0	0
273	2980		Conserved hypothetical membrane protein	2	2	0	0	0	0	0	1	1	0	0	0	0	0	0	0
274	2986		Conserved hypothetical membrane protein	2	2	0	0	0	0	0	1	1	0	0	0	0	0	0	0
275	2996		Conserved hypothetical membrane protein	2	2	0	0	0	0	0	1	1	0	0	0	0	0	0	0
276	3000		Conserved hypothetical membrane protein	2	2	0	0	0	0	0	1	1	0	0	0	0	0	0	0
277	3019		Conserved hypothetical membrane protein	2	2	0	0	0	0	0	1	1	0	0	0	0	0	0	0
278	3030		Conserved hypothetical membrane protein	2	2	0	0	0	0	0	1	1	0	0	0	0	0	0	0
279	3038		Conserved hypothetical membrane protein	2	2	0	0	0	0	0	1	1	0	0	0	0	0	0	0
280	3044		Conserved hypothetical membrane protein	2	2	0	0	0	0	0	1	1	0	0	0	0	0	0	0
281	3045		Conserved hypothetical membrane protein	2	2	0	0	0	0	0	1	1	0	0	0	0	0	0	0
282	2501		Conserved hypothetical protein	2	3	0	0	0	0	0	1	2	0	0	0	0	0	0	0
283	2972		Conserved hypothetical protein	2	2	0	0	0	0	0	1	1	0	0	0	0	0	0	0
284	2977		Conserved hypothetical protein	2	2	0	0	0	0	0	1	1	0	0	0	0	0	0	0
285	2982		Conserved hypothetical protein	2	2	0	0	0	0	0	1	1	0	0	0	0	0	0	0
286	2983		Conserved hypothetical protein	2	2	0	0	0	0	0	1	1	0	0	0	0	0	0	0
287	2987		Conserved hypothetical protein	2	2	0	0	0	0	0	1	1	0	0	0	0	0	0	0
288	2988		Conserved hypothetical protein	2	2	0	0	0	0	0	1	1	0	0	0	0	0	0	0
289	3001		Conserved hypothetical protein	2	2	0	0	0	0	0	1	1	0	0	0	0	0	0	0
290	3004		Conserved hypothetical protein	2	2	0	0	0	0	0	1	1	0	0	0	0	0	0	0
291	3011		Conserved hypothetical protein	2	2	0	0	0	0	0	1	1	0	0	0	0	0	0	0
292	3021		Conserved hypothetical protein	2	2	0	0	0	0	0	1	1	0	0	0	0	0	0	0
293	3022		Conserved hypothetical protein	2	2	0	0	0	0	0	1	1	0	0	0	0	0	0	0
294	3027		Conserved hypothetical protein	2	2	0	0	0	0	0	1	1	0	0	0	0	0	0	0
295	3032		Conserved hypothetical protein	2	2	0	0	0	0	0	1	1	0	0	0	0	0	0	0
296	3046		Conserved hypothetical protein	2	2	0	0	0	0	0	1	1	0	0	0	0	0	0	0
297	5938		Conserved hypothetical protein	2	2	0	0	0	0	0	1	1	0	0	0	0	0	0	0
298	5983		Conserved hypothetical protein	2	2	0	0	0	0	0	1	1	0	0	0	0	0	0	0
299	7339		Conserved hypothetical protein	2	2	0	0	0	0	0	1	1	0	0	0	0	0	0	0
300	7342		Conserved hypothetical protein	2	2	0	0	0	0	0	1	1	0	0	0	0	0	0	0
301	7386		Conserved hypothetical protein	2	2	0	0	0	0	0	1	1	0	0	0	0	0	0	0
302	7388		Conserved hypothetical protein	2	2	0	0	0	0	0	1	1	0	0	0	0	0	0	0
303	7415		Conserved hypothetical protein	2	2	0	0	0	0	0	1	1	0	0	0	0	0	0	0
304	7417		Conserved hypothetical protein	2	2	0	0	0	0	0	1	1	0	0	0	0	0	0	0
305	7450		Conserved hypothetical protein	2	2	0	0	0	0	0	1	1	0	0	0	0	0	0	0
306	7453		Conserved hypothetical protein	2	2	0	0	0	0	0	1	1	0	0	0	0	0	0	0
307	7462		Conserved hypothetical protein	2	2	0	0	0	0	0	1	1	0	0	0	0	0	0	0
308	7508		Conserved hypothetical protein	2	2	0	0	0	0	0	1	1	0	0	0	0	0	0	0
309	7533		Conserved hypothetical protein	2	2	0	0	0	0	0	1	1	0	0	0	0	0	0	0
310	7608		Conserved hypothetical protein	2	2	0	0	0	0	0	1	1	0	0	0	0	0	0	0
311	7609		Conserved hypothetical protein	2	2	0	0	0	0	0	1	1	0	0	0	0	0	0	0
312	7623		Conserved hypothetical protein	2	2	0	0	0	0	0	1	1	0	0	0	0	0	0	0
313	7674		Conserved hypothetical protein	2	2	0	0	0	0	0	1	1	0	0	0	0	0	0	0
314	7677		Conserved hypothetical protein	2	2	0	0	0	0	0	1	1	0	0	0	0	0	0	0
315	7695		Conserved hypothetical protein	2	2	0	0	0	0	0	1	1	0	0	0	0	0	0	0
316	9002		Conserved hypothetical protein	2	2	0	0	0	0	0	1	1	0	0	0	0	0	0	0
317	2971		conserved hypothetical protein distantly related to glycosyltransferases, family 2	2	2	0	0	0	0	0	1	1	0	0	0	0	0	0	0
318	3047		Conserved hypothetical secreted protein	2	2	0	0	0	0	0	1	1	0	0	0	0	0	0	0
319	2969		Possible alpha-glycosyltransferase, family 4	2	2	0	0	0	0	0	1	1	0	0	0	0	0	0	0
320	2970		Possible alpha-glycosyltransferase, family 4	2	2	0	0	0	0	0	1	1	0	0	0	0	0	0	0
321	2995		Possible pectin lyase (parallel beta-helix repeat protein)	2	2	0	0	0	0	0	1	1	0	0	0	0	0	0	0
322	2997		Possible protein kinase-like proteir	2	2	0	0	0	0	0	1	1	0	0	0	0	0	0	0
323	2737		Conserved hypothetical membrane protein	2	2	0	0	0	0	0	1	0	1	0	0	0	0	0	0
324	2348		Conserved hypothetical protein	2	3	0	0	0	0	0	1	0	1	0	0	0	0	0	1
325	2724		Conserved hypothetical protein	2	2	0	0	0	0	0	1	0	1	0	0	0	0	0	0
326	2769		Conserved hypothetical protein	2	2	0	0	0	0	0	1	0	1	0	0	0	0	0	0
327	2771		Conserved hypothetical protein	2	2	0	0	0	0	0	1	0	1	0	0	0	0	0	0
328	2783		Conserved hypothetical protein	2	2	0	0	0	0	0	1	0	1	0	0	0	0	0	0
329	2785		Conserved hypothetical protein	2	2	0	0	0	0	0	1	0	1	0	0	0	0	0	0
330	4544		Conserved hypothetical protein	2	2	0	0	0	0	0	1	0	1	0	0	0	0	0	0
331	2786		Conserved membrane protein, Possible subunit of the Multisubunit Na+/H+ antiporter	2	2	0	0	0	0	0	1	0	1	0	0	0	0	0	0
332	2176		Dipeptidyl aminopeptidase/acylaminoacyl-peptidase	3	4	0	0	0	0	0	1	0	1	0	0	0	0	0	2

Line No.	Cluster No. in cyanorak	Gene Name	Product	No. of *Synechococcus* strains per cluster	No. of sequ. per cluster (14 genomes)	Synechococcus: Subcluster 5.1A: BL107	CC9902	CC9605	WH8102	CC9311	Subcluster 5.1B: WH7803	WH7805	RS9916	RS9917	5.2: WH5701	5.3: RCC307	Prochlorococcus: HL: MED4	SS120	LL: MIT9313
333	2817		Conserved hypothetical protein	2	2	0	0	0	0	0	1	0	0	1	0	0	0	0	0
334	2823		Conserved hypothetical membrane protein	2	2	0	0	0	0	0	1	0	0	1	0	0	0	0	0
335	2861		Conserved hypothetical membrane protein	2	2	0	0	0	0	0	1	0	0	1	0	0	0	0	0
336	2453		Conserved hypothetical protein	2	3	0	0	0	0	0	2	0	0	1	0	0	0	0	0
337	2857		Conserved hypothetical protein	2	2	0	0	0	0	0	1	0	0	1	0	0	0	0	0
338	2858		Conserved hypothetical protein	2	2	0	0	0	0	0	1	0	0	1	0	0	0	0	0
339	2862		Conserved hypothetical protein	2	2	0	0	0	0	0	1	0	0	1	0	0	0	0	0
340	5007		Conserved hypothetical protein	2	2	0	0	0	0	0	1	0	0	1	0	0	0	0	0
341	2815		Conserved hypothetical secreted protein	2	2	0	0	0	0	0	1	0	0	1	0	0	0	0	0
342	2426		MFS family transporter	2	3	0	0	0	0	0	1	0	0	2	0	0	0	0	0
343	2444		Possible ABC transporter, substrate binding protein	2	3	0	0	0	0	0	2	0	0	1	0	0	0	0	0
344	2868		Possible thiopurine S-methyltransferase	2	2	0	0	0	0	0	1	0	0	1	0	0	0	0	0
345	2870		Possible ATPase	2	2	0	0	0	0	0	1	0	0	1	0	0	0	0	0
346	2844		Two-component system response regulator	2	2	0	0	0	0	0	1	0	0	1	0	0	0	0	0
347	3015		Conserved hypothetical membrane protein	2	2	0	0	0	0	0	1	0	0	0	1	0	0	0	0
348	3017		Conserved hypothetical protein	2	2	0	0	0	0	0	1	0	0	0	1	0	0	0	0
349	2676		Peptide deformylase	2	3	0	0	0	0	0	1	0	0	0	1	0	0	0	1
350	3016		Spermidine synthase	2	2	0	0	0	0	0	1	0	0	0	1	0	0	0	0
351	2947		Conserved hypothetical protein	2	2	0	0	0	0	0	1	0	0	0	0	1	0	0	0
352	2949		Conserved hypothetical protein	2	2	0	0	0	0	0	1	0	0	0	0	1	0	0	0
353	2950		Conserved hypothetical protein	2	2	0	0	0	0	0	1	0	0	0	0	1	0	0	0
354	5712		Possible glyoxalase	2	2	0	0	0	0	0	1	0	0	0	0	1	0	0	0
355	2951		Possible cyanophycin synthetase	2	2	0	0	0	0	0	1	0	0	0	0	1	0	0	0
356	2490		TPR-repeat-containing protein	2	2	0	0	0	0	0	1	0	0	0	0	1	0	0	0
357	2948		TPR-repeat-containing protein	2	2	0	0	0	0	0	1	0	0	0	0	1	0	0	0
358	2372		Conserved hypothetical membrane protein	2	3	0	0	0	0	0	0	1	2	0	0	0	0	0	0
359	2763		Monoamine oxidase	2	2	0	0	0	0	0	0	1	1	0	0	0	0	0	0
360	2411		Carbohydrate-selective porin, OprB family	2	3	0	0	0	0	0	0	1	2	0	0	0	0	0	0
361	2765		Conserved hypothetical membrane protein	2	2	0	0	0	0	0	0	1	1	0	0	0	0	0	0
362	2381		Conserved hypothetical protein	2	3	0	0	0	0	0	0	2	1	0	0	0	0	0	0
363	2764		Conserved hypothetical protein	2	2	0	0	0	0	0	0	1	1	0	0	0	0	0	0
364	4452		Conserved hypothetical protein	2	2	0	0	0	0	0	0	1	1	0	0	0	0	0	0
365	4701		Conserved hypothetical protein	2	2	0	0	0	0	0	0	1	1	0	0	0	0	0	0
366	7451		Conserved hypothetical protein	2	2	0	0	0	0	0	0	1	1	0	0	0	0	0	0
367	2854		Conserved hypothetical protein	2	2	0	0	0	0	0	0	1	0	1	0	0	0	0	0
368	2853		ArsR Possible transcriptional regulator	2	2	0	0	0	0	0	0	1	0	1	0	0	0	0	0
369	2034		Conserved hypothetical protein	2	5	0	0	0	0	0	0	1	0	1	0	0	0	0	3
370	2850		Conserved hypothetical protein	2	2	0	0	0	0	0	0	1	0	1	0	0	0	0	0
371	7602		Conserved hypothetical protein	2	2	0	0	0	0	0	0	1	0	1	0	0	0	0	0
372	2855		RHOD domain-contaiining protein	2	2	0	0	0	0	0	0	1	0	1	0	0	0	0	0
373	6713		Possible ligand gated channel (GIC family)	2	2	0	0	0	0	0	0	1	0	0	1	0	0	0	0
374	3079		Conserved hypothetical protein	2	2	0	0	0	0	0	0	1	0	0	1	0	0	0	0
375	2954		Conserved hypothetical protein	2	2	0	0	0	0	0	0	1	0	0	0	1	0	0	0
376	2035		Site-specific recombinase	2	6	0	0	0	0	0	0	2	0	0	0	1	1	0	2
377	14		Conserved hypothetical protein	2	40	0	0	0	0	0	0	0	11	1	0	0	0	0	28
378	2398		Conserved hypothetical protein	2	3	0	0	0	0	0	0	0	1	2	0	0	0	0	0
379	2053		Conserved hypothetical protein	2	5	0	0	0	0	0	0	0	2	3	0	0	0	0	0
380	2174		Conserved hypothetical protein	2	4	0	0	0	0	0	0	0	1	2	0	0	0	0	1
381	2353		Conserved hypothetical protein	2	3	0	0	0	0	0	0	0	1	1	0	0	0	0	1
382	2726		Conserved hypothetical protein	2	2	0	0	0	0	0	0	0	1	1	0	0	0	0	0
383	2727		Conserved hypothetical protein	2	2	0	0	0	0	0	0	0	1	1	0	0	0	0	0
384	2728		Conserved hypothetical protein	2	2	0	0	0	0	0	0	0	1	1	0	0	0	0	0
385	2729		Conserved hypothetical protein	2	2	0	0	0	0	0	0	0	1	1	0	0	0	0	0
386	2730		Conserved hypothetical protein	2	2	0	0	0	0	0	0	0	1	1	0	0	0	0	0
387	2732		Conserved hypothetical protein	2	2	0	0	0	0	0	0	0	1	1	0	0	0	0	0
388	2733		Conserved hypothetical protein	2	2	0	0	0	0	0	0	0	1	1	0	0	0	0	0
389	2734		Conserved hypothetical protein	2	2	0	0	0	0	0	0	0	1	1	0	0	0	0	0
390	2761		Conserved hypothetical protein	2	2	0	0	0	0	0	0	0	1	1	0	0	0	0	0
391	2777		Conserved hypothetical protein	2	2	0	0	0	0	0	0	0	1	1	0	0	0	0	0
392	4332		Conserved hypothetical protein	2	2	0	0	0	0	0	0	0	1	1	0	0	0	0	0
393	4661		Conserved hypothetical protein	2	2	0	0	0	0	0	0	0	1	1	0	0	0	0	0
394	2725		Glycosyltransferase	2	2	0	0	0	0	0	0	0	1	1	0	0	0	0	0
395	1965		Outer membrane autotransporter barrel	2	5	0	0	0	0	0	0	0	4	1	0	0	0	0	0
396	2735		Possible phytanoyl-CoA dioxygenase	2	2	0	0	0	0	0	0	0	1	1	0	0	0	0	0
397	2742		Possible phytanoyl-CoA dioxygenase	2	2	0	0	0	0	0	0	0	1	1	0	0	0	0	0
398	1821		Conserved hypothetical protein	2	9	0	0	0	0	0	0	0	7	0	1	0	0	0	1
399	2338		Conserved hypothetical protein	2	3	0	0	0	0	0	0	0	1	0	1	0	0	0	1
400	2778		Conserved hypothetical membrane protein	2	2	0	0	0	0	0	0	0	1	0	0	1	0	0	0
401	2787		Conserved hypothetical protein	2	2	0	0	0	0	0	0	0	1	0	0	1	0	0	0
402	2731		Conserved hypothetical secreted protein	2	2	0	0	0	0	0	0	0	1	0	0	1	0	0	0
403	2736		Hypothetical protein	2	2	0	0	0	0	0	0	0	1	0	0	1	0	0	0
404	5613		Hypothetical protein	2	2	0	0	0	0	0	0	0	1	0	0	1	0	0	0
405	2782		Possible Zn peptidase	2	2	0	0	0	0	0	0	0	1	0	0	1	0	0	0
406	8023	mpeG	rod linker polypeptide (Lr), C-phycoerythrin II-associated	2	2	0	0	0	0	0	0	0	1	0	0	1	0	0	0
407	2814		Acyltransferase superfamily	2	2	0	0	0	0	0	0	0	0	1	1	0	0	0	0
408	2463		Antirestriction protein	2	3	0	0	0	0	0	0	0	0	1	2	0	0	0	0
409	191		Conserved hypothetical protein	2	14	0	0	0	0	0	0	0	0	13	1	0	0	0	0
410	1984		Conserved hypothetical protein	2	6	0	0	0	0	0	0	0	0	2	4	0	0	0	0
411	2077		Conserved hypothetical protein	2	3	0	0	0	0	0	0	0	0	1	2	0	0	0	0
412	2220		Conserved hypothetical protein	2	4	0	0	0	0	0	0	0	0	2	2	0	0	0	0
413	2235		Conserved hypothetical protein	2	4	0	0	0	0	0	0	0	0	3	1	0	0	0	0
414	2420		Conserved hypothetical protein	2	3	0	0	0	0	0	0	0	0	2	1	0	0	0	0
415	2421		Conserved hypothetical protein	2	3	0	0	0	0	0	0	0	0	2	1	0	0	0	0
416	2449		Conserved hypothetical protein	2	3	0	0	0	0	0	0	0	0	1	2	0	0	0	0
417	2791		Conserved hypothetical protein	2	2	0	0	0	0	0	0	0	0	1	1	0	0	0	0
418	2792		Conserved hypothetical protein	2	2	0	0	0	0	0	0	0	0	1	1	0	0	0	0
419	2793		Conserved hypothetical protein	2	2	0	0	0	0	0	0	0	0	1	1	0	0	0	0
420	2794		Conserved hypothetical protein	2	2	0	0	0	0	0	0	0	0	1	1	0	0	0	0
421	2795		Conserved hypothetical protein	2	2	0	0	0	0	0	0	0	0	1	1	0	0	0	0
422	2796		Conserved hypothetical protein	2	2	0	0	0	0	0	0	0	0	1	1	0	0	0	0
423	2797		Conserved hypothetical protein	2	2	0	0	0	0	0	0	0	0	1	1	0	0	0	0
424	2798		Conserved hypothetical protein	2	2	0	0	0	0	0	0	0	0	1	1	0	0	0	0
425	2799		Conserved hypothetical protein	2	2	0	0	0	0	0	0	0	0	1	1	0	0	0	0
426	2800		Conserved hypothetical protein	2	2	0	0	0	0	0	0	0	0	1	1	0	0	0	0
427	2801		Conserved hypothetical protein	2	2	0	0	0	0	0	0	0	0	1	1	0	0	0	0
428	2802		Conserved hypothetical protein	2	2	0	0	0	0	0	0	0	0	1	1	0	0	0	0
429	2807		Conserved hypothetical protein	2	2	0	0	0	0	0	0	0	0	1	1	0	0	0	0
430	2818		Conserved hypothetical protein	2	2	0	0	0	0	0	0	0	0	1	1	0	0	0	0
431	2830		Conserved hypothetical protein	2	2	0	0	0	0	0	0	0	0	1	1	0	0	0	0
432	2831		Conserved hypothetical protein	2	2	0	0	0	0	0	0	0	0	1	1	0	0	0	0
433	2832		Conserved hypothetical protein	2	2	0	0	0	0	0	0	0	0	1	1	0	0	0	0
434	2833		Conserved hypothetical protein	2	2	0	0	0	0	0	0	0	0	1	1	0	0	0	0
435	2836		Conserved hypothetical protein	2	2	0	0	0	0	0	0	0	0	1	1	0	0	0	0
436	2839		Conserved hypothetical protein	2	2	0	0	0	0	0	0	0	0	1	1	0	0	0	0
437	2840		Conserved hypothetical protein	2	2	0	0	0	0	0	0	0	0	1	1	0	0	0	0
438	2841		Conserved hypothetical protein	2	2	0	0	0	0	0	0	0	0	1	1	0	0	0	0
439	2842		Conserved hypothetical protein	2	2	0	0	0	0	0	0	0	0	1	1	0	0	0	0
440	2864		Conserved hypothetical protein	2	2	0	0	0	0	0	0	0	0	1	1	0	0	0	0
441	2866		Conserved hypothetical protein	2	2	0	0	0	0	0	0	0	0	1	1	0	0	0	0
442	2867		Conserved hypothetical protein	2	2	0	0	0	0	0	0	0	0	1	1	0	0	0	0
443	2873		Conserved hypothetical protein	2	3	0	0	0	0	0	0	0	0	2	1	0	0	0	0

						Synechococcus											Prochlorococcus		
						Subcluster 5.1A					Subcluster 5.1B				5.2	5.3	HL	LL	
Line No.	Cluster No. in cyanorak	Gene Name	Product	No. of *Synechococcus* strains per cluster	No. of sequ. per cluster (14 genomes)	BL107	CC9902	CC9605	WH8102	CC9311	WH7803	WH7805	RS9916	RS9917	WH5701	RCC307	MED4	SS120	MIT9313
444	2874		Conserved hypothetical protein	2	2	0	0	0	0	0	0	0	0	1	1	0	0	0	0
445	2875		Conserved hypothetical protein	2	2	0	0	0	0	0	0	0	0	1	1	0	0	0	0
446	2876		Conserved hypothetical protein	2	2	0	0	0	0	0	0	0	0	1	1	0	0	0	0
447	2878		Conserved hypothetical protein	2	2	0	0	0	0	0	0	0	0	1	1	0	0	0	0
448	2879		Conserved hypothetical protein	2	2	0	0	0	0	0	0	0	0	1	1	0	0	0	0
449	2880		Conserved hypothetical protein	2	2	0	0	0	0	0	0	0	0	1	1	0	0	0	0
450	9133		Conserved hypothetical protein	2	2	0	0	0	0	0	0	0	0	1	1	0	0	0	0
451	8856		D-lactate dehydrogenase	2	2	0	0	0	0	0	0	0	0	1	1	0	0	0	0
452	2221		FliA DNA-directed RNA polymerase specialized sigma subunit	2	4	0	0	0	0	0	0	0	0	2	2	0	0	0	0
453	2848		integral membrane signal transducer protein	2	2	0	0	0	0	0	0	0	0	1	1	0	0	0	0
454	1981		MgtA Cation transport ATPase	2	6	0	0	0	0	0	0	0	0	2	4	0	0	0	0
455	2465		NAD(FAD)-dependent dehydrogenase	2	3	0	0	0	0	0	0	0	0	2	1	0	0	0	0
456	12	cpcC	Phycobilisome linker polypeptide CpcC, phycocyanin-associated	2	5	0	0	0	0	0	0	0	0	2	3	0	0	0	0
457	2828	cpcD	Phycobilisome linker polypeptide CpcD, phycocyanin-associated	2	2	0	0	0	0	0	0	0	0	1	1	0	0	0	0
458	2860		Possible ABC transporter, membrane component	2	2	0	0	0	0	0	0	0	0	1	1	0	0	0	0
459	2466		Possible DNA/RNA helicase	2	3	0	0	0	0	0	0	0	0	1	2	0	0	0	0
460	192		possible transposase	2	14	0	0	0	0	0	0	0	0	13	1	0	0	0	0
461	2091		Restriction endonuclease	2	5	0	0	0	0	0	0	0	0	1	4	0	0	0	0
462	2851		Sterol desaturase	2	2	0	0	0	0	0	0	0	0	1	1	0	0	0	0
463	7995	sodA	superoxide dismutase [Mn]	2	2	0	0	0	0	0	0	0	0	1	1	0	0	0	0
464	2434		Transposase	2	3	0	0	0	0	0	0	0	0	1	2	0	0	0	0
465	2835		Transposase	2	2	0	0	0	0	0	0	0	0	1	1	0	0	0	0
466	2429		XerC Integrase	2	3	0	0	0	0	0	0	0	0	1	2	0	0	0	0
467	2804		XerC Integrase	2	2	0	0	0	0	0	0	0	0	1	1	0	0	0	0
468	2182		Acid phosphatase	2	4	0	0	0	0	0	0	0	0	1	0	1	0	0	2
469	2847		Conserved hypothetical protein	2	2	0	0	0	0	0	0	0	0	1	0	1	0	0	0
470	5031		Conserved hypothetical protein	2	2	0	0	0	0	0	0	0	0	1	0	1	0	0	0
471	2829		Kef-type K+ transport system, membrane component	2	2	0	0	0	0	0	0	0	0	1	0	1	0	0	0
472	2827		Conserved hypothetical protein related to phosphatidylserine/phosphatidylglycerophosphate	2	2	0	0	0	0	0	0	0	0	1	0	1	0	0	0
473	2845	clc4	Possible chloride channel	2	2	0	0	0	0	0	0	0	0	1	0	1	0	0	0
474	2926		alpha-glycosyltransferase, family 4	2	2	0	0	0	0	0	0	0	0	0	1	1	0	0	0
475	2936		Bacterioferritin	2	2	0	0	0	0	0	0	0	0	0	1	1	0	0	0
476	2922		beta-glycosyltransferase, family 2	2	2	0	0	0	0	0	0	0	0	0	1	1	0	0	0
477	2921		Conserved hypothetical membrane protein	2	2	0	0	0	0	0	0	0	0	0	1	1	0	0	0
478	2023		Conserved hypothetical protein	2	5	0	0	0	0	0	0	0	0	0	1	1	1	1	1
479	2934		Conserved hypothetical protein	2	2	0	0	0	0	0	0	0	0	0	1	1	0	0	0
480	2937		Conserved hypothetical protein	2	2	0	0	0	0	0	0	0	0	0	1	1	0	0	0
481	2941		Conserved hypothetical protein	2	2	0	0	0	0	0	0	0	0	0	1	1	0	0	0
482	2944		Conserved hypothetical protein	2	2	0	0	0	0	0	0	0	0	0	1	1	0	0	0
483	7223		Conserved hypothetical secreted protein	2	2	0	0	0	0	0	0	0	0	0	1	1	0	0	0
484	8105		Cysteine synthase	2	2	0	0	0	0	0	0	0	0	0	1	1	0	0	0
485	2942		Glycerol-3-phosphate dehydrogenase [NAD(P)+]	2	2	0	0	0	0	0	0	0	0	0	1	1	0	0	0
486	2945		glycoside hydrolase family 19, distantly related to chitinases	2	2	0	0	0	0	0	0	0	0	0	1	1	0	0	0
487	2483		hydrolase, HAD superfamily	2	3	0	0	0	0	0	0	0	0	0	2	1	0	0	0
488	2955		Iron-sulfur cluster binding protein	2	2	0	0	0	0	0	0	0	0	0	1	1	0	0	0
489	2923	btpA	Photosystem I assembly protein	2	2	0	0	0	0	0	0	0	0	0	1	1	0	0	0
490	2920		Possible alpha-glycosyltransferase, family 4	2	2	0	0	0	0	0	0	0	0	0	1	1	0	0	0
491	2935	cbiT	Precorrin-6Y C5,15-methyltransferase (Decarboxylating)	2	2	0	0	0	0	0	0	0	0	0	1	1	0	0	0
492	2943		Possible epimerase, PhzC/PhzF homolog	2	2	0	0	0	0	0	0	0	0	0	1	1	0	0	0
493	2927		Possible hydrolase (HAD superfamily)	2	2	0	0	0	0	0	0	0	0	0	1	1	0	0	0
494	2940		Possible periplasmic or secreted lipoprotein	2	2	0	0	0	0	0	0	0	0	0	1	1	0	0	0
495	9113	thrC	Threonine synthase	2	2	0	0	0	0	0	0	0	0	0	1	1	0	0	0
496	2306		ABC-type polysaccharide/polyol phosphate transport system, ATPase component	3	3	1	1	1	0	0	0	0	0	0	0	0	0	0	0
497	2297		Beta-lactamase class C and other penicillin-binding proteins	3	3	1	1	1	0	0	0	0	0	0	0	0	0	0	0
498	2121		Conserved hypothetical protein	3	7	2	1	1	0	0	0	0	0	0	0	0	1	1	1
499	2273		Conserved hypothetical protein	3	3	1	1	1	0	0	0	0	0	0	0	0	0	0	0
500	2275		Conserved hypothetical protein	3	3	1	1	1	0	0	0	0	0	0	0	0	0	0	0
501	2296		Conserved hypothetical protein	3	3	1	1	1	0	0	0	0	0	0	0	0	0	0	0
502	2304		Conserved hypothetical protein	3	3	1	1	1	0	0	0	0	0	0	0	0	0	0	0
503	2305		Conserved hypothetical protein	3	3	1	1	1	0	0	0	0	0	0	0	0	0	0	0
504	2310		Conserved hypothetical protein	3	3	1	1	1	0	0	0	0	0	0	0	0	0	0	0
505	2311		Conserved hypothetical protein	3	3	1	1	1	0	0	0	0	0	0	0	0	0	0	0
506	2313		Conserved hypothetical protein	3	3	1	1	1	0	0	0	0	0	0	0	0	0	0	0
507	2315		Conserved hypothetical protein	3	3	1	1	1	0	0	0	0	0	0	0	0	0	0	0
508	2317		Conserved hypothetical protein	3	3	1	1	1	0	0	0	0	0	0	0	0	0	0	0
509	2322		Conserved hypothetical protein	3	3	1	1	1	0	0	0	0	0	0	0	0	0	0	0
510	2519		Conserved hypothetical protein	3	4	1	1	1	0	0	0	0	0	0	0	0	0	0	1
511	2545		Conserved hypothetical protein	3	3	1	1	1	0	0	0	0	0	0	0	0	0	0	0
512	2578		Conserved hypothetical protein	3	3	1	1	1	0	0	0	0	0	0	0	0	0	0	0
513	2591		Conserved hypothetical protein	3	3	1	1	1	0	0	0	0	0	0	0	0	0	0	0
514	3240		Conserved hypothetical protein	3	3	1	1	1	0	0	0	0	0	0	0	0	0	0	0
515	2151		two-domain glycosyltransferase, family 2	3	3	1	1	1	0	0	0	0	0	0	0	0	0	0	0
516	2120		Conserved hypothetical protein	3	4	2	1	0	1	0	0	0	0	0	0	0	0	0	0
517	2272		Conserved hypothetical protein	3	3	1	1	0	1	0	0	0	0	0	0	0	0	0	0
518	3313		Conserved hypothetical protein	3	3	1	1	0	1	0	0	0	0	0	0	0	0	0	0
519	2316		Possible nuclease	3	3	1	1	0	1	0	0	0	0	0	0	0	0	0	0
520	2145	PTOX	Possible plastoquinol terminal oxidase	3	4	2	1	0	1	0	0	0	0	0	0	0	0	0	0
521	2314		Possible sulfotransferase protein	3	3	1	1	0	1	0	0	0	0	0	0	0	0	0	0
522	6501		Conserved hypothetical membrane protein	3	3	1	1	0	0	1	0	0	0	0	0	0	0	0	0
523	2293		Conserved hypothetical protein	3	4	1	1	0	0	1	0	0	0	0	0	0	0	1	0
524	2554		Conserved hypothetical protein	3	3	1	1	0	0	1	0	0	0	0	0	0	0	0	0
525	2565		Conserved hypothetical protein	3	3	1	1	0	0	1	0	0	0	0	0	0	0	0	0
526	2606		Conserved hypothetical protein	3	3	1	1	0	0	1	0	0	0	0	0	0	0	0	0
527	2610		Conserved hypothetical protein	3	3	1	1	0	0	1	0	0	0	0	0	0	0	0	0
528	2615		Conserved hypothetical protein	3	3	1	1	0	0	1	0	0	0	0	0	0	0	0	0
529	2619		Conserved hypothetical protein	3	3	1	1	0	0	1	0	0	0	0	0	0	0	0	0
530	3281		Conserved hypothetical protein	3	3	1	1	0	0	1	0	0	0	0	0	0	0	0	0
531	3324		Conserved hypothetical protein	3	3	1	1	0	0	1	0	0	0	0	0	0	0	0	0
532	2301	fda	Fructose-1,6-bisphosphate aldolase class I	3	4	1	1	0	0	1	0	0	0	0	0	0	0	1	0
533	2596		Photosystem II PsbY-like protein	3	3	1	1	0	0	1	0	0	0	0	0	0	0	0	0
534	8031	mpeF	rod linker polypeptide (Lr), C-phycoerythrin II-associated	3	3	1	1	0	0	1	0	0	0	0	0	0	0	0	0
535	3177		Conserved hypothetical protein	3	3	1	1	0	0	0	1	0	0	0	0	0	0	0	0
536	2276		Conserved hypothetical protein	3	3	1	1	0	0	0	0	0	1	0	0	0	0	0	0
537	2291		Conserved hypothetical protein	3	3	1	1	0	0	0	0	0	1	0	0	0	0	0	0
538	2536		Conserved hypothetical protein	3	3	1	1	0	0	0	0	0	0	1	0	0	0	0	0
539	2286		Pterin-4a-carbinolamine dehydratase	3	3	1	1	0	0	0	0	0	0	0	1	0	0	0	0
540	2320		Conserved hypothetical protein	3	3	1	0	1	1	0	0	0	0	0	0	0	0	0	0
541	2130		MoxR-like ATPase	3	4	1	0	1	2	0	0	0	0	0	0	0	0	0	0
542	2000		Superfamily I DNA/RNA helicase	3	5	1	0	2	0	0	0	1	0	0	0	0	0	1	0
543	2267		Possible nucleotide sugar epimerase	3	3	1	0	0	1	0	0	0	1	0	0	0	0	0	0
544	2266		Possible sugar-phosphate nucleotide transferase	3	3	1	0	0	1	0	0	0	1	0	0	0	0	0	0
545	3133		Possible sulfotransferase	3	3	1	0	0	1	0	0	0	0	0	1	0	0	0	0
546	2300		Sodium/glutamate symporter	3	3	1	0	0	1	0	0	0	0	0	1	0	0	0	0
547	2289		Guanosine polyphosphate pyrophosphohydrolase/synthetase	3	3	1	0	0	0	0	0	1	1	0	0	0	0	0	0
548	3338		Conserved hypothetical protein	3	3	1	0	0	0	0	0	0	1	0	1	0	0	0	0
549	2287		Metallophosphoesterase	3	3	1	0	0	0	0	0	0	1	0	1	0	0	0	0
550	2288		Methylglyoxal synthase	3	3	1	0	0	0	0	0	0	1	0	1	0	0	0	0
551	2240		Conserved hypothetical protein	3	4	0	1	2	1	0	0	0	0	0	0	0	0	0	0
552	2510		Conserved hypothetical protein	3	4	0	1	2	1	0	0	0	0	0	0	0	0	0	0
553	2907		Conserved hypothetical protein	3	3	0	1	1	1	0	0	0	0	0	0	0	0	0	0

						Synechococcus											Prochlorococcus		
						Subcluster 5.1A					Subcluster 5.1B				5.2	5.3	HL	LL	
Line No.	Cluster No. in cyanorak	Gene Name	Product	No. of *Synechococcus* strains per cluster	No. of sequ. per cluster (14 genomes)	BL107	CC9902	CC9605	WH8102	CC9311	WH7803	WH7805	RS9916	RS9917	WH5701	RCC307	MED4	SS120	MIT9313
554	2238		Possible phage integrase	3	4	0	1	2	1	0	0	0	0	0	0	0	0	0	0
555	2410		Conserved hypothetical protein	3	3	0	1	1	0	0	0	0	1	0	0	0	0	0	0
556	2236		Possible alpha-glycosyltransferase, family 4	3	4	0	1	0	1	0	0	2	0	0	0	0	0	0	0
557	2788		Conserved hypothetical membrane protein	3	3	0	1	0	0	1	0	0	1	0	0	0	0	0	0
558	2373		Conserved hypothetical protein with TPR repeat, possibly involved in cell envelope biogenesis	3	3	0	1	0	0	0	1	0	1	0	0	0	0	0	0
559	2349		Conserved hypothetical protein	3	4	0	0	1	1	0	0	0	0	1	0	0	0	0	1
560	2481		Conserved hypothetical protein	3	3	0	0	1	1	0	0	1	0	0	0	0	0	0	0
561	2939		Conserved hypothetical protein	3	3	0	0	1	1	0	0	0	0	0	0	1	0	0	0
562	3060		Conserved hypothetical protein	3	3	0	0	1	1	0	0	1	0	0	0	0	0	0	0
563	3642		Conserved hypothetical protein	3	4	0	0	1	1	0	0	0	1	0	0	0	0	0	1
564	4722		Conserved hypothetical protein	3	3	0	0	1	1	0	0	0	1	0	0	0	0	0	0
565	9027		Conserved hypothetical protein	3	3	0	0	1	1	0	0	0	1	0	0	0	0	0	0
566	2167		Conserved hypothetical protein	3	4	0	0	1	1	0	0	1	0	0	0	0	1	0	0
567	2402		Conserved hypothetical protein	3	3	0	0	1	1	0	0	0	1	0	0	0	0	0	0
568	2357		Conserved hypothetical membrane protein	3	4	0	0	1	0	1	0	0	0	0	1	0	0	0	1
569	2775		Conserved hypothetical protein	3	3	0	0	1	0	1	0	0	1	0	0	0	0	0	0
570	3947		Conserved hypothetical protein	3	4	0	0	1	0	1	0	0	1	0	0	0	0	1	0
571	7723		Conserved hypothetical protein	3	3	0	0	1	0	1	0	1	0	0	0	0	0	0	0
572	2487		Conserved hypothetical membrane protein	3	3	0	0	1	0	0	1	0	0	0	0	1	0	0	0
573	4449		Conserved hypothetical protein	3	3	0	0	1	0	0	0	0	1	0	0	1	0	0	0
574	5906		Conserved hypothetical protein	3	3	0	0	1	0	0	0	0	0	0	1	1	0	0	0
575	2028		beta-glycosyltransferase, family 2	3	5	0	0	1	0	0	0	0	0	0	1	1	1	0	1
576	2375		Possible ABC transporter, ATP binding and membrane components	3	3	0	0	0	1	0	1	0	1	0	0	0	0	0	0
577	2237		Conserved hypothetical protein	3	4	0	0	0	1	0	1	0	0	0	2	0	0	0	0
578	2482		Conserved hypothetical protein	3	3	0	0	0	1	0	1	0	0	0	1	0	0	0	0
579	2394		Conserved hypothetical protein	3	3	0	0	0	1	0	0	1	1	0	0	0	0	0	0
580	2260		Conserved hypothetical protein	3	4	0	0	0	1	0	0	1	0	0	2	0	0	0	0
581	2079		Possible ribosomal protein S6 modification protein	3	5	0	0	0	1	0	0	3	0	1	0	0	0	0	0
582	2184		Conserved hypothetical protein	3	4	0	0	0	1	0	0	0	1	1	0	0	0	1	0
583	1826		Conserved hypothetical protein with hemolysin-type calcium-binding regions	3	8	0	0	0	1	0	0	0	1	0	0	6	0	0	0
584	2228		Conserved hypothetical protein	3	4	0	0	0	1	0	0	0	0	1	2	0	0	0	0
585	2432		Conserved hypothetical protein	3	3	0	0	0	1	0	0	0	0	1	1	0	0	0	0
586	2433		Conserved hypothetical protein	3	3	0	0	0	1	0	0	0	0	1	1	0	0	0	0
587	2435		Conserved hypothetical protein	3	3	0	0	0	1	0	0	0	0	1	1	0	0	0	0
588	2436		Conserved hypothetical protein	3	3	0	0	0	1	0	0	0	0	1	1	0	0	0	0
589	2439		Conserved hypothetical protein	3	3	0	0	0	1	0	0	0	0	1	1	0	0	0	0
590	2440		Conserved hypothetical protein	3	3	0	0	0	1	0	0	0	0	1	1	0	0	0	0
591	2036		Possible nuclease	3	5	0	0	0	1	0	0	0	0	1	1	0	0	0	2
592	2826	chrA1	chromate transporter, CHR family	3	6	0	0	0	1	0	0	0	0	2	0	2	1	0	0
593	2478		UDP-N-acetylmuramyl pentapeptidephosphotransferase	3	3	0	0	0	1	0	0	0	0	0	1	1	0	0	0
594	3008		Conserved hypothetical membrane protein	3	3	0	0	0	0	1	1	1	0	0	0	0	0	0	0
595	2981		Conserved hypothetical protein	3	3	0	0	0	0	1	1	1	0	0	0	0	0	0	0
596	2984		Conserved hypothetical protein	3	3	0	0	0	0	1	1	1	0	0	0	0	0	0	0
597	2993		Conserved hypothetical protein	3	3	0	0	0	0	1	1	1	0	0	0	0	0	0	0
598	3007		Conserved hypothetical protein	3	3	0	0	0	0	1	1	1	0	0	0	0	0	0	0
599	3020		Conserved hypothetical protein	3	3	0	0	0	0	1	1	1	0	0	0	0	0	0	0
600	7965		Conserved hypothetical protein	3	3	0	0	0	0	1	1	1	0	0	0	0	0	0	0
601	3028		Conserved hypothetical secreted protein	3	3	0	0	0	0	1	1	1	0	0	0	0	0	0	0
602	2503		Possible ABC transporter, branched chain amino acid binding protein	3	5	0	0	0	0	2	1	2	0	0	0	0	0	0	0
603	2968		Possible alpha-glycosyltransferase, family 4	3	3	0	0	0	0	1	1	1	0	0	0	0	0	0	0
604	3012		Possible esterase related to lysophospholipase L1	3	3	0	0	0	0	1	1	1	0	0	0	0	0	0	0
605	2973		Sugar kinase, ribokinase family	3	3	0	0	0	0	1	1	1	0	0	0	0	0	0	0
606	2805		Conserved hypothetical protein	3	3	0	0	0	0	1	1	0	0	1	0	0	0	0	0
607	2856		Conserved hypothetical protein	3	3	0	0	0	0	1	1	0	0	1	0	0	0	0	0
608	2859		Conserved hypothetical protein related to glyoxalase/bleomycin resistance protein	3	3	0	0	0	0	1	1	0	0	1	0	0	0	0	0
609	2359		Possible transporter, MFS family	3	4	0	0	0	0	1	1	0	0	1	0	0	0	0	1
610	2933		Possible multicopper oxidase	3	3	0	0	0	0	1	1	0	0	0	0	1	0	0	0
611	2755		Conserved hpothetical protein	3	3	0	0	0	0	1	0	1	1	0	0	0	0	0	0
612	2767		Conserved hypothetical protein	3	3	0	0	0	0	1	0	1	1	0	0	0	0	0	0
613	2768		Conserved hypothetical protein	3	3	0	0	0	0	1	0	1	1	0	0	0	0	0	0
614	2747		PBPb, Bacterial periplasmic transport system protein	3	3	0	0	0	0	1	0	1	1	0	0	0	0	0	0
615	2813		Conserved hypothetical protein	3	3	0	0	0	0	1	0	1	0	1	0	0	0	0	0
616	3086		Conserved hypothetical protein	3	3	0	0	0	0	1	0	1	0	0	1	0	0	0	0
617	9124		Cation or drug efflux system protein	3	3	0	0	0	0	1	0	1	0	0	0	1	0	0	0
618	2946		Conserved hypothetical membrane protein	3	3	0	0	0	0	1	0	1	0	0	0	1	0	0	0
619	2930		Possible carboxyvinyl-carboxyphosphonate phosphorylmutase	3	3	0	0	0	0	1	0	1	0	0	0	1	0	0	0
620	2953		Possible secreted oxidoreductase	3	3	0	0	0	0	1	0	1	0	0	0	1	0	0	0
621	2789		Response regulator	3	3	0	0	0	0	1	0	0	1	1	0	0	0	0	0
622	2773		Conserved hypothetical protein	3	3	0	0	0	0	1	0	0	1	1	0	0	0	0	0
623	2759		LysR Transcriptional regulator	3	5	0	0	0	0	1	0	0	2	2	0	0	0	0	0
624	2790		two-component hybrid sensor histidine kinase	3	3	0	0	0	0	1	0	0	1	1	0	0	0	0	0
625	2347		Curli production assembly/transport component CsgG subfamily protein	3	4	0	0	0	0	1	0	0	1	0	1	0	0	0	1
626	8030		Two-component system response regulator	3	3	0	0	0	0	1	0	0	1	0	0	1	0	0	0
627	9107		Two-component system sensor histidine kinase	3	3	0	0	0	0	1	0	0	1	0	0	1	0	0	0
628	2361		Conserved hypothetical protein	3	4	0	0	0	0	1	0	0	0	1	1	0	0	0	1
629	2464	feoB	protein of the ferrous iron uptake family	3	4	0	0	0	0	1	0	0	0	2	1	0	0	0	0
630	2824		Uncharacterized protein involved in exopolysaccharide biosynthesis	3	3	0	0	0	0	1	0	0	0	1	1	0	0	0	0
631	2024	sbtA	Possible sodium-dependent bicarbonate transporter	3	6	0	0	0	0	1	0	0	0	0	1	1	1	1	1
632	2416		Conserved hypothetical protein	3	3	0	0	0	0	0	1	1	1	0	0	0	0	0	0
633	2414		Possible epimerase, PhzC/PhzF homolog	3	3	0	0	0	0	0	1	1	1	0	0	0	0	0	0
634	2451		Conserved hypothetical membrane protein	3	3	0	0	0	0	0	1	1	0	1	0	0	0	0	0
635	2438		Conserved hypothetical protein	3	3	0	0	0	0	0	1	1	0	1	0	0	0	0	0
636	2460		Conserved hypothetical protein	3	3	0	0	0	0	0	1	1	0	1	0	0	0	0	0
637	2461		Conserved hypothetical protein	3	3	0	0	0	0	0	1	1	0	1	0	0	0	0	0
638	2469		Conserved hypothetical secreted protein	3	3	0	0	0	0	0	1	1	0	1	0	0	0	0	0
639	2497		ABC efflux transporter, membrane component	3	3	0	0	0	0	0	1	1	0	0	1	0	0	0	0
640	2172		ABC-type polysaccharide/polyol phosphate transport system, ATPase component	3	4	0	0	0	0	0	1	1	0	0	1	0	0	0	1
641	2264		Conserved hypothetical membrane protein	3	4	0	0	0	0	0	1	1	0	0	2	0	0	0	0
642	2356		Conserved hypothetical membrane protein	3	6	0	0	0	0	0	1	1	0	0	1	0	0	1	2
643	2504		Hydrogenase/urease accessory protein	3	3	0	0	0	0	0	1	1	0	0	1	0	0	0	0
644	2491		Conserved hypothetical membrane protein	3	3	0	0	0	0	0	1	1	0	0	0	1	0	0	0
645	2485		Conserved hypothetical protein	3	3	0	0	0	0	0	1	1	0	0	0	1	0	0	0
646	2492		Cupin, RmlC-type superfamily protein	3	3	0	0	0	0	0	1	1	0	0	0	1	0	0	0
647	2484		DnaJ domain containing protein	3	3	0	0	0	0	0	1	1	0	0	0	1	0	0	0
648	2045		Two-component system response regulator	3	5	0	0	0	0	0	1	1	0	0	0	2	0	0	1
649	2384		Adenylate cyclase	3	3	0	0	0	0	0	1	0	1	1	0	0	0	0	0
650	2397		Conserved hypothetical membrane protein	3	3	0	0	0	0	0	1	0	1	1	0	0	0	0	0
651	2188		Conserved hypothetical protein	3	4	0	0	0	0	0	1	0	2	1	0	0	0	0	0
652	2383		Conserved hypothetical protein	3	3	0	0	0	0	0	1	0	1	1	0	0	0	0	0
653	2388		Conserved hypothetical protein	3	3	0	0	0	0	0	1	0	1	1	0	0	0	0	0
654	2396		Possible ABC transporter, substrate binding component	3	3	0	0	0	0	0	1	0	1	1	0	0	0	0	0
655	2442		Ca2+/H+ antiporter	3	3	0	0	0	0	0	1	0	0	1	1	0	0	0	0
656	2470		Conserved hypothetical protein	3	3	0	0	0	0	0	1	0	0	1	0	1	0	0	0
657	9119		sulfate ion transporter	3	4	0	0	0	0	0	1	0	0	0	2	1	0	0	0
658	2418		Conserved hypothetical protein	3	3	0	0	0	0	0	0	1	1	1	0	0	0	0	0
659	1886		Arylsulfatase A and related enzymes	3	7	0	0	0	0	0	0	2	2	0	2	0	0	0	1
660	1952		Conserved hypothetical protein	3	6	0	0	0	0	0	0	1	1	0	3	0	0	0	1
661	2386		Conserved hypothetical protein	3	3	0	0	0	0	0	0	1	1	0	1	0	0	0	0
662	2189		HAMP domain-containing protein	3	4	0	0	0	0	0	0	2	1	0	1	0	0	0	0
663	2037		Possible Na+-dependent transporter	3	5	0	0	0	0	0	0	1	1	0	2	0	0	0	1
664	2387		Possible Serine/Threonine kinase	3	3	0	0	0	0	0	0	1	1	0	1	0	0	0	0

Line No.	Cluster No. in cyanorak	Gene Name	Product	No. of *Synechococcus* strains per cluster	No. of sequ. per cluster (14 genomes)	Synechococcus Subcluster 5.1A BL107	CC9902	CC9605	WH8102	CC9311	Subcluster 5.1B WH7803	WH7805	RS9916	RS9917	5.2 WH5701	5.3 RCC307	Prochlorococcus HL MED4	SS120	LL MIT9313
665	2412		Nucleoside deaminase	3	3	0	0	0	0	0	0	1	1	0	0	1	0	0	0
666	2371		Possible phosphoribosyltransferase	3	3	0	0	0	0	0	0	1	1	0	0	1	0	0	0
667	2467		Nucleotidyltransferase domain, NTP_transf_2 superfamily	3	3	0	0	0	0	0	0	1	0	1	1	0	0	0	0
668	2232		Conserved hypothetical protein	3	4	0	0	0	0	0	0	1	0	1	2	0	0	0	0
669	2489		Conserved hypothetical secreted protein	3	3	0	0	0	0	0	0	1	0	0	1	1	0	0	0
670	2486		Conserved hypothetical membrane protein	3	3	0	0	0	0	0	0	1	0	0	1	1	0	0	0
671	2180		Possible mechanosensitive ion channel	3	4	0	0	0	0	0	0	0	1	1	1	0	0	0	1
672	8021	mscS3	small mechanosensitive ion channel, MscS family	3	4	0	0	0	0	0	0	0	1	1	1	0	0	0	1
673	2377		Conserved hypothetical secreted protein	3	3	0	0	0	0	0	0	0	1	1	0	1	0	0	0
674	2380		Conserved hypothetical protein	3	3	0	0	0	0	0	0	0	1	0	1	1	0	0	0
675	2417		Conserved hypothetical protein	3	3	0	0	0	0	0	0	0	1	0	1	1	0	0	0
676	2925		Peroxiredoxin	3	3	0	0	0	0	0	0	0	0	1	1	1	0	0	0
677	1903		beta-glycosyltransferase, family 2	4	4	1	1	1	1	0	0	0	0	0	0	0	0	0	0
678	2138		Conserved hypothetical membrane protein	4	4	1	1	1	1	0	0	0	0	0	0	0	0	0	0
679	1906		Conserved hypothetical protein	4	6	1	2	2	1	0	0	0	0	0	0	0	0	0	0
680	1924		Conserved hypothetical protein	4	6	1	1	1	1	0	0	0	0	0	0	0	1	1	0
681	2008		Conserved hypothetical protein	4	5	1	1	1	2	0	0	0	0	0	0	0	0	0	0
682	2010		Conserved hypothetical protein	4	5	1	1	1	1	0	0	0	0	0	0	0	1	0	0
683	2020		Conserved hypothetical protein	4	5	1	1	1	2	0	0	0	0	0	0	0	0	0	0
684	2101		Conserved hypothetical protein	4	4	1	1	1	1	0	0	0	0	0	0	0	0	0	0
685	2107		Conserved hypothetical protein	4	4	1	1	1	1	0	0	0	0	0	0	0	0	0	0
686	2111		Conserved hypothetical protein	4	4	1	1	1	1	0	0	0	0	0	0	0	0	0	0
687	2118		Conserved hypothetical protein	4	4	1	1	1	1	0	0	0	0	0	0	0	0	0	0
688	2126		Conserved hypothetical protein	4	4	1	1	1	1	0	0	0	0	0	0	0	0	0	0
689	2134		Conserved hypothetical protein	4	4	1	1	1	1	0	0	0	0	0	0	0	0	0	0
690	2137		Conserved hypothetical protein	4	4	1	1	1	1	0	0	0	0	0	0	0	0	0	0
691	2140		Conserved hypothetical protein	4	4	1	1	1	1	0	0	0	0	0	0	0	0	0	0
692	2143		Conserved hypothetical protein	4	4	1	1	1	1	0	0	0	0	0	0	0	0	0	0
693	2148		Conserved hypothetical protein	4	4	1	1	1	1	0	0	0	0	0	0	0	0	0	0
694	2149		Conserved hypothetical protein	4	4	1	1	1	1	0	0	0	0	0	0	0	0	0	0
695	2156		Conserved hypothetical protein	4	4	1	1	1	1	0	0	0	0	0	0	0	0	0	0
696	2160		Conserved hypothetical protein	4	4	1	1	1	1	0	0	0	0	0	0	0	0	0	0
697	2161		Conserved hypothetical protein	4	4	1	1	1	1	0	0	0	0	0	0	0	0	0	0
698	2162		Conserved hypothetical protein	4	4	1	1	1	1	0	0	0	0	0	0	0	0	0	0
699	2283		Conserved hypothetical protein	4	4	1	1	1	1	0	0	0	0	0	0	0	0	0	0
700	2298		Conserved hypothetical protein	4	4	1	1	1	1	0	0	0	0	0	0	0	0	0	0
701	2307		Conserved hypothetical protein	4	4	1	1	1	1	0	0	0	0	0	0	0	0	0	0
702	3225		Conserved hypothetical protein	4	4	1	1	1	1	0	0	0	0	0	0	0	0	0	0
703	9077		Conserved hypothetical protein	4	4	1	1	1	1	0	0	0	0	0	0	0	0	0	0
704	2153		Possible ABC polysaccharide efflux transporter, membrane component	4	4	1	1	1	1	0	0	0	0	0	0	0	0	0	0
705	2157		Possible acyl carrier protein	4	4	1	1	1	1	0	0	0	0	0	0	0	0	0	0
706	2159		Possible acylneuraminate cytidylyltransferase	4	4	1	1	1	1	0	0	0	0	0	0	0	0	0	0
707	2163		Possible integrase/recombinase	4	4	1	1	1	1	0	0	0	0	0	0	0	0	0	0
708	2102		Possible MTA/SAH nucleosidase	4	4	1	1	1	1	0	0	0	0	0	0	0	0	0	0
709	9017		Possible N-acetylneuraminic acid synthetase	4	4	1	1	1	1	0	0	0	0	0	0	0	0	0	0
710	2152		Possible polysaccharide efflux protein	4	4	1	1	1	1	0	0	0	0	0	0	0	0	0	0
711	2158		Possible polysaccharide export protein	4	4	1	1	1	1	0	0	0	0	0	0	0	0	0	0
712	1929		similar to capsule polysaccharide protein kpsS	4	6	2	1	2	1	0	0	0	0	0	0	0	0	0	0
713	2110		Conserved hypothetical protein	4	5	1	1	1	0	1	0	0	0	0	0	0	0	0	1
714	2280		Conserved hypothetical protein	4	4	1	1	1	0	1	0	0	0	0	0	0	0	0	0
715	8099		Ferredoxin	4	4	1	1	1	0	1	0	0	0	0	0	0	0	0	0
716	1833	isiB	Flavodoxin	4	8	1	1	2	0	1	0	0	0	0	0	0	1	1	1
717	9095	isiA	Iron stress-induced chlorophyll-binding protein (IsiA)	4	4	1	1	1	0	1	0	0	0	0	0	0	0	0	0
718	9073	cpcG3	phycobilisome rod-core linker polypeptide	4	4	1	1	1	0	1	0	0	0	0	0	0	0	0	0
719	2271		Possible hydrolases or acyltransferases (alpha/beta hydrolase superfamily)	4	4	1	1	1	0	1	0	0	0	0	0	0	0	0	0
720	1904	trxB	Thioredoxin-disulfide reductase	4	7	1	1	1	0	1	0	0	0	0	0	0	1	1	1
721	2106		Transcriptional regulator, AraC family	4	4	1	1	1	0	0	0	1	0	0	0	0	0	0	0
722	2004		Conserved hypothetical protein	4	5	1	1	1	0	0	0	0	1	0	0	0	0	0	1
723	2108		Conserved hypothetical protein	4	4	1	1	1	0	0	0	0	1	0	0	0	0	0	0
724	2546		Possible transcriptional regulator, Crp/Fnr family	4	5	1	1	1	0	0	0	0	0	1	0	0	0	0	1
725	1920		Conserved hypothetical protein	4	6	1	1	1	0	0	0	0	0	0	0	1	1	0	1
726	1839		Conserved hypothetical membrane protein	4	8	1	1	1	0	0	0	0	0	0	0	1	2	1	1
727	2144		Conserved hypothetical protein	4	4	1	1	0	1	0	0	0	1	0	0	0	0	0	0
728	2309		Possible gluconolactonase	4	4	1	1	0	0	1	0	0	0	0	0	1	0	0	0
729	2277		Conserved hypothetical membrane protein	4	4	1	1	0	0	1	0	1	0	0	0	0	0	0	0
730	2308		Conserved hypothetical protein	4	4	1	1	0	0	1	0	0	1	0	0	0	0	0	0
731	2319		Conserved hypothetical protein	4	5	1	1	0	0	1	0	0	2	0	0	0	0	0	0
732	2555		Conserved hypothetical protein	4	5	2	1	0	0	1	0	0	1	0	0	0	0	0	0
733	1990		Conserved hypothetical protein	4	6	1	1	0	0	1	0	0	0	0	1	0	0	1	1
734	3192		Conserved hypothetical protein	4	4	1	1	0	0	1	0	0	0	0	0	1	0	0	0
735	1998		Short-chain dehydrogenase/reductase family enzyme	4	5	1	1	0	0	0	2	1	0	0	0	0	0	0	0
736	2523		Conserved hypothetical protein	4	4	1	1	0	0	0	0	0	1	0	1	0	0	0	0
737	1806		Autotransporter beta-domain-containing protein	4	8	3	2	0	0	0	0	0	2	0	0	1	0	0	0
738	2115	rpcG	Possible phycobilin:phycocyanin lyase-isomerase	4	4	1	0	1	1	0	0	0	1	0	0	0	0	0	0
739	1930		Conserved hypothetical protein	4	6	1	0	1	1	0	0	0	2	0	0	0	1	0	0
740	1763		Phage integrase family protein	4	8	1	0	2	4	0	0	0	0	0	0	1	0	0	0
741	2274		Conserved hypothetical protein	4	4	1	0	1	0	1	0	0	0	0	0	1	0	0	0
742	2122		Possible nuclease	4	5	1	0	1	0	1	0	0	0	0	0	1	1	0	0
743	2268		UDP-N-acetylglucosamine 2-epimerase	4	4	1	0	0	1	1	0	0	1	0	0	0	0	0	0
744	1748		Possible N-acetylneuraminic acid synthetase	4	5	1	0	0	1	1	0	0	1	0	0	0	0	0	1
745	7768		Conserved hypothetical protein	4	4	1	0	0	0	1	0	0	1	1	0	0	0	0	0
746	1999		Bacterial acid phosphatase	4	5	1	0	0	0	0	1	1	1	0	0	0	0	0	1
747	2001		Conserved hypothetical protein	4	5	1	0	0	0	0	0	1	0	1	2	0	0	0	0
748	2239		Conserved hypothetical protein	4	4	0	1	1	1	0	0	1	0	0	0	0	0	0	0
749	2224	mscS5	Possible large-conductance mechanosensitive channel mscL	4	5	0	1	0	1	0	1	0	0	1	0	0	0	0	1
750	2512		ChaA Ca2+/H+ antiporte	4	4	0	1	0	0	1	0	1	0	0	1	0	0	0	0
751	2413		Conserved hypothetical protein	4	4	0	0	1	1	1	0	0	1	0	0	0	0	0	0
752	2406		Conserved hypothetical protein	4	4	0	0	1	1	1	0	0	1	0	0	0	0	0	0
753	2244		Conserved hypothetical protein	4	4	0	0	1	1	0	1	1	0	0	0	0	0	0	0
754	2245		Conserved hypothetical protein	4	4	0	0	1	1	0	1	1	0	0	0	0	0	0	0
755	1875		Fatty acid desaturase	4	7	0	0	1	1	0	1	1	0	0	0	0	1	1	1
756	2211		Conserved hypothetical protein	4	4	0	0	1	1	0	1	0	1	0	0	0	0	0	0
757	2252		Conserved hypothetical protein	4	4	0	0	1	1	0	0	1	0	0	1	0	0	0	0
758	2253		Conserved hypothetical protein	4	4	0	0	1	1	0	0	1	0	0	1	0	0	0	0
759	2254		Conserved hypothetical protein	4	4	0	0	1	1	0	0	1	0	0	1	0	0	0	0
760	2255		Conserved hypothetical protein	4	4	0	0	1	1	0	0	1	0	0	1	0	0	0	0
761	2257		Conserved hypothetical protein	4	4	0	0	1	1	0	0	1	0	0	1	0	0	0	0
762	2258		Possible flavoprotein involved in K+ transport (COG2072)	4	4	0	0	1	1	0	0	1	0	0	1	0	0	0	0
763	2256		Possible nitrilase	4	4	0	0	1	1	0	0	1	0	0	1	0	0	0	0
764	1949		Conserved hypothetical protein	4	6	0	0	1	1	0	0	0	1	0	0	1	0	1	1
765	8064		ABC-type nitrate/sulfonate/bicarbonate transport system, periplasmic component	4	4	0	0	1	1	0	0	0	0	0	1	1	0	0	0
766	2247		Agmatinase	4	4	0	0	1	1	0	0	0	0	0	1	1	0	0	0
767	2259		Conserved hypothetical protein	4	4	0	0	1	1	0	0	0	0	0	1	1	0	0	0
768	2098		Hydrogenase nickel incorporation protein HypB	4	5	0	0	1	1	0	0	0	0	0	2	1	0	0	0
769	2251		Possible dioxygenase	4	4	0	0	1	1	0	0	0	0	0	1	1	0	0	0
770	2248		Possible hydrogenase nickel incorporation protein hypA	4	4	0	0	1	1	0	0	0	0	0	1	1	0	0	0
771	2250		Possible nitrate ABC transporter, membrane component	4	4	0	0	1	1	0	0	0	0	0	1	1	0	0	0
772	2989		Conserved hypothetical protein	4	4	0	0	1	0	1	1	1	0	0	0	0	0	0	0
773	2390		Large-conductance mechanosensitive channel	4	4	0	0	1	0	1	1	0	1	0	0	0	0	0	0
774	2328		Conserved hypothetical protein	4	7	0	0	1	0	1	0	0	1	0	0	1	1	1	1
775	4719		Conserved hypothetical protein	4	4	0	0	1	0	1	0	0	1	0	0	1	0	0	0

Line No.	Cluster No. in cyanorak	Gene Name	Product	No. of *Synechococcus* strains per cluster	No. of sequ. per cluster (14 genomes)	Synechococcus: Subcluster 5.1A: BL107	CC9902	CC9605	WH8102	CC9311	Subcluster 5.1B: WH7803	WH7805	RS9916	RS9917	5.2: WH5701	5.3: RCC307	Prochlorococcus: HL: MED4	LL: SS120	MIT9313
776	2404		Conserved hypothetical protein	4	4	0	0	1	0	1	0	0	1	0	0	1	0	0	0
777	4450		Conserved hypothetical protein	4	4	0	0	1	0	1	0	0	1	0	0	1	0	0	0
778	2415		Conserved hypothetical membrane protein	4	6	0	0	2	0	0	1	2	1	0	0	0	0	0	0
779	2217		Conserved hypothetical protein	4	4	0	0	1	0	0	1	1	1	0	0	0	0	0	0
780	4617		Conserved hypothetical protein	4	4	0	0	1	0	0	1	1	1	0	0	0	0	0	0
781	2078		Conserved hypothetical protein	4	5	0	0	2	0	0	1	1	0	1	0	0	0	0	0
782	2403		Conserved hypothetical protein	4	5	0	0	2	0	0	1	0	1	0	0	1	0	0	0
783	2391		Conserved hypothetical protein	4	4	0	0	0	1	1	0	0	1	1	0	0	0	0	0
784	2392		Conserved hypothetical protein	4	5	0	0	0	1	1	0	0	1	2	0	0	0	0	0
785	9011		Conserved hypothetical protein	4	4	0	0	0	1	1	0	0	0	1	1	0	0	0	0
786	2223		Conserved hypothetical protein	4	6	0	0	0	2	2	0	0	0	1	1	0	0	0	0
787	8026		ABC-type Mn2+/Zn2+ transport system permease component	4	4	0	0	0	1	1	0	0	0	1	0	1	0	0	0
788	8084		ABC-type Mn2+/Zn2+ transport systems, ATPase component	4	4	0	0	0	1	1	0	0	0	1	0	1	0	0	0
789	2462		Possible ABC zinc transport system substrate-binding protein	4	4	0	0	0	1	1	0	0	0	1	0	1	0	0	0
790	3025		Conserved hypothetical protein	4	4	0	0	0	1	0	1	1	0	1	0	0	0	0	0
791	1962		Conserved hypothetical protein	4	6	0	0	0	1	0	0	0	1	1	0	2	0	1	0
792	8025		ABC-type multidrug transport system, ATPase and permease components	4	6	0	0	0	1	0	0	0	1	1	0	1	1	1	0
793	2055		Conserved hypothetical protein	4	6	0	0	0	0	1	2	2	1	0	0	0	0	0	0
794	2185		Conserved hypothetical protein	4	5	0	0	0	0	1	1	1	1	0	0	0	0	1	0
795	2395		Possible carboxylesterase	4	4	0	0	0	0	1	1	1	1	0	0	0	0	0	0
796	2472		Conserved hypothetical protein	4	4	0	0	0	0	1	1	1	0	1	0	0	0	0	0
797	4833		Conserved hypothetical protein	4	5	0	0	0	0	1	1	1	0	2	0	0	0	0	0
798	2437		Indole-3-pyruvate decarboxylase	4	4	0	0	0	0	1	1	1	0	1	0	0	0	0	0
799	2033		Two-component system sensor histidine kinase	4	6	0	0	0	0	1	1	2	0	1	0	0	0	0	1
800	2499		Conserved hypothetical membrane protein	4	5	0	0	0	0	1	1	1	0	0	1	0	1	0	0
801	2505		Glutamine synthetase III	4	4	0	0	0	0	1	1	1	0	0	1	0	0	0	0
802	8125		Possible peptidase family protein with a signal peptide	4	4	0	0	0	0	1	1	1	0	0	1	0	0	0	0
803	2502		SAM-dependent methyltransferase	4	4	0	0	0	0	1	1	1	0	0	1	0	0	0	0
804	2488		Conserved hypothetical protein	4	4	0	0	0	0	1	1	1	0	0	0	1	0	0	0
805	2494		Conserved hypothetical secreted proteir	4	4	0	0	0	0	1	1	1	0	0	0	1	0	0	0
806	4368		Conserved hypothetical protein	4	4	0	0	0	0	1	1	0	1	1	0	0	0	0	0
807	5089		Conserved hypothetical protein	4	4	0	0	0	0	1	1	0	1	1	0	0	0	0	0
808	2409		Gluconolactonase	4	4	0	0	0	0	1	1	0	1	1	0	0	0	0	0
809	2177		Conserved hypothetical membrane protein	4	5	0	0	0	0	1	1	0	1	0	0	1	0	0	1
810	2422		Possible esterase	4	4	0	0	0	0	1	1	0	0	1	1	0	0	0	0
811	2423		Probable alcohol dehydrogenase class III (Glutathione-dependent formaldehyde dehydrogenase)	4	4	0	0	0	0	1	1	0	0	1	1	0	0	0	0
812	2458		Conserved hypothetical membrane protein	4	4	0	0	0	0	1	1	0	0	1	0	1	0	0	0
813	8129		Glucose 1-dehydrogenase	4	4	0	0	0	0	1	1	0	0	1	0	1	0	0	0
814	4347		Conserved hypothetical protein	4	4	0	0	0	0	1	0	1	1	0	1	0	0	0	0
815	3740		Conserved hypothetical membrane protein	4	4	0	0	0	0	1	0	0	1	0	0	1	0	0	1
816	2365		Possible Tripartite transporter component (TRAP-T family)	4	4	0	0	0	0	1	0	0	1	0	0	1	0	0	1
817	2366		Possible Tripartite transporter component (TRAP-T family)	4	4	0	0	0	0	1	0	0	1	0	0	1	0	0	1
818	2364		Possible Tripartite transporter component (TRAP-T family), substrate binding protein	4	4	0	0	0	0	1	0	0	1	0	0	1	0	0	1
819	1933		Conserved hypothetical membrane protein	4	6	0	0	0	0	0	1	1	1	2	0	0	1	0	0
820	2215		Conserved hypothetical protein	4	4	0	0	0	0	0	1	1	1	1	0	0	0	0	0
821	2218		Conserved hypothetical protein	4	4	0	0	0	0	0	1	1	1	1	0	0	0	0	0
822	2219		Conserved hypothetical protein	4	4	0	0	0	0	0	1	1	1	1	0	0	0	0	0
823	2393		Conserved hypothetical protein	4	4	0	0	0	0	0	1	1	1	1	0	0	0	0	0
824	4862		Conserved hypothetical protein	4	4	0	0	0	0	0	1	1	1	1	0	0	0	0	0
825	3029		Conserved hypothetical secreted protein	4	4	0	0	0	0	0	1	1	1	1	0	0	0	0	0
826	2043		Conserved hypothetical protein	4	5	0	0	0	0	0	1	1	0	1	1	0	0	0	1
827	2227		Phosphoketolase	4	4	0	0	0	0	0	1	1	0	1	1	0	0	0	0
828	2452		Conserved hypothetical membrane protein	4	4	0	0	0	0	0	1	1	0	1	0	1	0	0	0
829	1819		Conserved hypothetical protein	4	8	0	0	0	0	0	4	1	0	1	0	1	0	0	1
830	2455		Conserved hypothetical protein	4	4	0	0	0	0	0	1	1	0	1	0	1	0	0	0
831	2457		Conserved hypothetical protein	4	4	0	0	0	0	0	1	1	0	1	0	1	0	0	0
832	2476		Conserved hypothetical secreted protein	4	4	0	0	0	0	0	1	1	0	1	0	1	0	0	0
833	2234		Drug/metabolite transporter (DMT) superfamily efflux protein	4	4	0	0	0	0	0	1	1	0	1	0	1	0	0	0
834	2456		Possible biopolymer transport protein	4	4	0	0	0	0	0	1	1	0	1	0	1	0	0	0
835	2262		Conserved hypothetical protein	4	4	0	0	0	0	0	1	1	0	0	1	1	0	0	0
836	1873	gltS	Possible Na+/glutamate symporter	4	7	0	0	0	0	0	1	1	0	0	1	1	1	1	1
837	2852		Conserved hypothetical membrane protein	4	4	0	0	0	0	0	1	0	0	1	1	1	0	0	0
838	2198		Conserved hypothetical membrane protein	4	4	0	0	0	0	0	0	1	1	1	1	0	0	0	0
839	2006		Conserved hypothetical protein	5	6	1	1	2	1	1	0	0	0	0	0	0	0	0	0
840	2007		Conserved hypothetical protein	5	6	1	1	1	2	1	0	0	0	0	0	0	0	0	0
841	2146		Conserved hypothetical protein	5	5	1	1	1	1	1	0	0	0	0	0	0	0	0	0
842	2270		Conserved hypothetical protein	5	5	1	1	1	1	1	0	0	0	0	0	0	0	0	0
843	1842		Hydrogenase accessory protein or high-affinity nickel-transport protein homolog	5	8	1	1	1	1	1	0	0	0	0	0	0	1	1	1
844	1843	sodN	Nickel-containing superoxide dismutase	5	8	1	1	1	1	1	0	0	0	0	0	0	1	1	1
845	2129	dvr	Possible 3,8-Divinyl Protochlorophyllide a 8-Vinyl Reductase	5	5	1	1	1	1	1	0	0	0	0	0	0	0	0	0
846	1907		Possible phosphoribosyltransferase	5	7	1	1	1	1	1	0	0	0	0	0	0	1	1	0
847	2125		Signal peptidase I	5	8	1	1	1	1	1	0	0	0	0	0	0	1	1	1
848	1928		Conserved hypothetical protein	5	6	1	1	1	2	0	0	0	1	0	0	0	0	0	0
849	2013		Conserved hypothetical protein	5	5	1	1	1	1	0	0	0	1	0	0	0	0	0	0
850	2018		Conserved hypothetical protein	5	5	1	1	1	1	0	0	0	1	0	0	0	0	0	0
851	2622		Conserved hypothetical protein	5	5	1	1	1	1	0	0	0	1	0	0	0	0	0	0
852	2002		Conserved hypothetical protein	5	5	1	1	1	1	0	0	0	0	0	1	0	0	0	0
853	61	ispF	tRNA-(guanine-N1)-methyltransferase / 2-C-methyl-D-erythritol 2,4-cyclodiphosphate synthase	5	8	1	1	1	1	0	0	0	0	0	1	0	1	1	1
854	1865		Possible succinate dehydrogenase cytochrome b-556 subunit	5	7	1	1	1	1	0	0	0	0	0	1	0	0	1	1
855	1867	sdhB	Succinate dehydrogenase/fumarate reductase, Fe-S protein subunit	5	7	1	1	1	1	0	0	0	0	0	1	0	0	1	1
856	1866	sdhA	Succinate dehydrogenase/fumarate reductase, flavoprotein subunit	5	7	1	1	1	1	0	0	0	0	0	1	0	0	1	1
857	1918		Possible dehydrogenase (flavoprotein)	5	6	1	1	1	1	0	0	0	0	0	0	1	0	0	1
858	2281		Conserved hypothetical protein	5	5	1	1	1	0	1	0	0	1	0	0	0	0	0	0
859	2576		Conserved hypothetical secreted protein	5	8	1	1	1	0	3	0	0	0	0	0	2	0	0	0
860	1911		Conserved hypothetical protein	5	6	1	1	1	0	0	1	0	1	0	0	0	0	0	1
861	1997		Conserved hypothetical protein	5	5	1	1	1	0	0	0	0	1	1	0	0	0	0	0
862	2318		Conserved hypothetical protein	5	5	1	1	0	1	1	0	0	1	0	0	0	0	0	0
863	2009		Possible two-component system response regulator	5	5	1	1	0	1	0	0	1	0	0	0	1	0	0	0
864	3051		Conserved hypothetical protein	5	6	1	1	0	0	2	1	1	0	0	0	0	0	0	0
865	3165		Conserved hypothetical protein	5	6	1	1	0	0	2	0	1	1	0	0	0	0	0	0
866	1994		Alpha-acetolactate decarboxylase	5	6	1	1	0	0	1	0	0	0	2	0	1	0	0	0
867	2123		AraC-type DNA-binding domain-containing protein	5	5	1	1	0	0	1	0	0	1	0	0	1	0	0	0
868	2124		AraC-type DNA-binding domain-containing protein	5	5	1	1	0	0	1	0	0	1	0	0	1	0	0	0
869	9110	mpeZ	Possible phycobilin:C-phycoerythrin II lyase	5	5	1	1	0	0	1	0	0	1	0	0	1	0	0	0
870	1993		Conserved hypothetical membrane protein	5	5	1	1	0	0	0	1	1	0	0	0	1	0	0	0
871	3166		Protein chain release factor B	5	5	1	1	0	0	0	0	0	1	0	1	1	0	0	0
872	2604		Conserved hypothetical membrane protein	5	5	1	0	1	1	1	0	0	0	0	0	1	0	0	0
873	2022		PhoH family protein	5	5	1	0	1	1	0	0	0	1	0	1	0	0	0	0
874	1831		Glutamine amidotransferase class-I	5	8	1	0	1	0	1	0	0	1	1	0	0	1	1	1
875	1430		Conserved hypothetical protein	5	11	2	0	2	0	0	2	2	3	0	0	0	0	0	0
876	2112		orotidine 5 prime-phosphate decarboxylase/orotate phosphoribosyltransferase	5	5	1	0	0	0	1	0	1	1	0	1	0	0	0	0
877	2243		Conserved hypothetical protein	5	5	0	1	1	1	0	1	1	0	0	0	0	0	0	0
878	1940		Possible hydrolase of the alpha/beta-hydrolase fold	5	6	0	1	0	1	0	1	0	1	0	0	1	0	0	1
879	3005		Conserved hypothetical membrane protein	5	5	0	1	0	0	0	1	1	1	1	0	0	0	0	0
880	3003		Conserved hypothetical protein	5	5	0	1	0	0	0	1	1	1	0	0	1	0	0	0
881	2246		Conserved hypothetical protein	5	5	0	0	1	1	1	1	1	0	0	0	0	0	0	0
882	2886		Metallothionein	5	9	0	0	1	1	4	1	0	0	0	2	0	0	0	0
883	2249		Possible nitrate ABC transporter, substrate binding protein	5	5	0	0	1	1	1	0	0	0	0	1	1	0	0	0
884	1876		Conserved hypothetical protein	5	7	0	0	1	1	0	1	1	0	0	0	1	1	1	0
885	8044		Possible ABC multidrug efflux transporter	5	7	0	0	1	1	0	1	0	2	0	0	0	0	0	2
886	2071		Conserved hypothetical protein	5	5	0	0	1	1	0	0	0	1	0	1	1	0	0	0

						Synechococcus											Prochlorococcus		
						Subcluster 5.1A					Subcluster 5.1B				5.2	5.3	HL		LL
Line No.	Cluster No. in cyanorak	Gene Name	Product	No. of *Synechococcus* strains per cluster	No. of sequ. per cluster (14 genomes)	BL107	CC9902	CC9605	WH8102	CC9311	WH7803	WH7805	RS9916	RS9917	WH5701	RCC307	MED4	SS120	MIT9313
887	2500		Conserved hypothetical secreted protein	5	9	0	0	2	0	1	2	2	0	0	0	2	0	0	0
888	2049	CRP	Possible cAMP or cGMP receptor protein	5	6	0	0	1	0	1	0	1	1	1	0	0	0	0	1
889	2181		Conserved hypothetical protein	5	5	0	0	1	0	1	0	1	0	0	1	0	0	0	1
890	1959		Possible adenylate/guanylate cyclase	5	7	0	0	1	0	1	0	1	0	0	2	0	0	0	2
891	2084		Deoxycytidine triphosphate deaminase	5	5	0	0	1	0	0	1	1	0	1	1	0	0	0	0
892	2082		Conserved hypothetical protein	5	5	0	0	1	0	0	1	1	0	1	0	1	0	0	0
893	2096		Conserved hypothetical protein	5	5	0	0	1	1	0	1	1	0	0	0	1	0	0	0
894	2095		Conserved hypothetical membrane protein	5	5	0	0	1	1	0	1	1	0	0	0	1	0	0	0
895	2213		Conserved hypothetical membrane protein	5	5	0	0	1	0	0	1	0	1	1	1	0	0	0	0
896	2214		Possible Multisubunit Na+/H+ antiporter subunit	5	5	0	0	1	0	0	1	0	1	1	1	0	0	0	0
897	2076		Possible Multisubunit Na+/H+ antiporter, MnhB subunit	5	5	0	0	1	0	0	1	0	1	1	1	0	0	0	0
898	2074		Possible Multisubunit Na+/H+ antiporter, MnhC subunit	5	5	0	0	1	0	0	1	0	1	1	1	0	0	0	0
899	2212		Possible Multisubunit Na+/H+ antiporter, MnhD subunit	5	5	0	0	1	0	0	1	0	1	1	1	0	0	0	0
900	2075		Possible Multisubunit Na+/H+ antiporter, MnhG subunit	5	5	0	0	1	0	0	1	0	1	1	1	0	0	0	0
901	4316		Conserved hypothetical membrane protein	5	5	0	0	1	0	0	0	1	1	1	0	1	0	0	0
902	9020		Conserved hypothetical protein	5	6	0	0	0	1	1	1	1	0	0	1	0	0	0	1
903	2039		Permease	5	6	0	0	0	1	1	0	1	0	0	1	1	0	0	1
904	2051		Nucleotide-diphosphate-sugar epimerase, membrane associated	5	6	0	0	0	1	1	0	0	0	1	1	1	0	1	0
905	1943	proW	ABC glycine betaine/proline transporter, membrane component	5	6	0	0	0	1	0	1	1	1	1	0	0	0	0	1
906	1944	proX	ABC glycine betaine/proline transporter, substrate binding component	5	6	0	0	0	1	0	1	1	1	1	0	0	0	0	1
907	8061	proV	ABC-type proline/glycine betaine transport system, ATPase component	5	6	0	0	0	1	0	1	1	1	1	0	0	0	0	1
908	1942	gbmt1	Glycine-sarcosine methyltransferase	5	6	0	0	0	1	0	1	1	1	1	0	0	0	0	1
909	1941	gbmt2	Sarcosine-dimethylglycine methyltransferase	5	6	0	0	0	1	0	1	1	1	1	0	0	0	0	1
910	2081		Acetate kinase	5	5	0	0	0	1	0	1	1	0	1	1	0	0	0	0
911	2086		Conserved hypothetical protein	5	5	0	0	0	1	0	1	1	0	1	0	1	0	0	0
912	2088		Conserved hypothetical secreted protein	5	5	0	0	0	1	0	1	1	0	1	0	1	0	0	0
913	1985		Dienelactone hydrolase family protein	5	6	0	0	0	1	0	1	2	0	1	0	1	0	0	0
914	2083		Possible low molecular weight phosphotyrosine protein phosphatase	5	5	0	0	0	1	0	1	1	0	1	0	1	0	0	0
915	2774		Conserved hypothetical protein	5	5	0	0	0	1	0	1	0	1	1	1	0	0	0	0
916	2475		Conserved hypothetical protein	5	7	0	0	0	1	0	1	0	1	3	0	1	0	0	0
917	1829	sphX	ABC transporter, substrate binding protein, phosphate, cyanobacterial specific	5	8	0	0	0	1	0	1	0	0	2	3	1	0	0	0
918	2092	corA	Possible cation (Mg/Co) transporter, MIT family	5	6	0	0	0	2	0	1	0	0	1	1	1	0	0	0
919	2803		Possible transcription regulator	5	6	0	0	0	2	0	0	1	0	1	1	0	0	0	1
920	2376		Conserved hypothetical protein	5	5	0	0	0	1	1	0	0	1	1	1	0	0	0	0
921	2209		Conserved hypothetical membrane protein	5	5	0	0	0	0	1	1	1	1	1	0	0	0	0	0
922	2976		Conserved hypothetical membrane protein	5	5	0	0	0	0	1	1	1	1	1	0	0	0	0	0
923	2199		Conserved hypothetical protein	5	5	0	0	0	0	1	1	1	1	1	0	0	0	0	0
924	4386		Conserved hypothetical protein	5	5	0	0	0	0	1	1	1	1	1	0	0	0	0	0
925	4489		Conserved hypothetical protein	5	5	0	0	0	0	1	1	1	1	1	0	0	0	0	0
926	3026		Conserved hypothetical secreted protein	5	6	0	0	0	0	1	1	1	1	1	0	0	0	0	1
927	2050		Ecotin	5	6	0	0	0	0	1	1	1	1	1	0	0	0	0	1
928	1895		Conserved hypothetical protein	5	8	0	0	0	0	1	2	2	1	0	2	0	0	0	0
929	1972		Conserved hypothetical secreted protein	5	7	0	0	0	0	1	1	1	1	0	3	0	0	0	0
930	1861		sodium/bile acid cotransporter family protein	5	5	0	0	0	0	1	1	1	1	0	1	0	0	0	0
931	9005		Possible protein kinase	5	5	0	0	0	0	1	1	1	1	0	0	1	0	0	0
932	2203		Two-component system sensor histidine kinase	5	5	0	0	0	0	1	1	1	1	0	0	1	0	0	0
933	2090		Glutamate-ammonia ligase	5	6	0	0	0	0	1	1	1	0	2	1	0	0	0	0
934	2229		carbohydrate-binding module family 2-containing protein	5	5	0	0	0	0	1	1	1	0	1	0	1	0	0	0
935	2231		Carbohydrate-selective porin OprB related protein	5	5	0	0	0	0	1	1	1	0	1	0	1	0	0	0
936	2990		Conserved hypothetical membrane protein	5	5	0	0	0	0	1	1	1	0	1	0	1	0	0	0
937	2089		conserved hypothetical protein distantly related to glycosidases	5	6	0	0	0	0	1	1	1	0	2	0	1	0	0	0
938	2473		Conserved hypothetical secreted protein	5	5	0	0	0	0	1	1	1	0	1	0	1	0	0	0
939	3893		Conserved hypothetical protein	5	6	0	0	0	0	1	1	1	0	0	1	1	0	0	1
940	2030		Conserved hypothetical membrane protein	5	7	0	0	0	0	2	1	0	1	1	0	1	0	0	1
941	2040		Conserved hypothetical membrane protein	5	6	0	0	0	0	1	1	0	1	1	0	1	0	0	1
942	2041		Conserved hypothetical membrane protein	5	6	0	0	0	0	1	1	0	1	1	0	1	0	0	1
943	2042		Conserved hypothetical membrane protein	5	6	0	0	0	0	1	1	0	1	1	0	1	0	0	1
944	2202		Conserved hypothetical membrane protein	5	5	0	0	0	0	1	1	0	1	1	0	1	0	0	0
945	2400		Conserved hypothetical membrane protein	5	5	0	0	0	0	1	1	0	1	1	0	1	0	0	0
946	2401		Conserved hypothetical membrane protein	5	5	0	0	0	0	1	1	0	1	1	0	1	0	0	0
947	2038		Conserved hypothetical protein	5	6	0	0	0	0	1	1	0	1	1	0	1	0	0	1
948	2031		Conserved hypothetical secreted protein	5	7	0	0	0	0	2	1	0	1	1	0	1	0	0	1
949	2342		Conserved hypothetical secreted protein	5	7	0	0	0	0	2	1	0	1	1	0	1	0	0	1
950	2671		Conserved hypothetical secreted protein	5	6	0	0	0	0	1	1	0	1	1	0	1	0	0	1
951	3651		Conserved hypothetical secreted protein	5	6	0	0	0	0	1	1	0	1	1	0	1	0	0	1
952	2067		Membrane-fusion protein	5	6	0	0	0	0	1	1	0	1	2	0	1	0	0	0
953	2068		Outer membrane efflux protein	5	6	0	0	0	0	1	1	0	1	2	0	1	0	0	0
954	2190		Conserved hypothetical protein	5	5	0	0	0	0	1	0	1	1	1	1	0	0	0	0
955	3761		Conserved hypothetical protein	5	5	0	0	0	0	1	0	1	1	1	0	0	0	0	1
956	2175		Glutamate decarboxylase	5	5	0	0	0	0	1	0	0	1	0	1	1	0	0	1
957	1932		Possible membrane associated acyltransferase	5	7	0	0	0	0	1	0	0	0	0	2	1	1	2	0
958	2351	mscL	Possible large-conductance mechanosensitive channel MscL	5	5	0	0	0	0	1	0	0	0	1	1	1	0	0	1
959	2072		Zn-dependent alcohol dehydrogenases	5	5	0	0	0	0	0	1	1	1	1	1	0	0	0	0
960	3556		Conserved hypothetical protein	5	8	0	0	0	0	0	1	1	1	1	0	1	1	1	1
961	1963		Metallo-beta-lactamase superfamily hydrolase	5	6	0	0	0	0	0	1	1	1	0	1	1	0	1	0
962	4321		Conserved hypothetical protein	5	5	0	0	0	0	0	1	1	1	1	1	0	0	0	0
963	1973		beta-glycosidase (chitinase-like), family 18	5	5	0	0	0	0	0	1	1	1	1	0	1	0	0	0
964	4313		Conserved hypothetical protein	5	6	0	0	0	0	0	1	1	1	1	0	2	0	0	0
965	2059		Fructose-bisphosphate aldolase class I	5	5	0	0	0	0	0	1	1	1	1	0	1	0	0	0
966	9109	cpcT	phycocyanobilin:Cys-84 beta-phycocyanin lyase	5	5	0	0	0	0	0	0	1	1	1	1	1	0	0	0
967	1870		Conserved hypothetical protein	6	17	1	1	7	3	1	0	0	4	0	0	0	0	0	0
968	2005		Conserved hypothetical protein	6	6	1	1	1	1	1	0	0	1	0	0	0	0	0	0
969	1704		Conserved hypothetical protein	6	10	2	1	2	1	1	0	0	0	0	0	2	1	0	0
970	1798		Conserved hypothetical protein	6	8	1	1	1	1	0	0	0	1	0	0	1	0	1	1
971	1803	mscS1	small mechanosensitive ion channel, MscS family	6	8	1	1	1	1	0	0	1	0	0	1	0	1	1	0
972	2607		Conserved hypothetical protein	6	6	1	1	1	0	1	1	1	0	0	0	0	0	0	0
973	1905		Conserved hypothetical protein	6	8	1	1	1	0	1	0	1	1	0	0	0	0	1	1
974	1777		Conserved hypothetical protein	6	8	1	1	1	0	0	0	1	1	0	0	1	0	1	1
975	1995		Conserved hypothetical protein	6	6	1	1	1	0	0	1	1	1	0	0	0	0	0	0
976	1773		Conserved hypothetical protein	6	8	1	1	2	0	0	1	1	1	0	0	0	0	0	1
977	1917		Conserved hypothetical protein	6	6	1	1	1	1	0	0	0	1	0	0	1	0	0	0
978	276		alpha-glycosyltransferase, family 4	6	13	2	3	0	3	0	2	1	0	0	2	0	0	0	0
979	2021		Conserved hypothetical membrane protein	6	6	1	1	0	0	1	1	1	0	1	0	0	0	0	0
980	2017		Conserved hypothetical secreted protein	6	6	1	1	0	0	1	1	1	0	1	0	0	0	0	0
981	8854		Conserved hypothetical secreted protein	6	7	1	1	0	0	2	1	1	0	1	0	0	0	0	0
982	117		Conserved hypothetical membrane protein	6	14	1	2	0	0	0	2	3	3	2	0	0	0	0	1
983	2099		Hypothetical protein	6	18	1	0	1	1	6	2	7	0	0	0	0	0	0	0
984	4785		Conserved hypothetical membrane protein	6	6	1	0	1	0	1	1	0	1	1	0	0	0	0	0
985	154		Conserved hypothetical protein	6	14	2	0	1	0	0	2	2	2	0	3	0	0	0	2
986	1927		Conserved hypothetical protein	6	6	1	0	1	0	0	1	1	1	0	0	1	0	0	0
987	2424		Conserved hypothetical protein	6	7	1	0	1	0	0	0	1	0	1	2	1	0	0	0
988	7770		Conserved hypothetical protein	6	6	1	0	0	0	1	0	1	1	1	1	0	0	0	0
989	1863		Conserved hypothetical membrane protein	6	8	1	0	0	0	1	0	1	1	0	1	1	1	1	0
990	2454		Conserved hypothetical protein	6	7	0	1	0	2	0	1	1	1	1	0	0	0	0	0
991	3050		Conserved hypothetical protein	6	6	0	1	0	0	1	1	1	0	1	0	1	0	0	0
992	2493		Conserved hypothetical protein	6	6	0	0	1	1	1	1	0	1	0	0	1	0	0	0
993	1986		Possible CaCA family calcium/proton exchanger	6	6	0	0	1	1	1	1	1	0	0	0	1	0	0	0
994	2085		Conserved hypothetical membrane protein	6	6	0	0	1	1	1	0	1	0	1	1	0	0	0	0
995	4989		Conserved hypothetical protein	6	6	0	0	1	1	1	0	0	1	1	0	1	0	0	0
996	2062		Conserved hypothetical protein	6	6	0	0	1	1	0	1	1	1	1	0	0	0	0	0
997	2230		Conserved hypothetical protein	6	6	0	0	1	1	0	1	1	1	1	0	0	0	0	0

						Synechococcus											Prochlorococcus		
						Subcluster 5.1A					Subcluster 5.1B				5.2	5.3	HL		LL
Line No.	Cluster No. in cyanorak	Gene Name	Product	No. of *Synechococcus* strains per cluster	No. of sequ. per cluster (14 genomes)	BL107	CC9902	CC9605	WH8102	CC9311	WH7803	WH7805	RS9916	RS9917	WH5701	RCC307	MED4	SS120	MIT9313
998	3013		Conserved hypothetical protein	6	6	0	0	1	1	0	1	1	0	1	1	0	0	0	0
999	1887		Possible phosphohydrolase	6	7	0	0	1	1	0	1	1	0	1	1	0	0	0	1
1000	1815	arsB	arsenate efflux pump	6	8	0	0	1	1	0	1	1	0	2	0	1	1	0	0
1001	1731		Conserved hypothetical protein	6	9	0	0	1	1	0	1	1	0	1	0	1	1	1	1
1002	2379		Conserved hypothetical protein	6	7	0	0	1	1	0	1	0	1	1	1	0	0	1	0
1003	4687		Conserved hypothetical protein	6	7	0	0	1	0	1	1	2	1	0	0	1	0	0	0
1004	1961		Possible transcriptional regulator protein	6	7	0	0	1	0	1	0	1	1	1	1	0	0	0	1
1005	1978	chrA2	chromate transporter, CHR family	6	6	0	0	1	0	0	1	1	1	1	0	1	0	0	0
1006	1742		Conserved hypothetical protein	6	10	0	0	0	1	1	1	0	1	1	5	0	0	0	0
1007	2751		Conserved hypothetical protein	6	7	0	0	0	1	1	1	0	1	1	2	0	0	0	0
1008	2063		Conserved hypothetical secreted protein	6	6	0	0	0	1	1	1	0	1	1	1	0	0	0	0
1009	2187		Conserved hypothetical protein	6	7	0	0	0	1	0	1	1	1	1	1	0	0	1	0
1010	2233		Conserved hypothetical protein	6	6	0	0	0	1	0	1	1	0	1	1	1	0	0	0
1011	3010		Conserved hypothetical membrane protein	6	7	0	0	0	0	1	1	1	1	1	1	0	0	1	0
1012	2056		Conserved hypothetical protein	6	6	0	0	0	0	1	1	1	1	1	1	0	0	0	0
1013	2445		Conserved hypothetical protein	6	6	0	0	0	0	1	1	1	1	1	1	0	0	0	0
1014	7830		Conserved hypothetical protein	6	6	0	0	0	0	1	1	1	1	1	1	0	0	0	0
1015	2427		Conserved hypothetical secreted protein	6	6	0	0	0	0	1	1	1	1	1	1	0	0	0	0
1016	2428		Conserved hypothetical secreted protein	6	6	0	0	0	0	1	1	1	1	1	1	0	0	0	0
1017	9008		Possible membrane-fusion protein	6	8	0	0	0	0	2	1	1	1	1	1	0	0	0	1
1018	9121		Two-component system response regulator	6	6	0	0	0	0	1	1	1	1	1	1	0	0	0	0
1019	2065		Two-component system sensor histidine kinase	6	6	0	0	0	0	1	1	1	1	1	1	0	0	0	0
1020	9019	ispF	2-C-methyl-D-erythritol 2,4-cyclodiphosphate synthase	6	6	0	0	0	0	1	1	1	1	1	0	1	0	0	0
1021	1934		Amino acid permease	6	7	0	0	0	0	1	1	1	1	1	0	1	0	0	1
1022	2196		Conserved hypothetical membrane protein	6	6	0	0	0	0	1	1	1	1	1	0	1	0	0	0
1023	1950		Conserved hypothetical protein	6	7	0	0	0	0	1	1	1	1	1	0	1	0	0	1
1024	2194		Conserved hypothetical protein	6	6	0	0	0	0	1	1	1	1	1	0	1	0	0	0
1025	2210		Conserved hypothetical protein	6	6	0	0	0	0	1	1	1	1	1	0	1	0	0	0
1026	2468		Conserved hypothetical protein	6	6	0	0	0	0	1	1	1	1	1	0	1	0	0	0
1027	1880		DNA repair exonuclease	6	8	0	0	0	0	1	1	1	1	1	0	1	0	1	1
1028	1879		Possible ATPase involved in DNA repair	6	8	0	0	0	0	1	1	1	1	1	0	1	0	1	1
1029	1946	clc2	Possible chloride channel	6	7	0	0	0	0	1	1	1	1	1	0	1	0	0	1
1030	2060		Possible L-fuculose phosphate aldolase	6	6	0	0	0	0	1	1	1	1	1	0	1	0	0	0
1031	1656	proP	Possible proline/betaine transporter, MFS family	6	11	0	0	0	0	1	3	1	1	3	0	1	0	0	1
1032	9018	trmD	tRNA-(guanine-N1)-methyltransferase	6	6	0	0	0	0	1	1	1	1	1	0	1	0	0	0
1033	1954		Possible bacterial sugar transferase	6	7	0	0	0	0	1	1	1	1	0	1	1	0	0	1
1034	1960		Catabolite gene activator and regulatory subunit of cAMP-dependent protein kinase	6	7	0	0	0	0	1	1	1	1	0	1	1	0	0	1
1035	2087		Alpha/beta superfamily hydrolase	6	6	0	0	0	0	1	1	1	0	1	1	1	0	0	0
1036	2206		Conserved hypothetical protein	6	6	0	0	0	0	1	1	1	0	1	1	1	0	0	0
1037	1874		alpha-glycosidase	6	9	0	0	0	0	1	1	1	1	1	0	1	1	1	1
1038	1814		Conserved hypothetical protein	6	9	0	0	0	0	1	1	1	1	1	1	0	1	1	1
1039	3340		Conserved hypothetical membrane protein	6	9	0	0	0	0	1	1	0	1	1	1	1	1	1	1
1040	3426		Conserved hypothetical protein	6	8	0	0	0	0	1	1	0	1	1	1	1	1	1	0
1041	1974		Coenzyme F420-reducing hydrogenase, beta subunit	6	6	0	0	0	0	0	1	1	1	1	1	1	0	0	0
1042	2208		Conserved hypothetical protein	6	6	0	0	0	0	0	1	1	1	1	1	1	0	0	0
1043	1884		Possible membrane protein implicated in regulation of membrane protease activity	6	7	0	0	0	0	0	1	1	1	1	1	1	0	0	1
1044	1883		Prohibitin family protein	6	7	0	0	0	0	0	1	1	1	1	1	1	0	0	1
1045	1824	sodB	Superoxide dismutase [Fe]	6	6	0	0	0	0	0	1	1	1	1	1	1	0	0	0
1046	1910		Conserved hypothetical protein	7	7	1	1	1	1	1	1	1	0	0	0	0	0	0	0
1047	1919		Uridine kinase	7	7	1	1	1	1	1	1	1	0	0	0	0	0	0	0
1048	1393	rpcT	Possible phycobilin:phycocyanin lyase	7	7	1	1	1	1	1	1	0	1	0	0	0	0	0	0
1049	2155		Conserved hypothetical protein	7	7	1	1	1	1	1	0	1	1	0	0	0	0	0	0
1050	1923		Conserved hypothetical protein	7	7	1	1	1	1	1	0	1	1	0	0	0	0	0	0
1051	2279	unk10	conserved hypothetical protein, gene in phycobilisome rod gene region	7	7	1	1	1	1	1	0	0	1	0	0	1	0	0	0
1052	2116	unk8	Conserved hypothetical protein, gene in the phycobilisome gene region	7	7	1	1	1	1	1	0	0	1	0	0	1	0	0	0
1053	1620		Conserved hypothetical protein	7	12	2	1	3	1	2	0	0	1	0	0	2	0	0	0
1054	2012		Conserved hypothetical protein	7	7	1	1	1	1	1	0	0	1	0	0	1	0	0	0
1055	1768		Nucleoside 2-deoxyribosyltransferase	7	9	1	1	1	1	1	0	0	1	0	0	1	1	1	0
1056	1921		Possible nucleoside-diphosphate-sugar epimerase	7	7	1	1	1	1	1	0	0	1	0	0	1	0	0	0
1057	136	mpeU	Possible phycobilin:C-phycoerythrin II lyase	7	7	1	1	1	1	1	0	0	1	0	0	1	0	0	0
1058	8012	mpeC	rod linker polypeptide (Lr), C-phycoerythrin II-associated (C-phycoerythrin II gamma subunit)	7	7	1	1	1	1	1	0	0	1	0	0	1	0	0	0
1059	1844		Conserved hypothetical protein	7	7	1	1	1	1	0	1	1	1	0	0	0	0	0	0
1060	9129		sodium/bile acid cotransporter family protein	7	7	1	1	1	1	0	1	1	0	1	0	0	0	0	0
1061	97		Conserved hypothetical protein	7	15	1	1	2	3	0	1	1	0	0	0	1	3	1	1
1062	1838		Conserved hypothetical protein	7	7	1	1	1	1	0	1	0	1	0	0	1	0	0	0
1063	1868		Conserved hypothetical protein	7	7	1	1	1	1	0	0	1	1	1	0	0	0	0	0
1064	1799		Conserved hypothetical protein	7	8	1	1	1	1	0	0	1	0	0	1	1	0	0	1
1065	1752		Conserved hypothetical membrane protein	7	9	1	1	2	0	1	1	1	1	0	0	0	0	0	1
1066	1914		Conserved hypothetical protein	7	8	1	1	1	0	1	1	1	2	0	0	0	0	0	0
1067	1915		Conserved hypothetical protein	7	8	1	1	2	0	1	1	1	1	0	0	0	0	0	0
1068	2128		Conserved hypothetical protein	7	8	1	1	2	0	1	1	1	1	0	0	0	0	0	0
1069	1765		Two-component system response regulator (signal receiver domain)	7	9	2	1	1	0	1	1	1	1	0	0	0	0	0	1
1070	1755		Possible hydroxylase	7	9	1	1	1	0	1	0	1	1	0	1	0	0	1	1
1071	1776		Conserved hypothetical protein	7	9	1	1	1	0	1	0	1	1	0	0	1	0	1	1
1072	1778		Possible ATPase	7	9	1	1	1	0	1	0	1	1	0	0	1	0	1	1
1073	1845		Conserved hypothetical protein	7	8	1	1	0	1	1	0	1	1	1	0	0	0	1	0
1074	1851		Conserved hypothetical protein	7	9	1	1	0	1	0	1	1	1	1	0	0	1	1	0
1075	8092		Glyceraldehyde-3-phosphate dehydrogenase	7	7	1	1	0	0	1	1	1	1	0	1	0	0	0	0
1076	8055	mpeV	Possible phycoerythrobilin:phycoerythrin lyase	7	9	1	1	0	0	1	1	1	1	0	0	1	0	1	1
1077	1772		Two-component system sensor histidine kinase	7	7	1	1	0	0	1	1	1	1	0	0	1	0	0	0
1078	1830		Conserved hypothetical membrane protein	7	8	1	1	0	0	1	1	1	0	1	1	0	0	0	1
1079	1681		Conserved hypothetical protein	7	10	2	0	1	0	1	1	2	1	0	1	0	0	0	1
1080	2284		Conserved hypothetical protein	7	8	1	0	1	1	2	1	1	1	0	0	0	0	0	0
1081	7774		Conserved hypothetical protein	7	7	1	0	0	0	1	1	1	1	1	1	0	0	0	0
1082	1654		Conserved hypothetical protein	7	10	0	1	1	1	0	1	1	1	1	0	0	1	1	1
1083	1891	pyrB	Aspartate carbamoyltransferase	7	7	0	1	1	1	0	0	1	1	1	0	1	0	0	0
1084	1893		Conserved hypothetical protein	7	7	0	1	1	1	0	0	1	1	1	0	1	0	0	0
1085	1892		N-carbamoyl-L-amino acid amidohydrolase	7	7	0	1	1	1	0	0	1	1	1	0	1	0	0	0
1086	1890		sodium:solute transporter family	7	7	0	1	1	1	0	0	1	1	1	0	1	0	0	0
1087	2064		Conserved hypothetical membrane protein	7	7	0	1	1	0	1	1	1	1	0	0	1	0	0	0
1088	2745		Conserved hypothetical membrane protein	7	7	0	1	1	0	1	1	1	1	0	0	1	0	0	0
1089	1966		Amino acid transporter	7	7	0	1	0	0	1	1	1	1	1	0	1	0	0	0
1090	1534	mscS2	small mechanosensitive ion channel, MscS family	7	8	0	1	0	0	1	1	1	1	1	0	1	0	0	1
1091	1982	rpcE	phycocyanin alpha phycobilin lyase	7	7	0	1	0	0	1	1	1	0	1	1	1	0	0	0
1092	1983	rpcF	Possible phycoerythrobilin:Cys-84 alpha R-phycocyanin II lyase, RpcF subunit	7	7	0	1	0	0	1	1	1	0	1	1	1	0	0	0
1093	1975		Possible PAS domain sensor protein	7	7	0	1	0	0	1	1	1	1	0	1	1	0	0	0
1094	2186		Conserved hypothetical membrane protein	7	7	0	1	0	0	0	1	1	1	1	1	1	0	0	0
1095	8051	hli	High light inducible protein	7	7	0	0	1	1	1	1	1	1	0	0	1	0	0	0
1096	1881		Possible carboxymethylenebutenolidase	7	8	0	0	1	1	1	1	1	1	0	0	1	0	0	1
1097	1979		Conserved hypothetical membrane protein	7	7	0	0	1	1	1	1	0	1	1	0	1	0	0	0
1098	1741		Conserved hypothetical protein	7	9	0	0	1	1	0	1	1	1	3	1	0	0	0	0
1099	2044		Possible secreted phosphohydrolase	7	8	0	0	1	0	1	1	1	1	1	1	0	0	0	1
1100	1964		Conserved hypothetical protein	7	7	0	0	1	0	1	1	1	1	1	1	0	0	0	0
1101	2070		Conserved hypothetical secreted protein	7	8	0	0	2	0	1	1	1	1	1	1	0	0	0	0
1102	1897	katG	Catalase/peroxidase	7	7	0	0	1	0	0	1	1	1	1	1	1	0	0	0
1103	1901		Conserved hypothetical secreted protein	7	7	0	0	1	0	0	1	1	1	1	1	1	0	0	0
1104	1535		Possible cation efflux transporter (CDF family)	7	7	0	0	0	1	1	1	1	1	1	1	0	0	0	0
1105	1968		SAM-dependent methyltransferase	7	7	0	0	0	1	1	1	1	1	0	1	1	0	0	0
1106	1956		Conserved hypothetical protein	7	8	0	0	0	1	1	0	1	1	1	1	1	0	0	1
1107	1882		Conserved hypothetical protein	7	8	0	0	0	1	0	1	1	1	1	1	1	0	0	1
1108	2047		Conserved hypothetical secreted protein	7	8	0	0	0	1	0	1	1	1	1	1	1	0	0	1

Line No.	Cluster No. in cyanorak	Gene Name	Product	No. of *Synechococcus* strains per cluster	No. of sequ. per cluster (14 genomes)	Synechococcus Subcluster 5.1A BL107	CC9902	CC9605	WH8102	CC9311	Subcluster 5.1B WH7803	WH7805	RS9916	RS9917	5.2 WH5701	5.3 RCC307	Prochlorococcus HL MED4	SS120	LL MIT9313
1109	1885		Conserved hypothetical membrane protein	7	8	0	0	0	0	1	1	1	1	1	1	1	0	0	1
1110	1947		Conserved hypothetical membrane protein	7	8	0	0	0	0	1	1	1	1	1	1	1	0	0	1
1111	1970		Conserved hypothetical membrane protein	7	7	0	0	0	0	1	1	1	1	1	1	1	0	0	0
1112	1734		Conserved hypothetical protein	7	10	0	0	0	0	1	1	1	1	1	1	1	1	1	1
1113	1877		Conserved hypothetical protein	7	8	0	0	0	0	1	1	1	1	1	1	1	0	0	1
1114	1896		Conserved hypothetical protein	7	8	0	0	0	0	1	2	1	1	1	1	1	0	0	0
1115	1980		Conserved hypothetical protein	7	7	0	0	0	0	1	1	1	1	1	1	1	0	0	0
1116	2723		Conserved hypothetical protein	7	7	0	0	0	0	1	1	1	1	1	1	1	0	0	0
1117	7842		Conserved hypothetical protein	7	7	0	0	0	0	1	1	1	1	1	1	1	0	0	0
1118	1971		Conserved hypothetical secreted protein	7	7	0	0	0	0	1	1	1	1	1	1	1	0	0	0
1119	2032		Conserved hypothetical secreted protein	7	8	0	0	0	0	1	1	1	1	1	1	1	0	0	1
1120	2762		Conserved hypothetical secreted protein	7	8	0	0	0	0	2	1	1	1	1	1	1	0	0	0
1121	1733		Possible enzyme of the cupin superfamily	7	10	0	0	0	0	1	1	1	1	1	1	1	1	1	1
1122	9125		Possible RND family multidrug efflux transporter	7	9	0	0	0	0	2	1	1	1	1	1	1	0	0	1
1123	1889	DpsA	potential DpsA	7	8	0	0	0	0	1	1	1	1	1	1	1	0	0	1
1124	1976	mscS4	small mechanosensitive ion channel, MscS family	7	7	0	0	0	0	1	1	1	1	1	1	1	0	0	0
1125	1841		Conserved hypothetical membrane protein	8	8	1	1	1	1	1	1	1	1	0	0	0	0	0	0
1126	1398	unk3	Conserved hypothetical protein	8	12	1	1	1	1	1	1	1	1	0	0	0	1	1	2
1127	1728		Conserved hypothetical protein	8	11	1	1	1	1	1	1	1	1	0	0	0	1	1	1
1128	1774		Conserved hypothetical protein	8	9	1	1	1	1	1	2	1	1	0	0	0	0	0	0
1129	1793		Conserved hypothetical protein	8	9	1	1	1	1	1	1	1	1	0	0	0	0	0	1
1130	1846		Conserved hypothetical protein	8	8	1	1	1	1	1	1	1	1	0	0	0	0	0	0
1131	1848		Conserved hypothetical protein	8	8	1	1	1	1	1	1	1	1	0	0	0	0	0	0
1132	1854		Conserved hypothetical protein	8	8	1	1	1	1	1	1	1	1	0	0	0	0	0	0
1133	1856		Conserved hypothetical protein	8	8	1	1	1	1	1	1	1	1	0	0	0	0	0	0
1134	1860		Conserved hypothetical protein	8	13	1	1	1	2	1	1	1	1	0	0	0	1	1	2
1135	1864		Conserved hypothetical protein	8	8	1	1	1	1	1	1	1	1	0	0	0	0	0	0
1136	1869		Conserved hypothetical protein	8	8	1	1	1	1	1	1	1	1	0	0	0	0	0	0
1137	1913		Conserved hypothetical protein	8	9	1	1	1	2	1	1	1	1	0	0	0	0	0	0
1138	2011		Conserved hypothetical protein	8	10	1	1	1	1	1	1	1	1	0	0	0	0	1	1
1139	2548	unk11	Conserved hypothetical protein, gene located in phycobilisome gene region	8	8	1	1	1	1	1	1	1	1	0	0	0	0	0	0
1140	5948	unk6	Conserved hypothetical protein, gene located in phycobilisome gene region	8	11	1	1	1	1	1	1	1	1	0	0	0	1	1	1
1141	1808		Conserved hypothetical secreted protein	8	11	1	1	1	1	1	1	1	1	0	0	0	1	1	1
1142	2269		Conserved hypothetical secreted protein	8	11	1	1	1	1	1	1	2	1	0	0	0	1	1	0
1143	8121		Ribosomal large subunit pseudouridine synthase A	8	9	1	1	1	1	1	1	1	1	0	0	0	0	0	1
1144	2154		Conserved hypothetical secreted protein	8	9	1	1	1	1	1	1	1	0	1	0	0	0	0	1
1145	1683		Possible Mn2+/Fe2+ transporter, NRAMP family	8	10	1	1	1	1	1	1	2	0	1	0	0	0	1	0
1146	2616		Conserved hypothetical membrane protein	8	8	1	1	1	1	1	1	1	0	0	1	0	0	0	0
1147	1916		DnaJ domain containing protein	8	8	1	1	1	1	1	1	1	0	0	1	0	0	0	0
1148	1551		Possible acetyltransferase	8	11	1	1	1	1	1	1	1	0	0	1	0	1	1	1
1149	1837	aplA	Allophycocyanin-like protein	8	8	1	1	1	1	1	1	0	1	0	0	1	0	0	0
1150	1835	unk9	conserved hypothetical protein, gene in phycobilisome rod gene region	8	8	1	1	1	1	1	1	0	1	0	0	1	0	0	0
1151	1836	unk7	conserved hypothetical protein, gene in phycobilisome rod gene region	8	8	1	1	1	1	1	1	0	1	0	0	1	0	0	0
1152	7994	mpeA	C-phycoerythrin class II, alpha chain	8	8	1	1	1	1	1	1	0	1	0	0	1	0	0	0
1153	8005	mpeB	C-phycoerythrin class II, beta chain	8	8	1	1	1	1	1	1	0	1	0	0	1	0	0	0
1154	1396	mpeY	Possible phycobilin:C-phycoerythrin II lyase	8	8	1	1	1	1	1	1	0	1	0	0	1	0	0	0
1155	8016	mpeE	rod linker polypeptide (Lr), C-phycoerythrin II-associated	8	8	1	1	1	1	1	1	0	1	0	0	1	0	0	0
1156	1660		Conserved hypothetical protein	8	10	1	1	1	1	1	0	1	1	1	0	0	0	1	1
1157	1764		Conserved hypothetical protein	8	15	4	2	1	3	2	0	1	1	0	0	1	0	0	0
1158	1745		Conserved hypothetical membrane protein	8	8	1	1	1	1	0	1	1	1	1	0	0	0	0	0
1159	2479		Conserved hypothetical protein	8	9	1	1	2	1	0	1	1	1	1	0	0	0	0	0
1160	2552		Conserved hypothetical protein	8	9	1	1	1	1	0	1	1	1	1	0	0	0	0	1
1161	1667		Possible sulfotransferase	8	9	1	1	1	1	0	1	1	1	1	0	0	0	0	1
1162	2581		Conserved hypothetical protein	8	8	1	1	1	1	0	1	1	0	1	1	0	0	0	0
1163	1712		Conserved hypothetical protein	8	9	1	1	1	1	0	1	1	0	0	1	1	0	1	0
1164	1787		Conserved hypothetical protein	8	10	1	1	1	1	0	1	1	1	1	0	0	1	1	0
1165	1582		Conserved hypothetical membrane protein	8	10	1	1	1	1	0	1	1	0	1	1	0	0	1	1
1166	1750		Nucleoside-diphosphate-sugar epimerase	8	8	1	1	1	1	0	0	1	1	0	1	1	0	0	0
1167	1590		conserved hypothetical protein distantly related to alpha-glycosyltransferases family 4	8	10	1	1	1	1	0	0	0	1	1	1	1	0	1	1
1168	1695		Uncharacterized protein involved in tolerance to divalent cations	8	11	1	1	1	1	0	0	0	1	1	1	1	1	1	1
1169	3239		Conserved hypothetical protein	8	8	1	1	1	0	1	1	1	1	1	0	0	0	0	0
1170	1858		Conserved hypothetical secreted protein	8	8	1	1	1	0	1	1	1	1	1	0	0	0	0	0
1171	2015		Conserved hypothetical secreted protein	8	8	1	1	1	0	1	1	1	1	1	0	0	0	0	0
1172	1746		Possible alpha-glycosyltransferase, family 4	8	9	1	1	1	0	1	1	1	1	1	0	0	0	0	1
1173	1747		Possible alpha-glycosyltransferase, family 4	8	9	1	1	1	0	1	1	1	1	1	0	0	0	0	1
1174	46		Conserved hypothetical protein	8	26	4	2	1	0	3	1	1	5	0	0	2	0	0	7
1175	1595		Conserved hypothetical protein	8	11	1	1	2	0	1	1	1	1	0	0	1	1	0	1
1176	2003		Conserved hypothetical protein	8	10	1	1	1	0	1	1	1	1	0	0	2	1	0	0
1177	1853	sodC	Superoxide dismutase [Cu-Zn]	8	8	1	1	1	0	1	1	1	1	0	0	1	0	0	0
1178	1204		Ferritin	8	17	1	1	1	0	5	0	1	1	2	1	0	1	1	2
1179	1390		Possible Fe regulatory crp	8	12	1	1	1	0	1	0	1	1	1	2	0	1	1	1
1180	2520		Conserved hypothetical protein	8	9	1	1	1	0	0	1	0	1	1	1	1	0	1	0
1181	2299		Conserved hypothetical protein	8	9	1	1	0	1	1	1	1	1	1	0	0	0	0	1
1182	1703		Possible metal-binding protein	8	10	1	1	0	2	1	1	1	1	1	0	0	1	0	0
1183	1857		Possible nuclease	8	8	1	1	0	1	1	1	1	1	1	0	0	0	0	0
1184	1698		Conserved hypothetical membrane protein	8	10	1	1	0	1	1	2	1	1	0	0	2	0	0	0
1185	1781		Chalcone synthase	8	9	1	1	0	1	1	1	1	0	1	1	0	0	0	1
1186	1780		Possible dehydrogenase	8	9	1	1	0	1	1	1	1	0	1	1	0	0	0	1
1187	1779		SAM-dependent methyltransferase	8	9	1	1	0	1	1	1	1	0	1	1	0	0	0	1
1188	1725		Conserved hypothetical protein	8	11	1	1	0	1	1	0	1	1	0	1	1	1	1	1
1189	2574		Conserved hypothetical protein	8	8	1	1	0	0	1	1	1	1	1	1	0	0	0	0
1190	2871		Conserved hypothetical protein	8	8	1	1	0	0	1	1	1	1	1	1	0	0	0	0
1191	8123		Possible ATP-dependent RNA helicase	8	8	1	1	0	0	1	1	1	1	1	1	0	0	0	0
1192	1743		Possible Na+/H+ antiporter, CPA1 family	8	9	1	1	0	0	1	1	1	1	1	1	0	0	0	1
1193	1871		Conserved hypothetical protein	8	8	1	1	0	0	1	1	1	1	0	1	1	0	0	0
1194	1832		Conserved hypothetical protein	8	8	1	1	0	0	1	1	1	0	1	1	1	0	0	0
1195	1769		Cyclic nucleotide-binding domain	8	8	1	0	1	1	0	1	1	1	1	1	0	0	0	0
1196	1957		Conserved hypothetical protein	8	9	1	0	0	0	1	1	1	1	1	1	1	0	0	1
1197	1958		Conserved hypothetical secreted protein	8	9	1	0	0	0	1	1	1	1	1	1	1	0	0	1
1198	1822	asnB	Asparagine synthase (glutamine-hydrolyzing)	8	10	0	1	1	1	1	0	1	1	1	0	2	0	1	0
1199	1533		similar to N-terminal part of GDP-fucose synthetase	8	12	0	1	0	1	1	2	1	1	0	2	1	1	1	0
1200	8122		Bifunctional family 51 beta-glycosyltransferase/PBP transpeptidase (murein polymerase)	8	9	0	1	0	0	1	1	1	1	1	2	1	0	0	0
1201	2819		Conserved hypothetical protein	8	8	0	1	0	0	1	1	1	1	1	1	1	0	0	0
1202	1740		Conserved hypothetical protein	8	10	0	0	2	1	1	1	1	1	1	2	0	0	0	0
1203	1818		Possible serine/threonine specific protein phosphatase	8	9	0	0	1	1	1	1	1	1	1	1	0	0	0	1
1204	1532	arsR	arsenical resistance operon repressor	8	11	0	0	1	1	0	1	1	1	1	1	1	1	1	1
1205	1739		Conserved hypothetical protein	8	9	0	0	1	1	0	1	1	1	1	1	1	0	0	1
1206	1823		Conserved hypothetical protein	8	8	0	0	1	1	0	1	1	1	1	1	1	0	0	0
1207	2200		Conserved hypothetical protein	8	8	0	0	1	1	0	1	1	1	1	1	1	0	0	0
1208	1817		Conserved hypothetical membrane protein	8	9	0	0	1	0	1	1	1	1	1	1	1	0	0	1
1209	1825		Conserved hypothetical protein	8	9	0	0	1	0	1	1	1	1	2	1	1	0	0	0
1210	2207		Conserved hypothetical protein	8	8	0	0	1	0	1	1	1	1	1	1	1	0	0	0
1211	1894		L-alanine-DL-glutamate epimerase	8	8	0	0	1	0	1	1	1	1	1	1	1	0	0	0
1212	1655		Possible sugar kinase	8	11	0	0	1	0	1	1	1	1	1	1	1	1	1	1
1213	1816		Tfp pilus assembly protein, pilus retraction ATPase PilT	8	9	0	0	1	0	1	1	1	1	1	1	1	0	0	1
1214	1164		Possible phosphatase	8	14	0	0	0	2	1	2	2	1	2	2	2	0	0	0
1215	1820		Universal stress protein family protein	8	9	0	0	0	1	1	1	1	1	1	1	1	0	0	1
1216	1718		Conserved hypothetical membrane protein	9	10	1	1	1	1	1	1	1	1	1	0	0	1	0	0
1217	1922		Conserved hypothetical membrane protein	9	9	1	1	1	1	1	1	1	1	1	0	0	0	0	0
1218	79		Conserved hypothetical protein	9	19	2	1	6	2	3	1	2	1	1	0	0	0	0	0
1219	1476		Conserved hypothetical protein	9	12	1	1	1	1	1	1	1	1	1	0	0	1	1	1

Line No.	Cluster No. in cyanorak	Gene Name	Product	No. of *Synechococcus* strains per cluster	No. of sequ. per cluster (14 genomes)	Synechococcus											Prochlorococcus		
						Subcluster 5.1A					Subcluster 5.1B				5.2	5.3	HL	LL	
						BL107	CC9902	CC9605	WH8102	CC9311	WH7803	WH7805	RS9916	RS9917	WH5701	RCC307	MED4	SS120	MIT9313
1220	1495		Conserved hypothetical protein	9	12	1	1	1	1	1	1	1	1	1	0	0	1	1	1
1221	1666		Conserved hypothetical protein	9	10	1	1	1	1	1	1	1	1	1	0	0	0	0	1
1222	1804		Conserved hypothetical protein	9	10	1	1	1	1	1	1	1	1	1	0	0	0	0	1
1223	1810		Conserved hypothetical protein	9	9	1	1	1	1	1	1	1	1	1	0	0	0	0	0
1224	1953		Conserved hypothetical protein	9	10	1	1	1	1	1	1	1	1	1	0	0	0	0	1
1225	2131		Conserved hypothetical protein	9	12	1	1	1	1	3	1	1	1	1	0	0	0	1	0
1226	2136		Conserved hypothetical protein	9	9	1	1	1	1	1	1	1	1	1	0	0	0	0	0
1227	2560		Conserved hypothetical protein	9	10	1	1	1	1	1	1	1	1	1	0	0	0	1	0
1228	9063		Conserved hypothetical protein	9	12	1	1	1	1	1	1	1	1	1	0	0	1	1	1
1229	1543		Conserved hypothetical protein distantly related to glycosyltransferases, family 4	9	11	1	1	1	1	1	1	1	1	1	0	0	0	1	1
1230	1723		Conserved hypothetical secreted protein	9	10	1	1	1	1	1	1	1	1	1	0	0	0	0	1
1231	1784		Conserved hypothetical secreted protein	9	9	1	1	1	1	1	1	1	1	1	0	0	0	0	0
1232	1509		Cysteine synthase	9	12	1	1	1	1	1	1	1	1	1	0	0	1	1	1
1233	1286		FAD/FMN-containing dehydrogenase	9	13	1	1	1	1	1	1	2	1	1	0	0	1	1	1
1234	1508		Cystathionine beta-lyase/cystathioninegamma-synthase	9	12	1	1	1	1	1	1	1	1	1	0	0	1	1	1
1235	8120		peptidoglycan lytic transglycosylase	9	10	1	1	1	1	1	1	1	1	1	0	0	0	0	1
1236	1753		Possible L-asparaginase	9	9	1	1	1	1	1	1	1	1	1	0	0	0	0	0
1237	1775		Possible Na+/H+ antiporter, CPA2 family	9	9	1	1	1	1	1	1	1	1	1	0	0	0	0	0
1238	1788		Possible peptidase (U32 family)	9	9	1	1	1	1	1	1	1	1	1	0	0	0	0	0
1239	1688		Ribonuclease PH	9	10	1	1	1	1	1	1	1	1	1	0	0	0	0	1
1240	1708		Beta-glycosyltransferase, family 2	9	10	1	1	1	1	1	1	1	1	0	1	0	0	0	1
1241	1558		Conserved hypothetical membrane protein	9	11	1	1	1	1	1	1	1	1	0	1	0	1	0	1
1242	1811		Conserved hypothetical protein	9	9	1	1	1	1	1	1	1	1	0	1	0	0	0	0
1243	2132		Conserved hypothetical protein with a signal peptdie	9	9	1	1	1	1	1	1	1	1	0	1	0	0	0	0
1244	1556		Possible multidrug efflux ABC transporter	9	11	1	1	1	1	1	1	1	1	0	1	0	1	0	1
1245	1557		Possible multidrug efflux ABC transporter	9	11	1	1	1	1	1	1	1	1	0	1	0	1	0	1
1246	1395	pebA	15,16 dihydrobiliverdin:ferredoxin oxidoreductase	9	12	1	1	1	1	1	1	1	1	0	0	1	1	1	1
1247	1487		AEC transporter family protein	9	12	1	1	1	1	1	1	1	1	0	0	1	1	1	1
1248	1855		Conserved hypothetical membrane protein	9	9	1	1	1	1	1	1	1	1	0	0	1	0	0	0
1249	2295		Conserved hypothetical membrane protein	9	9	1	1	1	1	1	1	1	1	0	0	1	0	0	0
1250	1433		Conserved hypothetical protein	9	12	1	1	1	1	1	1	1	1	0	0	1	1	1	1
1251	1445		Conserved hypothetical protein	9	12	1	1	1	1	1	1	1	1	0	0	1	1	1	1
1252	1715		Conserved hypothetical protein	9	12	1	1	1	1	1	1	1	1	0	0	1	1	1	1
1253	1727		Conserved hypothetical protein	9	10	1	1	1	1	1	1	1	1	0	0	1	1	0	0
1254	1782		Conserved hypothetical protein	9	9	1	1	1	1	1	1	1	1	0	0	1	0	0	0
1255	1809		Conserved hypothetical protein	9	11	1	1	1	1	1	1	1	1	0	0	1	0	1	1
1256	1761	unk5	Conserved hypothetical protein, gene in phycobilisome rod gene region	9	9	1	1	1	1	1	1	1	1	0	0	1	0	0	0
1257	1762	unk4	Conserved hypothetical protein, gene in phycobilisome rod gene region	9	9	1	1	1	1	1	1	1	1	0	0	1	0	0	0
1258	1758	unk12	conserved hypothetical protein, gene in phycobilisome rod gene region	9	9	1	1	1	1	1	1	1	1	0	0	1	0	0	0
1259	1834	unk13	conserved hypothetical protein, gene in phycobilisome rod gene region	9	11	1	1	1	1	1	1	1	1	0	0	1	0	1	1
1260	1989		Conserved hypothetical secreted protein	9	9	1	1	1	1	1	1	1	1	0	0	1	0	0	0
1261	69	cpeA	C-phycoerythrin class I alpha subunit	9	9	1	1	1	1	1	1	1	1	0	0	1	0	0	0
1262	8004	cpeB	C-phycoerythrin class I, beta subunit	9	9	1	1	1	1	1	1	1	1	0	0	1	0	0	0
1263	1676	ntrP	Nitrate/nitrite transporter, MFS family	9	10	1	1	1	2	1	1	1	1	0	0	1	0	0	0
1264	1760	cpeC	phycobilisome rod linker polypeptide (Lr), C-phycoerythrin class I-associated (CpeC)	9	9	1	1	1	1	1	1	1	1	0	0	1	0	0	0
1265	8020	cpeE	phycobilisome rod linker polypeptide (Lr), C-phycoerythrin class I-associated (CpeE)	9	9	1	1	1	1	1	1	1	1	0	0	1	0	0	0
1266	1759	cpeR	phycoerythrin operon regulator	9	9	1	1	1	1	1	1	1	1	0	0	1	0	0	0
1267	1394	pebB	phycoerythrobilin:ferredoxin oxidoreductase	9	12	1	1	1	1	1	1	1	1	0	0	1	1	1	1
1268	1397	cpeS	Possible phycoerythrobilin:phycoerythrin lyase CpeS	9	12	1	1	1	1	1	1	1	1	0	0	1	1	1	1
1269	1572	cpeT	Possible phycoerythrobilin:phycoerythrin lyase CpeT	9	11	1	1	1	1	1	1	1	1	0	0	1	0	1	1
1270	1757	cpeU	Possible phycoerythrobilin:phycoerythrin lyase CpeU	9	9	1	1	1	1	1	1	1	1	0	0	1	0	0	0
1271	1570	cpeY	Possible phycoerythrobilin:phycoerythrin lyase CpeY	9	11	1	1	1	1	1	1	1	1	0	0	1	0	1	1
1272	1571	cpeZ	Possible phycoerythrobilin:phycoerythrin lyase CpeZ	9	11	1	1	1	1	1	1	1	1	0	0	1	0	1	1
1273	8022	mpeD	rod linker polypeptide (Lr), C-phycoerythrin I and II-associated	9	9	1	1	1	1	1	1	1	1	0	0	1	0	0	0
1274	1517		sodium:solute symporter family, possibly glucose transporter	9	11	1	1	1	1	1	1	1	1	0	0	1	0	1	1
1275	2147		Zn-ribbon protein	9	10	1	1	1	1	1	1	1	0	1	1	0	0	1	0
1276	1902		Conserved hypothetical protein	9	9	1	1	1	1	1	1	1	0	1	0	1	0	0	0
1277	1796		Conserved hypothetical protein	9	9	1	1	1	1	1	1	1	0	0	1	1	0	0	0
1278	1812		Conserved hypothetical protein	9	16	1	2	1	1	2	1	0	2	0	1	1	1	1	2
1279	1405		Acetyltransferase	9	12	1	1	1	1	1	0	1	1	1	1	0	1	1	1
1280	1235		GAF domain protein	9	13	1	1	1	2	1	0	1	1	0	3	1	1	0	0
1281	1661		Conserved hypothetical membrane protein	9	10	1	1	1	1	1	0	1	1	0	1	1	0	1	0
1282	1200		Conserved hypothetical protein	9	13	1	1	1	1	1	0	1	1	1	0	1	0	3	1
1283	1682		Rubrerythrin	9	10	1	1	1	1	1	0	0	1	1	1	1	0	0	1
1284	1356		Carbonic anhydrase	9	12	1	1	2	1	1	0	0	1	2	2	1	0	0	0
1285	1800		Glutathione S-transferase	9	10	1	1	1	1	2	0	0	1	1	1	1	0	0	0
1286	2019		Periplasmic protein involved in polysaccharide export	9	12	1	1	1	2	1	0	0	1	1	1	1	1	1	0
1287	1335		beta-glycosyltransferase, family 2	9	12	1	1	1	1	0	1	1	1	1	0	1	1	1	1
1288	1222		Conserved hypothetical protein	9	12	1	1	1	1	0	1	1	1	1	0	1	1	1	1
1289	1770		Conserved hypothetical protein	9	9	1	1	1	1	0	1	1	1	1	0	1	0	0	0
1290	1169		Conserved hypothetical membrane protein	9	12	1	1	1	2	0	1	1	1	0	1	2	0	0	1
1291	1603		Conserved hypothetical membrane protein	9	10	1	1	1	1	0	1	1	1	0	1	1	1	0	0
1292	1847		Conserved hypothetical protein	9	10	1	1	1	1	0	1	1	1	0	1	1	1	0	0
1293	1625	ggt	Gamma-glutamyltranspeptidase	9	10	1	1	1	1	0	1	1	1	0	1	1	0	1	0
1294	1544		Conserved hypothetical membrane protein	9	10	1	1	1	1	0	1	1	1	1	0	1	0	0	1
1295	2178		Conserved hypothetical protein	9	10	1	1	1	1	0	1	1	1	1	1	0	0	0	1
1296	1771	clc3	Possible chloride channel	9	9	1	1	1	1	0	1	1	0	1	1	1	0	0	0
1297	1679		3-hydroxyisobutyrate dehydrogenase related protein	9	9	1	1	1	1	0	1	1	1	1	0	1	0	0	0
1298	1372		conserved hypothetical protein	9	12	1	1	1	0	1	2	3	1	1	1	0	0	0	0
1299	1669		FNT formate/nitrate transporter family protein	9	10	1	1	1	0	1	1	1	1	1	0	1	0	0	1
1300	1680		Conserved hypothetical protein	9	12	1	1	1	0	1	1	1	1	1	0	1	1	1	1
1301	1850		Conserved hypothetical protein	9	10	1	1	1	0	1	1	1	1	0	1	1	0	1	0
1302	1912		Conserved hypothetical secreted protein	9	11	1	1	1	0	1	1	1	1	0	1	1	1	1	0
1303	1702		Hydantoinase B/oxoprolinase	9	10	1	1	1	0	1	1	1	1	0	1	1	0	0	1
1304	1696		PucC homolog, major facilitator superfamily	9	11	1	1	1	0	2	1	1	0	1	1	1	0	1	0
1305	1859		Conserved hypothetical protein	9	10	1	1	1	0	1	1	1	0	1	1	1	0	1	0
1306	1862		Conserved hypothetical protein	9	9	1	1	1	0	1	1	0	1	1	1	1	0	0	0
1307	2290		Conserved hypothetical protein	9	9	1	1	1	0	1	1	1	1	1	1	0	0	0	0
1308	2052		Conserved hypothetical protein	9	10	1	1	1	0	0	1	2	1	1	1	1	0	0	0
1309	1343		Fatty acid desaturase	9	12	1	1	0	1	1	1	1	0	1	1	1	1	1	1
1310	21		Possible pyridoxal phosphate-dependent enzyme	9	17	3	1	0	1	1	0	1	1	2	1	1	3	1	1
1311	2282		Possible transcriptional regulator	9	9	1	1	0	1	1	1	1	1	0	1	1	0	0	0
1312	1790		Carotenoid binding protein/orange carotenoid protein (OCP)	9	9	1	1	0	1	1	1	1	0	1	1	1	0	0	0
1313	1608		Conserved hypothetical protein	9	11	1	1	0	2	1	1	1	0	1	1	1	1	0	0
1314	1792		Conserved hypothetical protein	9	9	1	1	0	1	1	1	1	0	1	1	1	0	0	0
1315	1791		Possible beta-carotene ketolase	9	9	1	1	0	1	1	1	1	0	1	1	1	0	0	0
1316	1722		Possible ligand gated channel (GIC family)	9	10	1	1	0	1	1	1	1	0	1	1	1	0	0	1
1317	1801		Conserved hypothetical protein	9	9	1	1	0	1	1	0	1	1	1	1	1	0	0	0
1318	2016		Conserved hypothetical protein	9	10	1	1	0	0	1	1	1	1	1	1	1	0	1	0
1319	2471		Conserved hypothetical protein	9	11	1	1	0	0	1	1	1	1	1	1	1	0	1	1
1320	2193		Conserved hypothetical secreted protein	9	10	1	1	0	0	1	1	1	1	1	2	1	0	0	0
1321	8117		Fatty-acid desaturase	9	12	1	1	0	0	1	1	1	1	1	1	1	1	1	1
1322	1621		Possible redox protein	9	11	1	1	0	0	1	1	1	1	1	1	1	1	0	1
1323	1744		SAM dependent carboxyl methyltransferase	9	9	1	0	1	1	1	1	1	1	1	0	1	0	0	0
1324	8093	gap3	Glyceraldehyde-3-phosphate dehydrogenase	9	12	1	0	1	1	0	1	1	1	1	1	1	1	1	1
1325	1248		multidrug efflux transporter, MFS family	9	12	1	0	1	1	0	1	1	1	1	1	1	1	1	1
1326	1606	ptrA	Potential phosphate regulator, Crp family transcriptional regulatory protein	9	11	1	0	1	1	0	1	1	1	1	1	1	1	1	0
1327	8015	phoB	Two-component system response regulator, phosphate sensing	9	11	1	0	1	1	0	1	1	1	1	1	1	1	0	1
1328	1687		Type II secretory pathway, ATPase PulE/Tfp pilus assembly pathway, ATPase PilB	9	10	1	0	0	1	1	1	1	1	1	1	1	0	0	1
1329	1685		Type II secretory pathway, component PulD	9	10	1	0	0	1	1	1	1	1	1	1	1	0	0	1
1330	1720		Type II secretory pathway, prepilin signal peptidase PulO	9	11	1	0	0	1	1	1	1	1	1	1	1	0	1	1

Line No.	Cluster No. in cyanorak	Gene Name	Product	No. of *Synechococcus* strains per cluster	No. of sequ. per cluster (14 genomes)	Synechococcus Subcluster 5.1A					Synechococcus Subcluster 5.1B				Synechococcus 5.2	Synechococcus 5.3	Prochlorococcus HL		Prochlorococcus LL
						BL107	CC9902	CC9605	WH8102	CC9311	WH7803	WH7805	RS9916	RS9917	WH5701	RCC307	MED4	SS120	MIT9313
1331	1686		Pentapeptide and Tetratricopeptide repeat-containing protein	9	10	1	0	0	1	1	1	1	1	1	1	1	0	0	1
1332	2		Two-component system response regulator	9	11	0	1	1	1	2	1	1	1	1	0	1	0	0	1
1333	1161	gmD	GDP-mannose 4,6-dehydratase	9	12	0	1	0	1	1	1	1	1	1	2	1	1	1	0
1334	1898		Conserved hypothetical membrane protein	9	9	0	0	1	1	1	1	1	1	1	1	1	0	0	0
1335	1899		Conserved hypothetical membrane protein	9	9	0	0	1	1	1	1	1	1	1	1	1	0	0	0
1336	9126		Conserved hypothetical membrane protein	9	10	0	0	1	1	1	1	1	1	1	1	1	0	0	1
1337	190		Conserved hypothetical protein	9	23	0	0	1	1	5	2	2	4	3	2	1	0	0	2
1338	1738		Conserved hypothetical protein	9	10	0	0	1	1	1	1	1	1	1	1	1	0	0	1
1339	2048		Conserved hypothetical protein	9	10	0	0	1	1	1	1	1	1	1	1	1	0	0	1
1340	1163		Conserved hypothetical secreted protein	9	14	0	0	1	1	1	2	1	1	2	3	1	0	0	1
1341	8118		Cytochrome c oxidase subunit I	9	11	0	0	1	1	1	1	1	1	1	2	2	0	0	0
1342	1900		Cytochrome c oxidase subunit II	9	9	0	0	1	1	1	1	1	1	1	1	1	0	0	0
1343	1827		Cytochrome c oxidase subunit III	9	9	0	0	1	1	1	1	1	1	1	1	1	0	0	0
1344	1735		Fe-S oxidoreductase	9	10	0	0	1	1	1	1	1	1	1	1	1	0	0	1
1345	1737	envZ	HKIII HAMP, chk91, Possible osmosensory histidine kinase	9	10	0	0	1	1	1	1	1	1	1	1	1	0	0	1
1346	1536		Possible flavoprotein related to choline dehydrogenase	9	13	0	0	1	1	2	2	1	1	2	1	2	0	0	0
1347	1657		Possible membrane-bound transcriptional regulator	9	11	0	0	1	1	1	1	1	1	1	2	1	0	0	1
1348	1658		Possible phosphohistidine phosphatase	9	11	0	0	1	1	1	1	1	1	1	1	1	0	0	2
1349	1732		Possible Pirin-related protein	9	10	0	0	1	1	1	1	1	1	1	1	1	1	0	0
1350	1828		Possible polysaccharide-forming beta-glycosyltransferase, family 2	9	9	0	0	1	1	1	1	1	1	1	1	1	0	0	0
1351	8017		RRII, OmpR, crr72	9	10	0	0	1	1	1	1	1	1	1	1	1	0	0	1
1352	1531	phoR	Two-component system sensor histidine kinase, phosphate sensing PhoR	9	12	0	0	1	1	0	1	1	1	1	1	1	1	0	3
1353	34	groL2	60 kDa chaperonin 2 (Protein Cpn60 2) (GroEL protein 2)	10	13	1	1	1	1	1	1	1	1	1	1	0	1	1	1
1354	8060		ABC transporter for amino acids, ATP binding component	10	12	1	1	1	1	1	1	1	1	1	1	0	0	1	1
1355	1309		ABC transporter, substrate binding protein	10	13	1	1	1	2	1	1	2	1	1	1	0	0	0	1
1356	1600		ABC transporter, substrate binding protein, possibly Mn	10	13	1	1	1	1	1	1	1	1	1	1	0	1	1	1
1357	22		ABC-type Mn2+/Zn2+ transport system ATPase component	10	13	1	1	1	1	1	1	1	1	1	1	0	1	1	1
1358	1244		ABC-type Mn2+/Zn2+ transport system permease component	10	13	1	1	1	1	1	1	1	1	1	1	0	1	1	1
1359	1587		CbbX protein homolog	10	11	1	1	1	1	1	1	1	1	1	1	0	0	0	1
1360	1652		Conserved hypothetical membrane protein	10	13	1	1	1	1	1	1	1	1	1	1	0	1	1	1
1361	1189		Conserved hypothetical membrane protein	10	13	1	1	1	1	1	1	1	1	1	1	0	1	1	1
1362	1217		Conserved hypothetical membrane protein	10	13	1	1	1	2	1	1	1	1	1	2	0	0	0	1
1363	1602		Conserved hypothetical membrane protein	10	11	1	1	1	1	1	1	1	1	1	1	0	0	0	1
1364	1684		Conserved hypothetical membrane protein	10	10	1	1	1	1	1	1	1	1	1	1	0	0	0	0
1365	1991		Conserved hypothetical membrane protein	10	10	1	1	1	1	1	1	1	1	1	1	0	0	0	0
1366	99		Conserved hypothetical protein	10	11	1	1	1	1	1	1	1	1	1	1	0	0	0	1
1367	1275		Conserved hypothetical protein	10	13	1	1	1	1	1	1	1	1	1	1	0	1	1	1
1368	1283		Conserved hypothetical protein	10	13	1	1	1	1	1	1	1	1	1	1	0	1	1	1
1369	1348		Conserved hypothetical protein	10	12	1	1	1	1	1	1	1	1	1	1	0	0	1	1
1370	1419		Conserved hypothetical protein	10	12	1	1	1	1	1	1	1	1	1	1	0	0	1	1
1371	1432		Conserved hypothetical protein	10	13	1	1	1	1	1	1	1	1	1	1	0	1	1	1
1372	1478		Conserved hypothetical protein	10	13	1	1	1	1	1	1	1	1	1	1	0	1	1	1
1373	1491		Conserved hypothetical protein	10	12	1	1	1	1	1	1	1	1	1	2	0	0	0	1
1374	1562		Conserved hypothetical protein	10	11	1	1	1	1	1	1	1	1	1	1	0	0	0	1
1375	1617		Conserved hypothetical protein	10	11	1	1	1	1	1	1	1	1	1	1	0	0	0	1
1376	1618		Conserved hypothetical protein	10	14	1	1	1	1	1	1	1	1	1	1	0	1	1	2
1377	1664		Conserved hypothetical protein	10	11	1	1	1	1	1	1	1	1	1	1	0	0	0	1
1378	1700		Conserved hypothetical protein	10	10	1	1	1	1	1	1	1	1	1	1	0	0	0	0
1379	1713		Conserved hypothetical protein	10	10	1	1	1	1	1	1	1	1	1	1	0	0	0	0
1380	1717		Conserved hypothetical protein	10	10	1	1	1	1	1	1	1	1	1	1	0	0	0	0
1381	1719		Conserved hypothetical protein	10	11	1	1	1	1	1	1	1	1	1	1	0	0	1	0
1382	1783		Conserved hypothetical protein	10	10	1	1	1	1	1	1	1	1	1	1	0	0	0	0
1383	1849		Conserved hypothetical protein	10	10	1	1	1	1	1	1	1	1	1	1	0	0	0	0
1384	2142		Conserved hypothetical protein	10	10	1	1	1	1	1	1	1	1	1	1	0	0	0	0
1385	2352		Conserved hypothetical protein	10	13	1	1	1	1	1	1	2	1	1	1	0	0	1	1
1386	2389		Conserved hypothetical protein	10	11	1	1	1	1	2	1	1	1	1	1	0	0	0	0
1387	4357		conserved hypothetical protein	10	11	1	1	1	1	1	1	1	1	1	1	0	0	0	1
1388	1577		Conserved hypothetical secreted protein	10	11	1	1	1	1	1	1	1	1	1	1	0	0	0	1
1389	8104		Cysteine synthase	10	13	1	1	1	1	1	1	1	1	1	1	0	1	1	1
1390	1238		Cytosine deaminase	10	13	1	1	1	1	1	1	1	1	1	1	0	1	1	1
1391	1710		dnaJ domain-containing protein	10	10	1	1	1	1	1	1	1	1	1	1	0	0	0	0
1392	1299		Ferredoxin	10	13	1	1	1	1	1	1	1	1	1	1	0	1	1	1
1393	1651		GTP-binding protein; HflX	10	11	1	1	1	1	1	1	1	1	1	1	0	0	0	1
1394	1615		HIT family hydrolase protein	10	11	1	1	1	1	1	1	1	1	1	1	0	0	0	1
1395	1265		Light-dependent protochlorophyllide oxido-reductase	10	13	1	1	1	1	1	1	1	1	1	1	0	1	1	1
1396	1274	petE	Plastocyanin precursor	10	13	1	1	1	1	1	1	1	1	1	1	0	1	1	1
1397	1488		Possible ABC amino acid transporter, membrane protein	10	12	1	1	1	1	1	1	1	1	1	1	0	0	1	1
1398	1624		Possible ABC amino acid transporter, membrane protein	10	12	1	1	1	1	1	1	1	1	1	1	0	0	1	1
1399	1489		Possible ABC amino acid transporter, substrate binding protein	10	12	1	1	1	1	1	1	1	1	1	1	0	0	1	1
1400	8039		Possible ABC multidrug efflux transporter	10	15	1	1	1	1	1	1	1	1	1	3	0	1	1	1
1401	1542		Possible alpha-glycosyltransferase, family 4	10	11	1	1	1	1	1	1	1	1	1	1	0	0	0	1
1402	1227	arsC	Possible arsenate reductase	10	13	1	1	1	1	1	1	1	1	1	1	0	1	1	1
1403	1635		Possible arylsulfatase regulatory protein	10	11	1	1	1	1	1	1	1	1	1	1	0	0	0	1
1404	379	clc1	Possible chloride channel, ClC family	10	14	1	1	1	1	1	1	1	1	1	2	0	1	1	1
1405	1785		Possible type 3 alternative RNA polymerase sigma factor	10	10	1	1	1	1	1	1	1	1	1	1	0	0	0	0
1406	1604		Possible zeta-carotene desaturase	10	11	1	1	1	1	1	1	1	1	1	1	0	0	0	1
1407	1716		Rieske-type iron-sulfur cluster-binding protein	10	10	1	1	1	1	1	1	1	1	1	1	0	0	0	0
1408	1637		RRII OmpR	10	11	1	1	1	1	1	1	1	1	1	1	0	0	0	1
1409	1272		Short-chain dehydrogenase/reductase family enzyme	10	13	1	1	1	1	1	1	1	1	1	1	0	1	1	1
1410	2117		Transcriptional regulator, luxR family	10	11	1	1	1	1	1	1	1	1	1	1	0	0	0	1
1411	1177		Uncharacterized AbrB family conserved membrane protein	10	13	1	1	1	1	1	1	1	1	1	1	0	1	1	1
1412	1813	ppt	4 prime-phosphopantetheinyl transferase related protein	10	13	1	1	1	1	1	1	1	1	1	0	1	1	1	1
1413	58		Conserved hypothetical membrane protein	10	11	1	1	1	1	1	1	1	1	1	0	1	0	0	1
1414	1616		Conserved hypothetical membrane protein	10	11	1	1	1	1	1	1	1	1	1	0	1	0	0	1
1415	1711		Conserved hypothetical membrane protein	10	11	1	1	1	1	1	1	1	1	1	0	1	0	1	0
1416	141		Conserved hypothetical protein	10	15	2	2	1	2	1	1	1	1	1	0	1	2	0	0
1417	1203		Conserved hypothetical protein	10	13	1	1	1	1	1	1	1	1	1	0	1	1	1	1
1418	1218		Conserved hypothetical protein	10	17	1	1	3	1	3	1	1	1	1	0	1	1	1	1
1419	1271		Conserved hypothetical protein	10	13	1	1	1	1	1	1	1	1	1	0	1	1	1	1
1420	1300		Conserved hypothetical protein	10	13	1	1	1	1	1	1	1	1	1	0	1	1	1	1
1421	1442		Conserved hypothetical protein	10	12	1	1	1	1	1	1	1	1	1	0	1	1	1	0
1422	1473		Conserved hypothetical protein	10	13	1	1	1	1	1	1	1	1	1	0	1	1	1	1
1423	1483		Conserved hypothetical protein	10	14	1	1	1	1	2	1	2	1	1	0	1	1	1	0
1424	1523		Conserved hypothetical protein	10	12	1	1	1	1	1	1	1	1	1	0	1	0	1	1
1425	1553		Conserved hypothetical protein	10	11	1	1	1	1	1	1	1	1	1	0	1	0	0	1
1426	1559		Conserved hypothetical protein	10	12	1	1	1	1	1	1	1	1	1	0	1	0	1	1
1427	1589		Conserved hypothetical protein	10	11	1	1	1	1	1	1	1	1	1	0	1	0	0	1
1428	1601		Conserved hypothetical protein	10	11	1	1	1	1	1	1	1	1	1	0	1	0	0	1
1429	1642		Conserved hypothetical protein	10	11	1	1	1	1	1	1	1	1	1	0	1	0	0	1
1430	1693		Conserved hypothetical protein	10	11	1	1	1	1	1	1	1	1	1	0	1	0	0	1
1431	1694		Conserved hypothetical protein	10	10	1	1	1	1	1	1	1	1	1	0	1	0	0	0
1432	1706		Conserved hypothetical protein	10	13	1	1	1	1	2	1	1	2	1	0	1	0	0	1
1433	1714		Conserved hypothetical protein	10	10	1	1	1	1	1	1	1	1	1	0	1	0	0	0
1434	1724		Conserved hypothetical protein	10	10	1	1	1	1	1	1	1	1	1	0	1	0	0	0
1435	1805		Conserved hypothetical protein	10	20	1	2	4	2	1	1	1	1	1	0	1	2	1	2
1436	1926		Conserved hypothetical protein	10	10	1	1	1	1	1	1	1	1	1	0	1	0	0	0
1437	2141		Conserved hypothetical protein	10	11	1	1	1	1	1	1	1	2	1	0	1	0	0	0
1438	3334		Conserved hypothetical protein	10	11	1	1	2	1	1	1	1	1	1	0	1	0	0	0
1439	8119		Conserved hypothetical protein	10	11	1	1	1	1	1	1	1	1	1	0	1	0	0	1
1440	85		Conserved hypothetical secreted protein	10	17	1	1	1	2	1	2	2	2	2	0	2	0	0	1
1441	1552		Cyanate lyase	10	11	1	1	1	1	1	1	1	1	1	0	1	1	0	0

Synechococcus accessory genome (minus unique genes)

Line No.	Cluster No. in cyanorak	Gene Name	Product	No. of *Synechococcus* strains per cluster	No. of sequ. per cluster (14 genomes)	Synechococcus											Prochlorococcus		
						Subcluster 5.1A					Subcluster 5.1B				5.2	5.3	HL		LL
						BL107	CC9902	CC9605	WH8102	CC9311	WH7803	WH7805	RS9916	RS9917	WH5701	RCC307	MED4	SS120	MIT9313
1442	26		Cysteine synthase	10	13	1	1	1	1	1	1	1	1	1	0	1	1	1	1
1443	1588	smr	DMT family multidrug efflux pump	10	12	1	1	1	1	1	1	1	1	3	0	1	0	0	0
1444	1370	gpgP	Glucosyl-3-phosphoglycerate phosphatase	10	12	1	1	1	1	1	1	1	1	1	0	1	1	1	0
1445	1368	gpgS	Glucosyl-3-phosphoglycerate synthase	10	12	1	1	1	1	1	1	1	1	1	0	1	1	1	0
1446	1539		Glutamate dehydrogenase/leucine dehydrogenase	10	11	1	1	1	2	1	1	1	1	1	0	1	0	0	0
1447	1697		Histone deacetylase family protein	10	10	1	1	1	1	1	1	1	1	1	0	1	0	0	0
1448	1285		Homoserine O-succinyltransferase	10	13	1	1	1	1	1	1	1	1	1	0	1	1	1	1
1449	1344		Integral membrane protein possibly involved in chromosome condensation	10	12	1	1	1	1	1	1	1	1	1	0	1	0	1	1
1450	1548		Integral membrane protein possibly involved in chromosome condensation	10	13	1	1	1	1	1	1	1	1	1	0	1	1	1	1
1451	1234	mqoA	Malate:quinone oxidoreductase	10	13	1	1	1	1	1	1	1	1	1	0	1	1	1	1
1452	1284		O-Acetyl homoserine sulfhydrylase	10	13	1	1	1	1	1	1	1	1	1	0	1	1	1	1
1453	1187		Possible flavoprotein	10	13	1	1	1	1	1	1	1	1	1	0	1	1	1	1
1454	1663		Possible glycine betaine transporter, BCCT family	10	10	1	1	1	1	1	1	1	1	1	0	1	0	0	0
1455	1690		Possible Pex protein (Period-extender gene product)	10	10	1	1	1	1	1	1	1	1	1	0	1	0	0	0
1456	1224		Possible pterin-4-alpha-carbinolamine dehydratase	10	13	1	1	1	1	1	1	1	1	1	0	1	1	1	1
1457	1503		Possible sodium-dependent transporter, NSS family	10	12	1	1	1	1	1	1	1	1	1	0	1	0	1	1
1458	8100		RNA-binding protein, RRM domain	10	13	1	1	1	1	1	1	1	1	1	0	1	1	1	1
1459	1424		RRII, LuxR	10	13	1	1	1	1	2	2	2	1	1	0	1	0	0	0
1460	1585		SAM-dependent methyltransferase	10	11	1	1	1	1	1	1	1	1	1	0	1	0	0	1
1461	1662		Sarcosine oxidase	10	10	1	1	1	1	1	1	1	1	1	0	1	0	0	0
1462	1564		Secreted protein with pentapeptide repeats	10	11	1	1	1	1	1	1	1	1	1	0	1	0	0	1
1463	1511		S-isoprenylcysteine O-methyltransferase related enzyme	10	13	1	1	1	1	1	1	1	1	1	0	1	1	1	1
1464	964	alsT	Sodium:alanine symporter family protein	10	14	1	1	1	1	1	1	2	1	2	0	1	0	1	1
1465	1369		sucrose phosphorylase	10	12	1	1	1	1	1	1	1	1	1	0	1	1	1	0
1466	45	sulP2	sulfate ion transporter	10	13	1	1	1	1	1	1	1	1	1	0	1	1	1	1
1467	1925		Conserved hypothetical membrane protein	10	11	1	1	2	1	1	1	1	1	0	1	1	0	0	0
1468	62		Conserved hypothetical protein	10	21	3	2	3	3	1	1	1	3	0	1	1	0	0	2
1469	94		Conserved hypothetical protein	10	16	1	2	1	2	1	1	1	1	0	2	2	2	0	0
1470	162		Conserved hypothetical protein	10	16	1	2	2	1	1	1	1	2	0	2	1	0	2	0
1471	1225		Conserved hypothetical protein	10	13	1	1	1	1	1	1	1	1	0	1	1	1	1	1
1472	1674		Conserved hypothetical protein	10	10	1	1	1	1	1	1	1	1	0	1	1	0	0	0
1473	1726		Conserved hypothetical protein	10	10	1	1	1	1	1	1	1	1	0	1	1	0	0	0
1474	1291		Conserved hypothetical secreted protein	10	14	2	2	2	2	1	1	1	1	0	1	1	0	0	0
1475	1376		Hydroxyacid dehydrogenase/reductase family protein	10	12	1	1	1	1	1	1	1	1	0	1	1	0	1	1
1476	1678		Molybdenum cofactor biosynthesis protein A	10	10	1	1	1	1	1	1	1	1	0	1	1	0	0	0
1477	1670		Molybdenum cofactor biosynthesis protein B	10	10	1	1	1	1	1	1	1	1	0	1	1	0	0	0
1478	1673		Molybdenum cofactor biosynthesis protein C	10	10	1	1	1	1	1	1	1	1	0	1	1	0	0	0
1479	1672		Molybdopterin biosynthesis protein moeA	10	10	1	1	1	1	1	1	1	1	0	1	1	0	0	0
1480	1749		Molybdopterin converting factor subunit 1	10	10	1	1	1	1	1	1	1	1	0	1	1	0	0	0
1481	1671		Molybdopterin converting factor subunit 2	10	10	1	1	1	1	1	1	1	1	0	1	1	0	0	0
1482	1677		Molybdopterin-guanine dinucleotide biosynthesis protein A	10	10	1	1	1	1	1	1	1	1	0	1	1	0	0	0
1483	1675	narB	nitrate reductase	10	10	1	1	1	1	1	1	1	1	0	1	1	0	0	0
1484	1619		Possible cytochrome P450	10	11	1	1	1	1	1	1	1	1	0	1	1	0	0	1
1485	1171		Possible thioredoxin	10	13	1	1	1	1	1	1	1	1	0	1	1	1	1	1
1486	1264		Short-chain dehydrogenase/reductase (SDR) superfamily	10	13	1	1	1	1	1	1	1	1	0	1	1	1	1	1
1487	8098		Ferredoxin	10	11	1	1	2	1	1	1	1	0	1	1	1	0	0	0
1488	1576		glycoside hydrolase, family 13	10	11	1	1	1	1	1	1	1	0	1	1	1	0	0	1
1489	8045	bicA	SulP-type bicarbonate transporter	10	10	1	1	1	1	1	1	0	1	1	1	1	0	0	0
1490	1367	urtD	ATP-binding subunit of urea ABC transporter UrtD	10	12	1	1	1	1	1	0	1	1	1	1	1	1	0	1
1491	8074	urtE	ATP-binding subunit of urea ABC transporter UrtE	10	12	1	1	1	1	1	0	1	1	1	1	1	1	0	1
1492	713		Conserved hypothetical protein	10	14	1	1	1	1	1	0	1	1	1	2	1	1	1	1
1493	1555		Conserved hypothetical protein	10	11	1	1	1	1	1	0	1	1	1	1	1	1	0	0
1494	1182		Transglutaminase-like enzyme	10	13	1	1	1	1	1	0	1	1	1	3	1	1	0	0
1495	1365	urtB	Urea ABC transporter, membrane component	10	12	1	1	1	1	1	0	1	1	1	1	1	1	0	1
1496	1366	urtC	Urea ABC transporter, membrane component	10	12	1	1	1	1	1	0	1	1	1	1	1	1	0	1
1497	1361	ureD	Urease accessory protein D	10	12	1	1	1	1	1	0	1	1	1	1	1	1	0	1
1498	1362	ureE	Urease accessory protein E	10	12	1	1	1	1	1	0	1	1	1	1	1	1	0	1
1499	1363	ureF	Urease accessory protein F	10	12	1	1	1	1	1	0	1	1	1	1	1	1	0	1
1500	1364	ureG	Urease accessory protein G	10	12	1	1	1	1	1	0	1	1	1	1	1	1	0	1
1501	1358	ureC	Urease alpha subunit	10	12	1	1	1	1	1	0	1	1	1	1	1	1	0	1
1502	1359	ureB	Urease beta subunit	10	12	1	1	1	1	1	0	1	1	1	1	1	1	0	1
1503	1360	ureC	Urease gamma subunit	10	12	1	1	1	1	1	0	1	1	1	1	1	1	0	1
1504	1173		2-dehydro-3-deoxyphosphooctonate aldolase	10	12	1	1	2	1	0	1	1	1	1	1	1	0	0	1
1505	1172		3-deoxy-manno-octulosonate cytidylyltransferase	10	12	1	1	2	1	0	1	1	1	1	1	1	0	0	1
1506	1323		Conserved hypothetical membrane protein	10	12	1	1	1	1	0	1	1	1	1	1	1	0	1	1
1507	41		Conserved hypothetical protein	10	13	1	1	1	2	0	1	2	1	1	2	1	0	0	0
1508	91		Conserved hypothetical protein	10	14	1	1	2	1	0	1	1	1	1	1	1	0	0	3
1509	215		Conserved hypothetical protein	10	13	1	1	1	1	0	1	1	1	1	1	1	1	1	1
1510	1094		Conserved hypothetical protein	10	13	1	1	1	1	0	1	1	1	1	1	1	1	1	1
1511	1951		Conserved hypothetical protein	10	13	1	1	1	1	0	1	1	2	1	1	1	0	1	1
1512	1305		dTDP-4-dehydrorhamnose 3,5-epimerase	10	12	1	1	1	1	0	1	1	1	1	2	1	0	0	1
1513	1515		dTDP-glucose-4,6-dehydratase	10	11	1	1	1	1	0	1	1	1	1	1	1	0	0	1
1514	1513		glucose-1-phosphate thymidylyltransferase	10	11	1	1	1	1	0	1	1	1	1	1	1	0	0	1
1515	1333		Low specificity phosphatase (HAD superfamily)	10	11	1	1	1	1	0	1	1	1	1	1	1	0	0	1
1516	1443		NUDIX hydrolase	10	11	1	1	1	1	0	1	1	1	1	1	1	0	0	1
1517	1332		Polysialic acid capsule expression protein	10	11	1	1	1	1	0	1	1	1	1	1	1	0	0	1
1518	1514		Possible dTDP-4-dehydrorhamnose reductase	10	11	1	1	1	1	0	1	1	1	1	1	1	0	0	1
1519	1401		Possible enolase-phosphatase E-1	10	11	1	1	1	1	0	1	1	1	1	1	1	0	0	1
1520	1324		Possible inverting glycosyltransferase family 83 utilizing lipid monophospho-sugar donors	10	11	1	1	1	1	0	1	1	1	1	1	1	0	0	1
1521	1402		Sugar aldolase	10	11	1	1	1	1	0	1	1	1	1	1	1	0	0	1
1522	1630		Conserved hypothetical membrane protein	10	11	1	1	1	0	1	1	1	1	1	1	1	0	0	1
1523	1612		Conserved hypothetical protein	10	11	1	1	1	0	1	1	1	1	1	1	1	0	0	1
1524	1627		Conserved hypothetical protein	10	12	1	1	1	0	1	1	1	1	1	1	1	0	1	1
1525	1709		Conserved hypothetical protein	10	12	1	1	1	0	1	1	1	1	1	1	1	0	1	1
1526	1807		Conserved hypothetical protein	10	10	1	1	1	0	1	1	1	1	1	1	1	0	0	0
1527	1945		Conserved hypothetical protein	10	11	1	1	1	0	1	1	1	1	1	1	1	0	0	1
1528	1786		Possible group 3 RNA polymerase sigma factor	10	11	1	1	1	0	1	1	1	1	1	1	1	0	0	1
1529	605		Possible metalloendopeptidase	10	11	1	1	1	0	1	1	1	1	1	1	1	0	0	1
1530	400		Possible small-conductance mechanosensitive channel	10	15	1	1	1	0	2	2	3	1	1	2	1	0	0	0
1531	2164		Conserved hypothetical membrane protein	10	10	1	1	0	1	1	1	1	1	1	1	1	0	0	0
1532	1375		Conserved hypothetical protein	10	12	1	1	0	1	1	1	1	1	1	1	1	0	1	1
1533	2171		Conserved hypothetical protein	10	11	1	1	0	1	1	1	1	1	1	1	1	0	0	1
1534	2535		Conserved hypothetical protein	10	11	1	1	0	1	1	1	1	1	1	1	1	0	0	1
1535	1629		Phosphoglycerate mutase family protein	10	11	1	1	0	1	1	1	1	1	1	1	1	0	0	1
1536	1139		Possible ATPase with chaperone activity	10	14	2	1	0	1	1	1	1	1	1	1	1	0	0	3
1537	1521	ald	Alanine dehydrogenase	10	12	1	0	1	1	1	1	1	1	1	1	1	0	1	1
1538	1165		Inositol monophosphatase family protein	10	13	1	0	1	1	1	1	1	1	1	1	1	1	1	1
1539	1563		Type II secretory pathway, component PulF	10	11	1	0	1	1	1	1	1	1	1	1	1	0	0	1
1540	1969		Conserved hypothetical protein	10	10	0	1	1	1	1	1	1	1	1	1	1	0	0	0
1541	2441		Conserved hypothetical protein	10	10	0	1	1	1	1	1	1	1	1	1	1	0	0	0
1542	1160	petC	Cytochrome b6-f complex iron-sulfur subunit	10	13	0	1	1	1	1	1	1	1	1	1	1	1	1	1

Numbers in red : 1-3 Prochlorococcus strains also contain members of this protein cluster
Green background: genes linked to photosynthesis
