# Supplementary figures and images for: Unraveling the genomic mosaic of a ubiquitous genus of marine cyanobacteria
Source: Genome Biol. 2008 May 28;9(5):R90. doi: 10.1186/gb-2008-9-5-r90 (PMC2441476; doi:10.1186/gb-2008-9-5-r90)

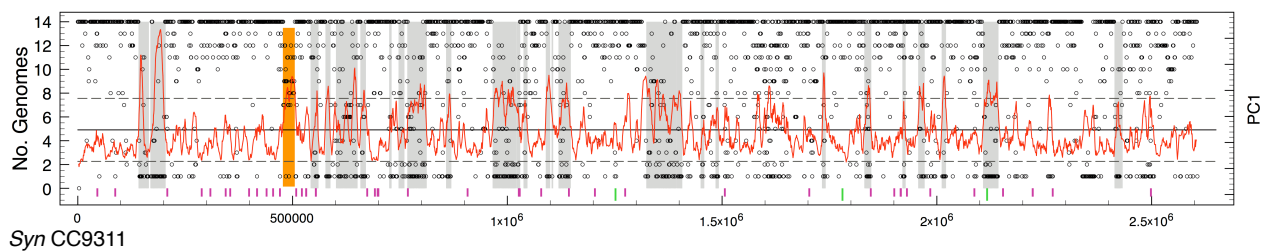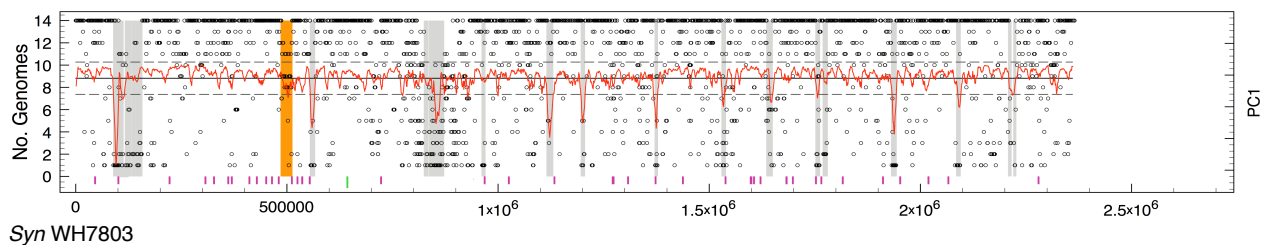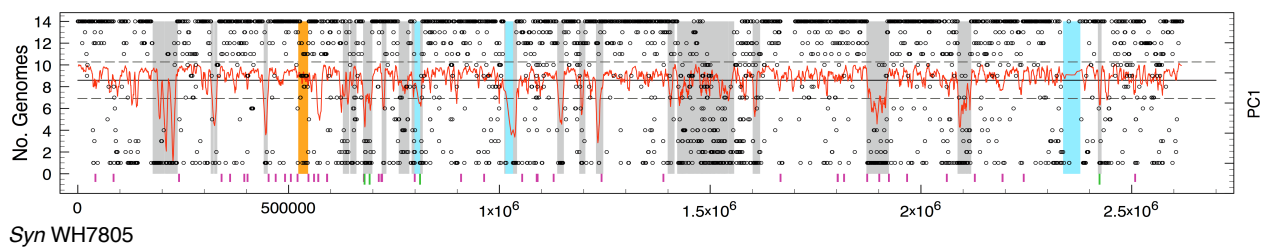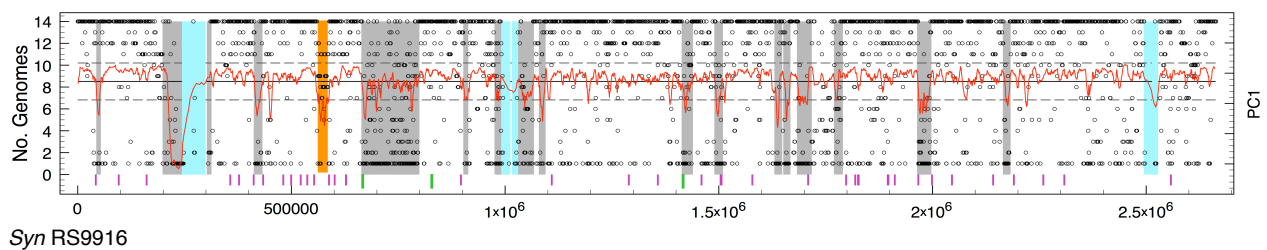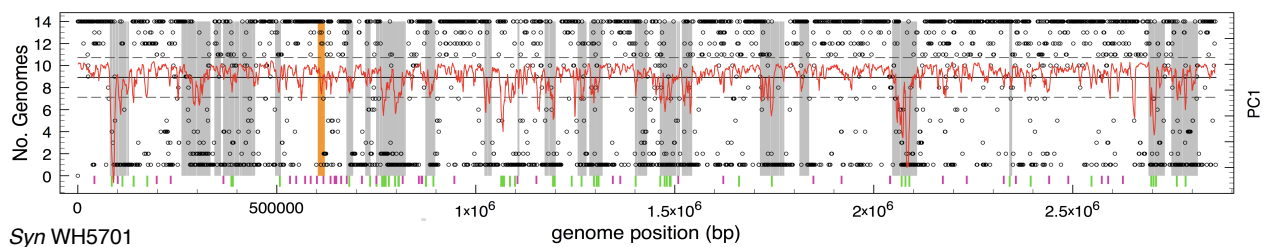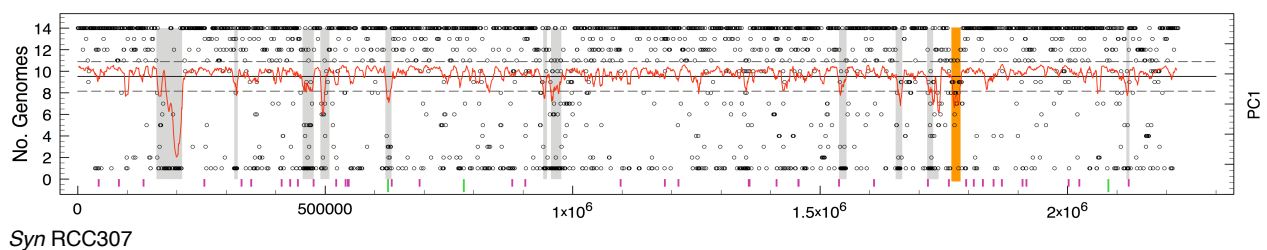

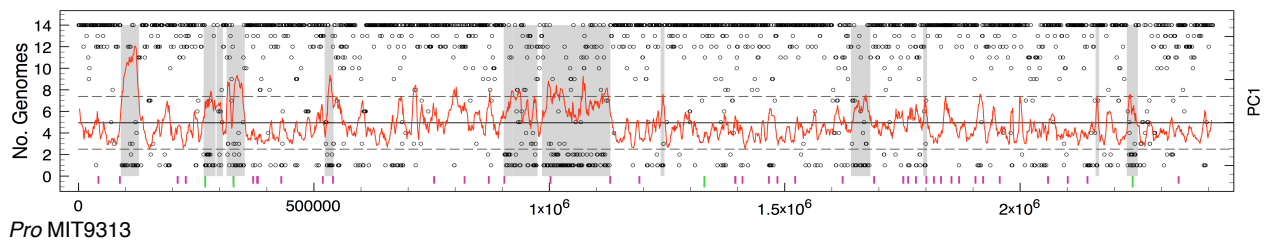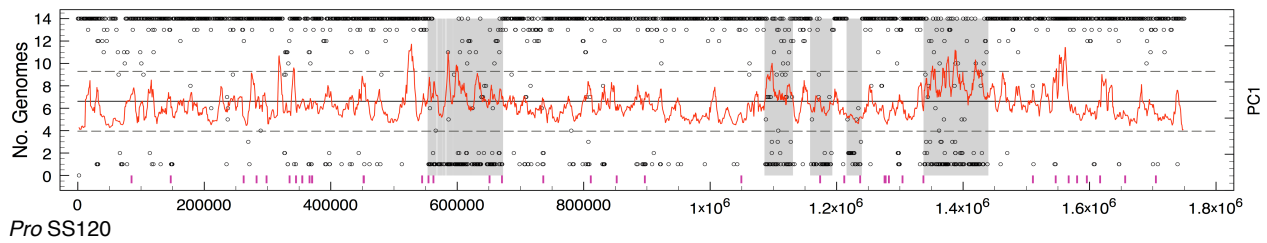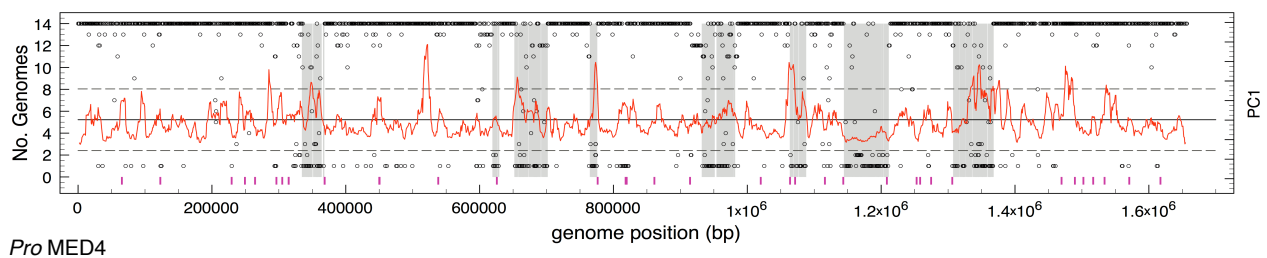

Supplement: Additional data file 3 — Genomes have been re-aligned so that they all start at dnaN. For other details, see the legend of Figure 3. [file gb-2008-9-5-r90-S3.pdf]

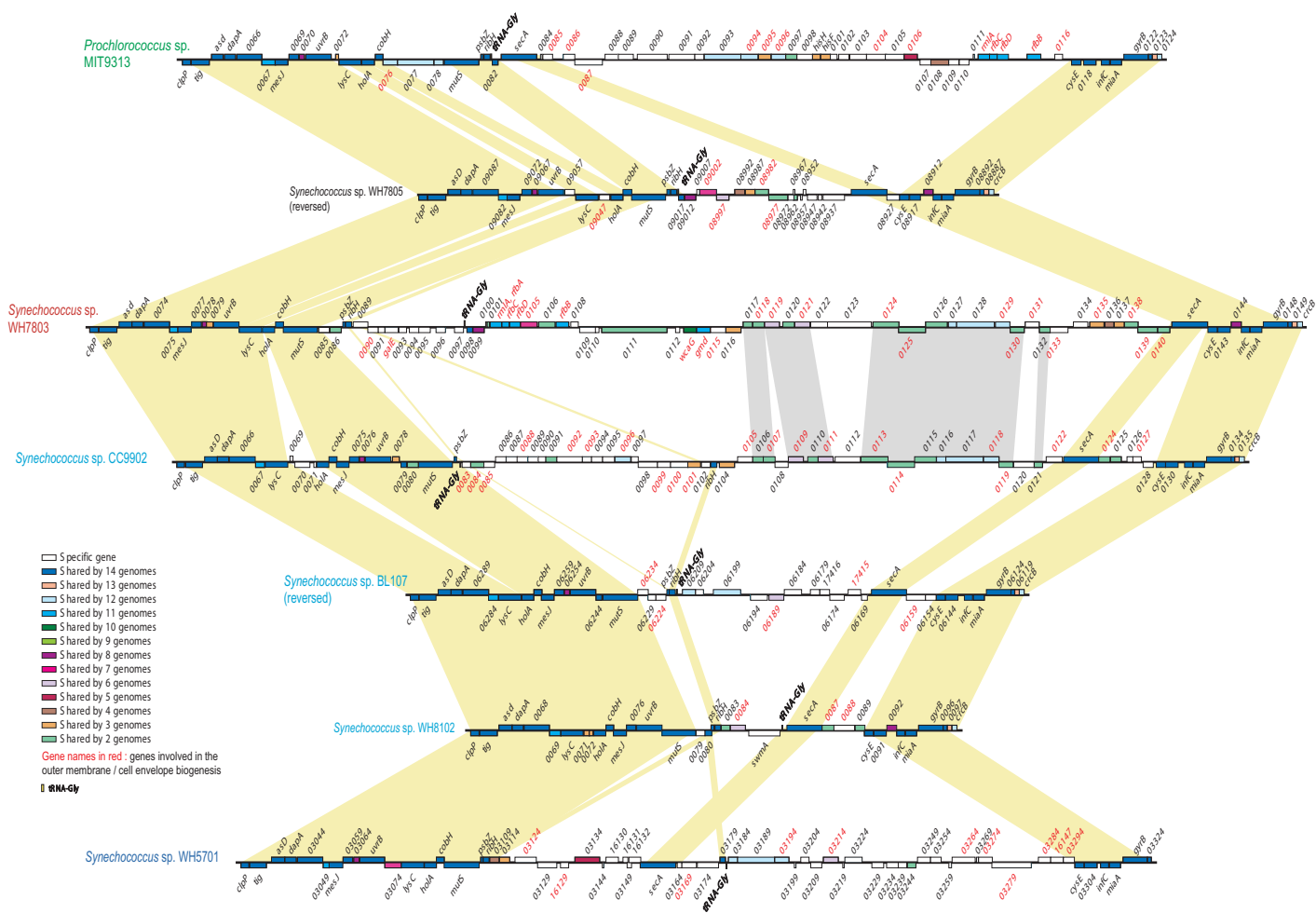

Supplement: Additional data file 4 — Core genome segments surrounding the islands are connected by yellow shading. Genes shared specifically by Synechococcus spp. WH7803 and CC9902 are connected by gray shading. [file gb-2008-9-5-r90-S4.pdf]

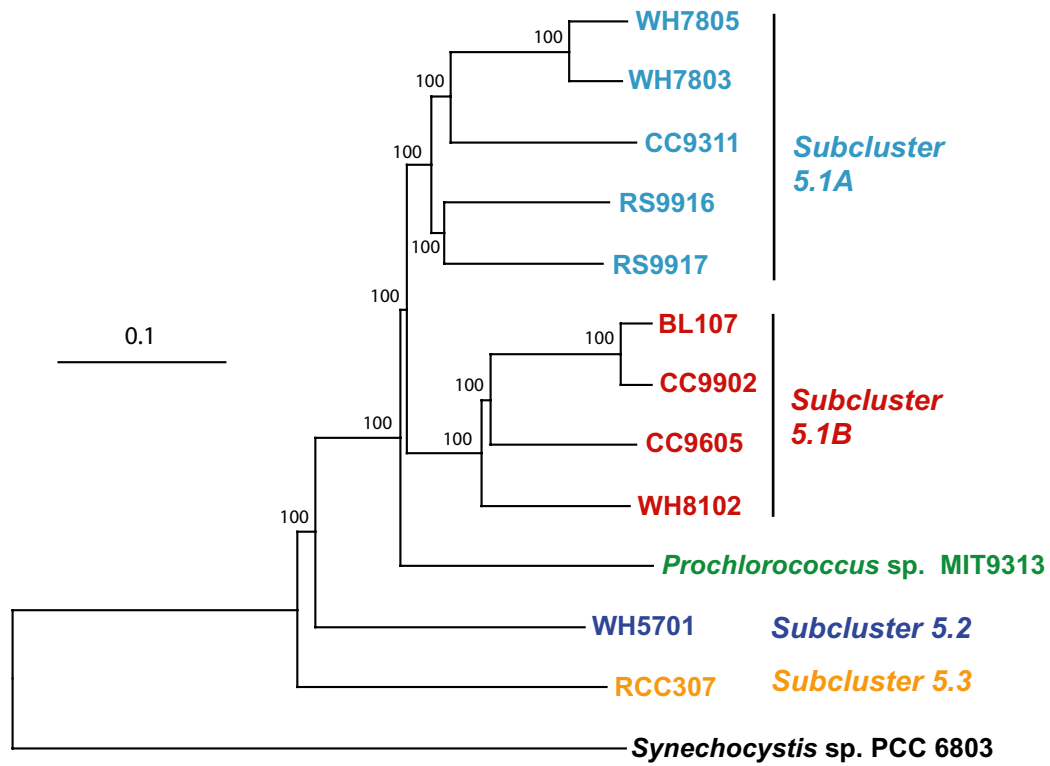

Supplement: Additional data file 6 — NJ tree based on concatenated alignment of the core genome rooted with the freshwater cyanobacterium Synechocystis sp. PCC6803 (863 proteins, 263,424 amino acid positions, gene families with paralogs excluded). [file gb-2008-9-5-r90-S6.pdf]

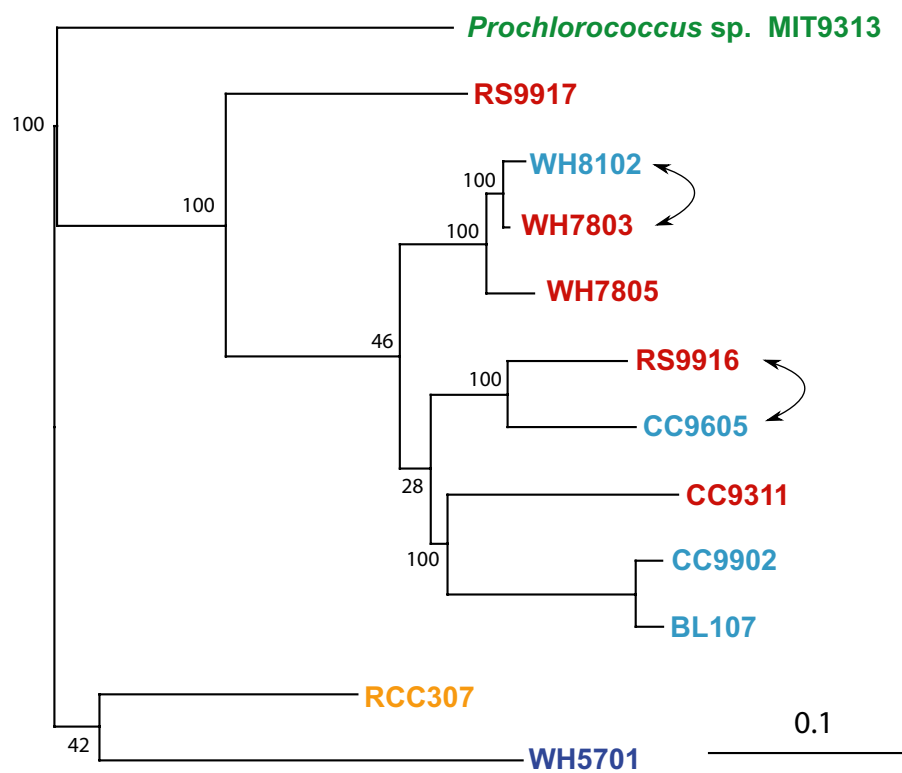

Dufresne et al., additional file 8

Supplement: Additional data file 8 — This enzyme is part of the GS/GOGAT pathway, which is involved in the assimilation of NH4+. This tree suggests at least two transfers between clades III and V (represented by WH7803 and WH8102, respectively) and between clades II and X (represented by RS9916 and CC9605, respectively). [file gb-2008-9-5-r90-S8.pdf]
