# Supplementary material for: Unraveling the genomic mosaic of a ubiquitous genus of marine cyanobacteria
Source: Genome Biol. 2008 May 28;9(5):R90. doi: 10.1186/gb-2008-9-5-r90 (PMC2441476; doi:10.1186/gb-2008-9-5-r90)
Supplement: Additional data file 5 — Island coordinates and island gene composition in the 14 genomes of marine picocyanobacteria used in this study. [file gb-2008-9-5-r90-S5.pdf]

genome	Name	Location (start..stop)	Length (bp)	# genes	Gene descriptions and predicted gene functions<br>*Abbreviations* HK: two component system sensor histidine kinase. RR: two component system response regulator. PTOX: plastoquinol terminal oxidase
**WH8102**	ISL1	342837..483257	140420	146	24 glycosyl transferases or genes involved in cell-envelope biogenesis; 2 phage integrases; metallothionein; urea binding protein; oxidoreductase
	ISL2	620180..633146	12966	9	possible ABC transporter involved in polysaccharide efflux; glycosyl transferase
	ISL3	835928..858919	22991	27	phage integrase; **Plastoquinol terminal oxidase (PTOX);** sodium/glutamate symporter
	ISL4	910261..958945	48684	16	*swmB* (32,375 aa); putative 4-α-glucanotransferase
	ISL5	1088808..1101688	12880	10	possible site-specific recombinase
	ISL6	1121330..1141334	20004	17	2 phage integrases
	ISL7	1151665..1158903	7238	7	
	ISL8	1171474..1182267	10793	12	possible phage integrase family
	ISL9	1334154..1420474	86320	137	Ferredoxin; possible phosphoribulokinase/uridine kinase family protein; phage integrase family; partial homology to phage recombinase; RNA-binding protein; carotenoid binding protein and possible beta-carotene ketolase; ribosomal-protein-alanine acetyltransferase; zeta-carotene desaturase; gamma-glutamyltranspeptidase; cytochrome P450 family protein; agmatine ureohydrolase; hydrogenase expression/formation proteins HypA2 and HypB; nitrate-like ABC transport system; putative Rieske [2Fe-2S] family protein; nitrilase, flavoprotein involved in K+ transport (COG2072); DnaJ domain-containing protein; short-chain dehydrogenase/reductase (SDR) superfamily; hli; 3 transcriptional regulators; glycosyltransferase family 2
	ISL10	1488803..1524352	35549	40	2 phage integrases; pfkB family carbohydrate kinase
	ISL11	1589405..1607641	18236	17	3 phage integrases; conserved hypothetical protein [similar to C-terminal end of type I restriction enzyme HsdR]; glycosyl transferase; galactosyltransferase
	ISL12	1842623..1855485	12862	14	PhoH family protein
	PBS	1900355..1927085	26,730	33	PBS rod gene cluster: pigment type 3b
	ISL13	1980851..1996473	15622	15	phage integrase; transcriptional regulator; ABC transporter, multidrug efflux family
	ISL14	2185139..2207704	22565	14	
	ISL15	2311143..2323308	12165	15	hli5; phage integrase
	ISL16	2377767..2395315	17548	18	ferredoxin--nitrite reductase; cyanate ABC transporter; cyanate lyase, Zn^{2+} ABC transport system
**BL107**	ISL1	185650..199811	14161	9	lipopolysaccharide biosynthesis; two-domain glycosyltransferase
	ISL2	210419..231126	20707	21	nucleoside-diphosphate-sugar pyrophosphorylase, 2-polyprenyl-3-methyl-5-hydroxy-6-metoxy-1,4- benzoquinol methylase; short-chain dehydrogenase family protein; imidazole glycerol phosphate synthase subunits hisH and HisF, oxidoreductase; GFO/Idh/MocA family protein; acylneuraminate cytidyltransferase; pyruvate kinase; SAM-dependent methyltransferase; pyridoxal phosphate-dependent enzyme; 2 Nucleoside-diphosphate-sugar epimerase; pyridoxal phosphate-dependent enzyme; formyl transferase N-terminal domain; cephalosporin hydroxylase
	ISL3	268826..292742	23916	19	flanked by mutS-psbZ-ribH and SecA-cysE,
	ISL4	324754..329733	4979	6	nucleotide sugar epimerase; aminotransferase (degT family); hexapeptide transferase family; N-acetylneuraminic acid synthetase, UDP-N-acetylglucosamine 2-epimerase
	ISL5	548698..560297	11599	13	pilus assembly protein and type-II secretory system components
	ISL6	694562..708415	13853	19	2 CRP transcriptional regulators, ferritin, Fe^{3+} ABC transporter substrate binding protein; 2 porins, flavodoxin, thioredoxin di-sulfide reductase
	PBS	784116..812109	27993	40	PBS rod gene cluster: pigment type 3d
	ISL7	877114..907762	30648	48	phage integrase; hli; band 7 protein; sulfotransferase
	ISL8	1083188..1106833	23645	27	RR*; nuclease; helicase; DnaJ domain protein
	ISL9	1130990..1146375	15385	18	Phycobilin:C-phycoerythrin II lyase MpeZ; AraC family transcriptional regulator
	ISL10	1230865..1251942	21077	29	hli
	ISL11	1418358..1434714	16356	25	DnaJ domain protein; DEAD/DEAH box helicase protein; DNA ligase; exonuclease; hli
	ISL12	1559991..1568591	8600	10	TerC family
	ISL13	1589476..1597507	8031	13	
	ISL14	1672128..1707066	34938	52	2 Ferredoxins (2Fe-2S); **Fe stress-induced chlorophyll-binding protein (IsiA),** Photosystem II PsbY-like; phycobilisome rod-core linker polypeptide, Superoxide dismutase [Cu-Zn]; hli, carotenoid binding protein and beta-carotene ketolase; phosphoribulokinase/uridine kinase family
	ISL15	1781540..1785433	3893	10	
	ISL16	1832825..1849107	16282	22	**PTOX**; phage integrase, sodium/glutamate symporter
	ISL17	2009823..2025378	15555	15	2 x glycosyltransferases; 5 genes involved in carbohydrate efflux and metabolism; DEAD/DEAH box helicase-like protein
	ISL18	2063291..2075721	12430	17	succinate dehydrogenase cytochrome b-556 subunit, succinate dehydrogenase/fumarate reductase - flavoprotein and Fe-S protein subunits
	ISL19	2188340..2217860	29520	38	possible integrase/recombinase; PTOX (possible psuedogene); putative phycobilisome linker polypeptide - C-phycoerythrin class II-associated; DEAD/DEAH box helicase-like protein; band 7 protein; nuclease; sulfotransferase; metallopeptidase
**CC9311**	ISL1	142873..203140	60267	59	putative phosphatase; 13 genes involved in sugar modification; 2 glycosyltransferases
	PBS	472850..507894	35044	44	PBS rod gene cluster: pigment type 3d
	ISL2	543480..559014	15534	20	two component system; DNA binding response regulator; trehalose synthase; glycerol dehydrogenase; glycerol kinase; glycoside hydrolase
	ISL3	578128..586825	8697	7	
	ISL4	602561..650368	47807	57	**2 ferritin** genes and ferrous iron transport system; 3 possible pilins, prepilin-type N-

					terminal cleavage/methylation domain protein; two component system (RR and HK*), HK; porin; periplasmic amino acid-binding protein
	ISL5	659397..669901	10504	15	two component system (RR and HK);type I secretion target GGXGXDXXX repeat protein; possible carbamoyl-phosphate synthase L chain
	ISL6	726433..729446	3013	7	
	ISL7	748273..759035	10762	12	AraC-type regulatory protein; transporter; major facilitator family protein
	ISL8	768612..810983	42371	57	**Ferritin**, bacterial metalothionein; glycine/betain transporter; photosystem I reaction center subunit psaK (photosystem I subunit X); glyceraldehyde-3-phosphate dehydrogenase
	ISL9	859031..868292	9261	17	-
	ISL10	967259..1045531	78272	106	**Ferritin**; sensory box HK/RR, DNA-binding response regulator, two component system; glutathione S-transferase; hli; possible light-dependent protochlorophyllide oxido-reductase
	ISL11	1091094..1105772	14678	8	ABC transporter RzcB; DNA-binding response regulator
	ISL12	1120624..1146598	25974	32	Ferrochelatase; carbamoyl-phosphate synthase L chain, phycobilisome rod-core linker polypeptide (L-RC 28.5); glutamine synthetase; type II alternative RNA polymerase sigma factor, sigma-70 family protein; ribonucleotide reductase (Class II); nuclease
	ISL13	1325058..1408018	82960	78	manganese/zinc/iron chelating ABC transporter (MZT) family; 2 porins; iron-regulated protein A precursor; thioredoxin-like, thiol oxidoreductase; Band 7 protein; DnaJ domain; multicopper oxidase
	ISL14	1451553..1457356	5803	8	glutamine synthase, putative urea transporter
	ISL15	1486354..1490968	4614	11	-
	ISL16	1734060..1739896	5836	12	**Flavodoxin**; thioredoxin reductase; ferredoxin
	ISL17	1832673..1845994	13321	24	2 hli
	ISL18	1921309..1926142	4833	15	hli
	ISL19	1958139..1970184	12045	20	Hli; CpeY protein; transcriptional regulator, araC family
	ISL20	2012780..2019900	7120	8	photosystem II PsbY
	ISL21	2109129..2143307	34178	47	phage integrase; bacteriophage protein homolog; bacterial metallothionein; hli, ZIP family transporter; TonB-dependent receptor protein; ParB nuclease
	ISL22	2338922..2368808	29886	32	iron-containing alcohol dehydrogenase; antibiotic biosynthesis monooxygenase; sensory box/GGDEF family protein; peroxidase; carboxyphosphonoenolpyruvate phosphonomutase; enantiomer-selective amidase; vanadium-dependent bromoperoxidase, mechanosensitive ion channel family protein; nicotinate-nucleotide pyrophosphorylase
	ISL23	2415128..2432141	17013	24	cyclic nucleotide-binding domain protein; putative adenylate cyclase; CRP transcriptional regulator and 2 other transcriptional regulators; RND multidrug efflux transporter
**CC9605**	ISL1	129900..149841	19941	16	-
	PBS	417023..441121	24098	30	PBS rod gene cluster: pigment type 3d
	ISL2	513856..551287	37431	48	Band 7 protein; GAF domain; phage integrase; putative maleylacetoacetate isomerase; carboxymuconolactone decarboxylase family protein
	ISL3	733747..751967	18220	17 19	Nuclease; ATPase involved in chromosome partitioning; DNA-directed RNA polymerase
	ISL4	774489..804804	30315	24	ethyl tert-butyl ether degradation EthD; ankyrin; cholesterol oxidase; proline-specific peptidase
	ISL5	830270..836830	6560	6	-
	ISL6	894575..920454	25879	33	3 phage integrase, LexA repressor
	ISL7	989206..1050790	61584	80	putative rieske (2Fe-2S) family protein; nitrate-like ABC transporter and substrate-binding protein; aliphatic sulphonates hydrogenase accessory protein HypB; putative hydrogenase expression/formation protein HypA2, putative agmatine ureohydrolase; zeta-carotene desaturase; porin, phosphonate-binding periplasmic protein; ACR3 family arsenite transporter; chromate transporter
	ISL8	1093864..1102271	8407	12	Formate hydrogenlyase subunit 3/multisubunit Na+/H+ antiporter MnhD subunitm (2R)-phospho-3-sulfolactate synthase; extracellular solute-binding protein
	ISL9	1150493..1159896	9403	11	-
	ISL10	1287134..1294908	7774	8	site-specific recombinase XerD
	ISL11	1326213..1337754	11541	13	-
	ISL12	1444972..1461680	16708	17	CRP family transcriptional regulator; porin; putative iron ABC transporter, substrate binding protein; ferritin; ferredoxin (2Fe-2S); thioredoxin-disulfide reductase; flavodoxin; HTH of cAMP family transcriptional regulator; chlorophyll a/b binding light harvesting protein PcbD
	ISL13	1532982..1632606	99624	137	3 phage integrases; 4 cation efflux transporters (CDF family); carbamoyl-phosphate synthase L chain; possible ICC (3',5'-cyclic-nucleotide phosphodiesterase); ferric uptake regulator, fur; glutathione S-transferase
	ISL14	1816149..1826961	10812	10	heavy metal translocating P-type ATPase
	ISL15	1954045..1962679	8634	10	RecA-family ATPase, phage integrase family
	ISL16	2021232..2063085	41853	41	9 genes involved in cell envelope biogenesis; site-specific recombinase XerD-like; weak similarity to phage integrase
	ISL17	2096645..2105675	9030	11	-
	ISL18	2284523..2327370	42847	37	transcriptional regulator, Crp/Fnr family; adenylyl cyclase class-3/4/guanylyl cyclase; cyclic nucleotide-binding domain (cNMP-BD) protein; 2 ATPases, prolyl 4-hydroxylase, α subunit; possible diaminopimelate decarboxylase acetyltransferase
	ISL19	2384560..2399989	15429	17	phage integrase; ATPase, phycobilisome rod-core linker polypeptide (L-RC 28.5); flavodoxin
**CC9902**	ISL1	82945..138343	55398	46	13 glycosyltransferases or outer membrane/cell envelope biogenesis; 3 TPR repeat proteins and a cyclic nucleotide-binding domain (cNMP-BD) protein. common with BL107-ISL3 and WH7803-ISL1

	ISL2	444980..482936	37956	41	possible integrase/recombinase; **PTOX**; DEAD/DEAH box helicase, band 7 protein; nuclease; sulfotransferase; N-acylneuraminate-9-phosphate synthase; acylneuraminate cytidylyltransferase; polysaccharide export protein; capsule polysaccharide protein KpsS-like
	ISL3	632884..655372	22488	17	DEAD/DEAH box helicase; fumarate hydratase, ABC transporter involved in polysaccharide efflux; 5 related to LPS or cell envelope biogenesis; sodium/bile acid cotransporter family
	ISL4	947613..983999	36386	43	possible beta-kerotene ketolase; orange carotenoid protein OCP; marine cyanobacterial specific DnaJ, transcriptional regulator LuxR family, Cu/Zn SOD; 2 Ferredoxin (2Fe-2S); hli; phycobilisome rod-core linker polypeptide, iron-stress induced chlorophyll-binding protein (IsiA), phosphoribulokinase/uridine kinase family protein
	ISL5	990952..1005421	14469	22	Hli; DEAD/DEAH box helicase
	ISL6	1135875..1144414	8539	9	TerC family protein; Transcription-repair coupling factor
	ISL7	1164433..1177043	12610	15	-
	ISL8	1318772..1349329	30557	23	ABC transporter system for Mn^{2+}; multicopper oxidase
	ISL9	1392789..1431518	38729	57	photosystem I subunit IV psaE, metalloprotease, phage integrase, HNH nuclease; possible carbamoyl-phosphate synthase L chain
	ISL10	1504484..1518394	13910	14	AraC family transcriptional regulator; GAF domain, PBS lyase HEAT-like repeat
	ISL11	1726654..1733702	7048	15	
	PBS	1798919..1829076	30,157	42	PBS rod gene cluster: pigment type 3d
	ISL12	1905017..1917810	12793	15	2 porins; **iron ABC transporter**, substrate binding protein; **ferritin**, **Flavodoxin**; Thioredoxin-disulphide reductase; CRP family transcriptional regulator; similar to BL107-ISL6
**WH7803**	ISL1	89122..157357	68235	56	22 glycosyltransferases or outer membrane/cell envelope biogenesis; alkaline phosphatase (with phytase-like insertion)
	PBS	487139..511524	24385	34	PBS rod gene cluster: pigment type 3a
	ISL2	555917..566496	10579	9	ATP-type multidrug efflux pump
	ISL3	802561..932888	130328	201	metallo-beta-lactamase superfamily hydrolase; multisubunit Na^{+}/H^{+} antiport system - MnhBGDC subunits; drug/metabolite transporter (DMT) superfamily efflux protein, cytochrome C6; permease, 2 complete ABC-type Mn^{2+}/Zn^{2+} transport systems; porin; hydrogenase accessory membrane protein; redox protein; SAM-dependent methyltransferase; dehydrogenase; chalcone synthase; cytochrome P450
	ISL4	962344..968866	6522	9	drug/metabolite transporter (DMT) superfamily efflux protein; glutathione synthetase fused with a acetyltransferase domain; cysteine dioxygenase, type I
	ISL5	1116645..1130606	13961	14	Flanked by tRNA; SphX; ABC-type sugar transport sytem, *ggtBCDA*; glucosylglycerol-phosphate synthase; peroxiredoxin; chaperone HtpG
	ISL6	1197181..1205296	8115	9	ABC transporter, membrane component; permease of the major facilitator superfamily
	ISL7	1372168..1377305	5137	9	-
	ISL8	1530393..1538427	8034	17	-
	ISL9	1636666..1650587	13921	16	3 HK; 1 RR and 6 pili subunit superfamily proteins
	ISL10	1752848..1761145	8297	16	-
	ISL11	1769991..1779555	9564	10	
	ISL12	1932160..1942822	10662	18	Putative cyanophycin synthetase; TPR-repeat-containing protein
	ISL13	2086183..2096644	10461	10	Permease; possible glyoxalase; putative dioxygenase; phytoene synthase; RR, HK and a hybrid RR/HK
	ISL14	2209175..2226030	16855	13	Arginine decarboxylase; CRP-family transcriptional regulator and adenylate cyclase
**WH7805**	ISL1	54887..69301	14414	18	catalase
	ISL2	115771..248365	132594	195	3 giant orfs (>3,000 aa); oxidoreductase; 4 ribosomal protein S6 paralogs, DNA/RNA helicases; ABC-type MN transport system; Cu-SOD; 2 Band 7 proteins; glycosyl transferases
	ISL3	258650..270876	12226	13	Ferritin; CRP family transcriptional regulator; iron ABC transporter, substrate binding protein; uncharacterized iron-regulated protein
	ISL4	426986..440993	14007	19	ABC transport systems for phosphate and sugars; 2 ferredoxins, alkyl-hydroperoxide reductase; ATP phosphoribosyltransferase involved in histidine biosynthesis; Hsp90; glucosylglycerol-phosphate synthase
	ISL5	470171..480677	10506	11	DNA ligase; ATP-dependent helicase and chromate transporter
	ISL6	519000..532914	13914	9	1 giant ORF
	ISL7	632033..658530	26497	7	giant ORF (14,802 aa); ATPase PulE; type I secreted protein
	ISL8	856020..878324	22304	24	phage integrase; SOS function regulatory protein; LexA repressor; giant orf (10,622 aa)
	ISL9	940855..949292	8437	10	SOD; putative multidrug efflux ABC transporter; peptidylprolyl isomerase
	ISL10	974776..993373	18597	22	Type I restriction-modification system; partial phage integrase, phage integrase, site-specific recombinase; transcriptional regulator
	ISL11	1012046..1023828	11782	14	3 HKa, 1 RR
	ISL12	1029064..1041414	12350	14	Urea transporter; ABC-type branched-chain amino acid transport systems; 2 substrate binding proteins
	PBS	1127386..1147228	19842	29	PBS rod gene cluster: pigment type 2. **Nb. No tetranucleotide deviation**
	ISL13	1220657..1229151	8494	6	glycosyl transferase and exopolysaccharide biosynthesis protein
	ISL14	1343142..1354859	11717	6	2 RR, 1 HK and 1 giant ORF (5,018 aa)
	ISL15	1436806..1492545	55739	47	7 glycosyl transferases and 9 other genes involved in sugar modification or cell wall biogenesis; 6 orfs containing TPR repeats
	ISL16	1526050..1558961	32911	33	Polyferredoxin; Na+/proline symporter; 2 ribokinase family, MutY; dihydroxyacetone kinase; site-specific recombinase; glycosyl transferase - related to UDP-glucuronosyltransferase; Ca2+-binding protein (EF-Hand superfamily); ligand gated channel (GIC family)
	ISL17	1574067..1586886	12819	16	alkaline phosphatase-5'nucleotidase; alkaline phosphatase; putative ABC-type polysaccharide/polyol phosphate export system; 4 glycosyl transferases and

					sugar transferases involved in lipopolysaccharide synthesis
	ISL18	1739247..1746384	7366	9	aspartate carbamoyltransferase, asparagine synthetase protein, N-carbamoyl-L-amino acid amidohydrolase
	ISL19	1866542..1871593	5051	6	flanked by a site-specific recombinase and a phage integrase
	ISL20	1894599..1986828	92229	52	3-phosphoglycerate kinase; Universal stress protein UspA; cytochrome P450, delta-aminolevulinic acid dehydratase, predicted permease, pseudouridylate synthase; giant orf (8,130 aa), ATPase components of various ABC-type transport systems; 2 guanosine polyphosphate pyrophosphohydrolases; PPE-repeat proteins; giant orf (3780 aa), TPR repeat protein; AraC-type transcriptional regulator; GTPase; permeases; nicotinate-nucleotide pyrophosphorylase; arginyl-tRNA synthetase; L-asparaginase; ABC-type amino acid transport; PEP phosphonomutase; 3 RTX toxins and related Ca2+-binding proteins; GMP synthase; adenylate cyclase
	ISL21	2175292..2204492	29200	38	DnaJ; transcriptional regulator containing PAS AAA-type ATPase and DNA-binding domains
	ISL22	2369502..2420721	51219	72	phage integrase; RR; 6 RTX toxins and related Ca^{2+}-binding proteins
**RS9917**	ISL1	1..17807	17806	65	prophage CP4-like integrase
	ISL2	71361..131250	59889	74	MN-SOD; 2 transposases; RR; porin; arsenate efflux pump, arsenical resistance operon repressor; glyceraldehyde-3-phosphate dehydrogenase; multidrug efflux transporter; ABC-type transporter, phosphate binding protein; beta-carotene ketolase and carotenoid binding protein
	ISL3	249223..345033	95810	14	RR, 2 HK; ABC-type transporter, substrate binding protein
	ISL4	375209..387799	12590	18	
	ISL5	491952..504125	12173	13	HK, RR, HK, 2 pilins
	PBS	600115..609267	9152	13	PBS rod gene cluster: pigment type 1. Nb no tetranucleotide deviation
	ISL6	800990..863367	62377	62	2 transposases; integron integrase; phage integrase; putative ATP-dependent Lon protease; 2 restriction enzymes; DNA-damage-inducible protein
	ISL7	901468..991077	89609	72	5 transposases (IS elements); ppGpp-regulated growth inhibitor suppressor ChpR/MazE; MazF transcriptional modulator of MazE/toxin; 22 genes related to polysaccharide modification or cell wall biogenesis
	ISL8	1544990..1554348	9350	11	HK, RR; pilT-like
	ISL9	1836314..1889396	53082	80	2 Band 7 proteins; glutamate-1-semialdehyde 2,1-aminomutase; putative glycolate oxidase subunit glcD; Exodeoxyribonuclease III xth; MFS family transporter; ABC-type Fe3+ transport system, membrane component; putative iron ABC transporter, substrate binding protein; 4α-hydroxytetrahydrobiopterin dehydratase (PCD); putative histone deacetylase/AcuC/AphA family protein; Thermostable carboxypeptidase 1; possible high light inducible protein; GCN5-related N-acetyltransferase; putative protein-tyrosine-phosphatase; Deoxycytidine triphosphate deaminase; short-chain dehydrogenase/reductase (SDR) superfamily; Marine cyanobacteria-specific dnaJ domain containing protein; Prevent-host-death protein; Possible zeta-carotene desaturase; Protochlorophyllide reductase; putative large-conductance mechanosensitive channel MscL; transposase; amino acid racemase; Glycoside hydrolase, starch-binding;
	ISL10	2056070..2147008	90938	136	Catalase/peroxidase; pfkB family carbohydrate kinase; outer membrane efflux protein; hli; endonuclease III; periplasmic protein; carotenoid isomerase; cell death suppressor protein; zinc-containing alcohol dehydrogenase superfamily; putative Glutathione S-transferase; N-terminal domain; possible 3-hydroxyacid dehydrogenase; transcriptional regulator; α-acetolactate decarboxylase; diguanylate cyclase/phosphodiesterase (GGDEF & EALdomains) with PAS/PAC sensor(s); 2 Mn^{2+}/Zn^{2+} ABC transport systems; glutamine synthetase type III; sodium:solute transporter family; aspartate carbamoyltransferase; asparagine synthase; N-carbamoyl-L-amino acid amidohydrolase; permease, NADH dehydrogenase subunit N; Cytochrome c, class IC:Cytochrome c, class I; porin; 3 transposases; RR; 2 type I site-specific deoxyribonuclease; 2 Ferritin; 2 ferrous iron uptake (FeoB) family, Iron ABC transport system substrate binding protein; 2 CRP family transcriptional regulators; peroxiredoxin
	ISL11	2197745..2208898	11153	16	Transposase; acetyltransferase; ATP/GTP-binding protein; 2 ATP-dependent helicases
	ISL12	2431357..2462318	30961	40	HK, RR; sigma-54 factor; coproporphyrinogen III oxidase; *Prochlorococcus*-like magnesium-protoporphyrin IX monomethyl ester aerobic oxidativecyclase; ArsR family regulator; *Synechococcus*-specific Photosystem II protein D1; ABC transporter - membrane component; transcriptional regulator; N-acetylmuramoyl-L-alanine amidase; K+ channel; rieske Fe-S protein; pyridoxamine 5'-phosphate oxidase; possible ICC (3',5'-cyclic-nucleotide phosphodiesterase) protein
	ISL13	2494457..2533537	39080	71	membrane associated glutaredoxin; glutamine amidotransferase class-I; laccase; cyclic nucleotide-binding domain (cNMP-BD); 4 transposases
	ISL1	2564885..2584918	20033		See above
**RS9916**	ISL1	56488..186307	129819	231	phage integrase; glycosyltransferase; fur; possible zeta-carotene desaturase; 2 hli; protochlorophyllide reductase; GAF domain; Cu/Zn SOD; Zn-dependent protease
	ISL2	294974..300687	5713	6	giant orf (3,461 aa) hemolysin-type calcium-binding region:RTX N-terminal domain
	ISL3	368019..455099	87080	85	ABC-type Mn^{2+}/Zn^{2+} transporter system including 2 binding protein components; metallophosphoesterase; methylglyoxal synthase; porin; chromate transporter; ABC glycine betaine/proline transporter system; glycine-sarcosine methyltransferase; sarcosine-dimethylglycine methyltransferase
	ISL4	471786..482829	11043	12	2 Crp/Fnr family transcriptional regulators; ferritin; porin; putative hydroxylase; iron ABC transport substrate binding protein; 3-phosphoshikimate 1-carboxyvinyltransferase; similar to WH7805-ISL3, BL107-ISL6 and CC9605-ISL12, CC9902-ISL11
	ISL5	805902..826101	20199	38	RR, HK; arsenical resistance operon repressor; Glyceraldehyde -3 -phosphate

					dehydrogenase; MFS family multidrug efflux transporter; nuclease
	ISL6	882411..896822	14411	25	PAS domain sensor protein, HK
	ISL7	1022841..1034884	12043	18	2 cysteinyl-tRNA synthetase; sodium-dependent transporter; 1-deoxy-D-xylulose 5-phosphate reductoisomerase; ferredoxin-like protein
	ISL8	1043673..1054406	10733	25	-
	ISL9	1075627..1104626	28999	40	RR, LexA; esterase/lipase; diaminobutyrate- -2 -oxoglutarate aminotransferase
	ISL10	1162246..1177039	14793	19	Pilin polypeptide PilA-like protein; RR, HK; ABC oligopeptide transport, membrane component
	ISL11	1356713..1384101	27388	62	3 SAP domain proteins
	ISL12	1557439..1570245	12806	22	PhoH family protein
	ISL13	1911806..1920229	8423	7	-
	ISL14	2101108..2106136	5028	6	integrase and site-specific recombinase
	ISL15	2256150..2365287	109137	55	20 genes involved in LPS biosynthesis polysaccharide modification and cell wall biogenesis; DNA/RNA helicase
	ISL16	2469526..2483869	14343	15	TPR repeat protein
	PBS	2614355..2643107	28752	43	PBS rod gene cluster: pigment type 3d
**RCC307**	ISL1	160200..210406	50206	47	23 glycosyltransferases or genes involved in cell wall biogenesis; sulfolipid biosynthesis protein
	ISL2	317519..322085	4566	12	Cu/Zn SOD
	ISL3	455591..476315	20724	50	Hli; nuclease
	ISL4	491496..507407	15911	19	RR, PAS domain sensor protein; HK, RR; pili subunit superfamily; type II secretory pathway component PulD; ABC oligopeptide transporter membrane component; polysaccharide-forming beta-glycosyltransferase
	ISL5	622873..633746	10873	13	Phage integrase; DnaJ domain containing protein
	ISL6	941788..946836	5048	14	-
	ISL7	957618..1002319	44701	76	DMT family multidrug efflux pump; RNA-binding protein, RRM domain; cytochrome P450; transcriptional regulator; FAD-dependent dehydrogenase; Inorganic pyrophosphatase; HK; N-carbamoyl-L-amino acid amidohydrolase; asparagine synthase; aspartate carbamoyltransferase; sodium:solute transporter; rubrerythrin; chromate transporter, CHR family; saccharopine dehydrogenase related protein
	ISL8	1336607..1355738	19131	41	pyridoxamine phosphate oxidase; glycoside hydrolase, family 19; SAM-dependent methyltransferase; short-chain dehydrogenase/reductase; proline/betaine transporter, MFS family; permeases of the major facilitator superfamily; ligand gated channel (GIC family); 2 hli
	ISL9	1539608..1552261	12653	29	secreted oxidoreductase
	ISL10	1653992..1665440	11448	24	gluconolactonase
	ISL11	1717506..1732232	14726	31	Hli; aldehyde dehydrogenase family protein
	PBS	1752176..1788207	36031	49	PBS rod gene cluster: pigment type 3c
	ISL12	2098337..2123807	25470	29	HK, 2 RR; phosphate/phosphonate ABC type transporter, substrate binding component; small mechanosensitive ion channel, MscS family, valyl-tRNA synthetase
**WH5701**	ISL1	252137..291720	39583	39	molybdate ABC transporter permease and periplasmic protein components; cyanate ABC transport system; Cro/CI family transcriptional regulator; transcriptional regulator; 2 transposases; phage integrase; metallo-beta-lactamase superfamily hydrolase
	ISL2	309607..374711	65104	84	alkaline phosphatase; molybdopterin binding oxidoreductase; arylsulfatase; glutamate-ammonia ligase; glutathione-s-transferase; NAD-dependent pyridoxamine 5'-phosphate oxidase/oxidoreductase; AraC family transcriptional regulator; MerR family transcriptional regulator; transcriptional regulator; P-type ATPase transporter for Cu; monooxygenase; 2 transposases,
	ISL3	502829..549693	46864	38	4 transposases; 14 genes involved in polysaccharide modification and cell-wall biogenesis
	ISL4	682879..754079	71200	99	possible beta-carotene ketolase; carotenoid binding protein; type-I restriction modification system; ATP dependent DNA helicase; GAF domain; amine oxidase
	ISL5	765571..781207	15636	14	proteobacterial arylsulfatase; AraC family transcriptional regulator; nonspecific acid phosphatase precursor
	ISL6	787512..865874	78362	72	mutS; ferredoxin-nitrite reductase; nitrate reductase; cyanate ABC transport system; 2 glutamine synthases; 2 guanylate cyclases; cyclic nucleotide binding domain protein; other genes related to molybdenum cofactor biosynthesis; 2 transposases
	ISL7	918105..931585	13480	10	transcriptional modulator of MazE/toxin; putative transcriptional regulator; integron integrase and transposase
	PBS	1026414..1039833	13419	20	PBS rod gene cluster: pigment type 1. Nb no tetranucleotide deviation
	ISL8	1097389..1112375	14986	25	Transposase; relE
	ISL9	1144622..1156924	12302	18	HK, RR; transposase
	ISL10	1171890..1245139	73249	81	prophage CP4-like integrase; 9 transposases; 2 hli; glutatione-s-transferase, C terminal domain; 2 helicases; zeta-carotene desaturase; catalase/peroxidase
	ISL11	1295763..1319594	23831	23	V-type ATP synthase subunits A-K; acetate kinase; D-lactate dehydrogenase; isoamylase; phosphoketolase, 3 transposases
	ISL12	1444188..1460860	16672	27	DEAD/DEAH box helicase
	ISL13	1482017..1531405	49388	7	SphX -periplasmic phosphate binding protein; Na/Pi cotransporter
	ISL14	1595357..1622223	26866	32	glycoside hydrolase; ATP/GTP binding protein; pilT; 2 transposases
	ISL15	1678319..1698802	20483	25	2 spermidine synthases; 2 DNA ligases; 2 serine proteases; transposase
	ISL16	1707479..1739362	31883	38	3 transposases; lipase; Mo-dependent nitrogenase; band 7 protein; Iron/Ascorbate oxidoreductase; Xre family transcriptional regulator
	ISL17	1822366..1851398	29032	36	type I restriction-modification system; glyceraldehyde 3-phosphate dehydrogenase; α,α-trehalose-phosphate synthase; nitroreductase
	ISL18	1884942..1934476	49534	54	5 transposases; 2 periplasmic phosphate-binding protein, sphX; transcriptional regulator; cytochrome oxidase; magnesium chelatase subunits ChlD and H;

					exonucleases
	ISL19	1940178..1964690	24512	34	2 hli; ABC transport system, P-type ATPase transporter for copper
	ISL20	2136401..2195774	59373	67	13 glycosyltransferases or genes involved in polysaccharide modification; transposase; ClpB
	ISL21	2235633..2258518	22885	23	6 glucosyltransferases; 3 hybrid HK-RR; RR; PAS domain sensor HK; Sigma-B negative regulator; anti-sigma factor antagonist
	ISL22	2468648..2568840	100192	83	12 glycosyltransferases; ABC multidrug efflux transporter; plasmid stabilisation protein; pilT; 3 transposases
	ISL23	2762812..2768610	5798	9	Transposase; cyclic nucleotide-binding domain (cNMP-BD) protein
**MED4** * from coleman et al., 2006	ISL1*	334900..368300	33400	36	flanked by 2 tRNA; cyanate transporter andlyase; restriction-modification genes; Range:PMM0351-0386.
	ISL1.1	620091..628646	8555	11	possible 5'-3' exonuclease; Glyoxalase/Bleomycin resistance protein; NAD-dependent DNA ligase N-terminus
	ISL2*	652800.. 701269	48469	54	PhoBR; porin; PstS; PtrA; ABC transport system for phosphate (PSTCAB); chromate transporter, CHR family; possible Zinc finger, C2H2 type; Elongation factor Tu domain 2; 2 hli; DDT domain; Hepatitis C virus envelope glycoprotein; Short-chain dehydrogenase/reductase (SDR) superfamily; Glutamine amidotransferase class-I; D12 class N6 adenine-specific DNA met; DnaJ central domain (4 repeats);; LysM domain; DUP family;; potassium channel, VIC family; Lipoprotein; multidrug efflux transporter, MFS family; glyceraldehyde 3-phosphate dehydrogenase; ArsR; chorismate binding enzyme; arsenite transporter, ACR3 family; Poly A polymerase regulatory subunit; Myosin N-terminal SH3-like domain; ATP binding subunit; Serine/threonine specific protein phosphatase; DEAD/DEAH box helicase:Helicase C-terminal domain; DNA ligase;COMC family; Major surface glycoprotein; Helix-turn-helix protein, copG family; α-2-macroglobulin family N-terminal
	ISL2.1	765679..775021	9342	13	Rieske iron-sulfur protein 2Fe-2S subunit; 4 hli, S1 RNA binding domain; Cytochrome oxidase c subunit VIb
	ISL2.2	932990.. 981120	48130	60	2 putative multidrug efflux ABC transporters; HNH endonuclease; ATP synthase protein; purine phosphoribosyltransferase related protein; 30S Ribosomal protein S21; Helix-hairpin-helix DNA-binding motif class 1; ATP/GTP-binding site motif A (P-loop); GCN5-related N-acetyltransferase; Glutathione peroxidase; putative nitrogen regulation protein NifR3 family homolog; 4-hydroxybenzoyl-CoA thioesterase family active site;possible lactate/malate dehydrogenase, α/β; ABC transport system possibly for Mn or Zn, Ferric uptake regulator family, possible Zur; Cobalamin synthesis protein/P47K; DNA gyrase/topoisomerase IV, subunit
	ISL3*	1071500..1082200	10700	9	flanked by tRNA-Pro2; 1 hli inside, 1 hli inflanking 5' region, 1 hli in flanking 3'region;repeats; 6 proteins with predictedtransmembrane domains. Range: PMM1123-1131.
	ISL4*	1141700..1216200	74500	66	Flanked by tRNA-Arg at 5' end, also containstRNA-Ala near 3' end; LPS genes; Range:PMM1196-1261
	ISL5*	1323000..1367600	44600	53	No tRNA; 8 hli; Range: PMM1375-1427
**SS120**	ISL1	554663..671231	116568	152	ABC-type phosphate transport system, *pstCAB*; DNA/RNA helicase; retron-type reverse transcriptase; AbrB family trancriptional regulator fused to LRR containing domain; DNA helicase, predicted restriction/modification system component, ortholog of BS_yeeB; Type II restriction enzyme, methylase subunit; NADPH-dependent reductase; ABC-type polar amino acid transport system ; nucleotide-diphosphate-sugar epimerase; UDP-N-acetylmuramyl pentapeptide phosphotransferase; Nucleoside-diphosphate-sugar epimerase; 2 Glycosyltransferase; succinate dehydrogenase; ATPase; DNA repair exonuclease; Restriction endonuclease S subunit; Type I restriction-modification system methyltransferase subunit; Short-chain dehydrogenase/reductase family; glycosidase; Predicted hydrolase, HAD superfamily; Phosphoribulokinase/uridine kinase family enzyme; undecaprenyl pyrophosphate phosphatase; Porin
	ISL2	1087360..1129760	42400	69	Secreted pentapeptide repeats protein; flavodoxin; chlorophyll a/b-binding light-harvesting protein PcbD, PcbB, PcbH; hli8; hli7; heme iron utilization protein; 5'-phosphoribosylglycinamide transformylase; oxidoreductase; ATPase components of ABC transporter; RNA-binding protein, RRM domain; dioxygenase; permease; Mn2+ and Fe2+ transporter, NRAMP family; MIF/Phenylpyruvate tautomerase family protein; 2 Fatty acid desaturase; Nucleoside 2-deoxyribosyltransferase; phosphoribosyltransferase; Membrane serine protease of rhomboid family; Glutathione peroxidase
	ISL3	1338872..1438725	99853	141	adenine phosphoribosyltransferase; SAM-dependent methyltransferase; Chlorophyll a/b binding light harvesting protein PcbE; GMP synthase - Glutamine amidotransferase domain; Na+-dependent transporter of the SNF family; DegT/DnrJ/EryC1/StrS aminotransferase family enzyme; Metallo-beta-lactamase superfamily hydrolase; SAM-dependent methyltransferase; Permease of the drug/metabolite transporter, DMT superfamily; Pyrimidine dimer DNA glycosylase/Endonuclease V; cytochrome b559 subunit beta; ABC-type Mn^{2+}/Zn^{2+} transport system; 2 GTPase, G3E family; hli13; hli10; hli12; hli11; hli1; hli3; Gamma-glutamyltransferase; Kef-type K^+ transport system predicted NAD-binding component; Light dependent protochlorophyllide oxido-reductase; Na^+/alanine symporter; UDP-N-acetylglucosamine enolpyruvyl transferase; RR; HK; Zn-dependent protease; Predicted redox protein; ATP-dependent Clp protease adaptor protein ClpS; Predicted calcineurin family phosphoesterase; Beta-lactamase class C and other penicillin binding proteins; PtrA; 2 PstS; Permease of the major facilitator superfamily; Glyceraldehyde-3-phosphate dehydrogenase; Transcriptional regulator; GTP cyclohydrolase I
	ISL4	1159507..1192997	33490	31	Zn-dependent hydrolase of the beta-lactamase fold, partial; calcineurin family phosphoesterase; Glycyl-tRNA synthetase, α subunit; Porin homolog; hydroxylase;

					ABC-type Fe3+ transport system periplasmic component; DNA helicase; SAM-dependent methyltransferase; hydrolase, HAD superfamily; Transketolase; 1-deoxy-xylulose 5-phosphate synthase; NAD dependent epimerase/dehydratase; 2OG-Fe(II) dioxygenase superfamily protein; SAM-dependent methyltransferase; UDP-galactopyranose mutase; Chlorophyll a/b binding light harvesting protein PcbF; Adhesin-like protein
	ISL5	1217122..1240286	23164	20	polysaccharide export-related periplasmic protein; Phosphatase, HAD superfamily; FAD dependent oxidoreductase; Nucleotidyl transferase family enzyme; Phosphoheptose isomerase; ADP-heptose synthase; NAD dependent epimerase/dehydratase; SAM-dependent methyltransferase; 4 Glycosyltransferase; ABC-type multidrug transport system ATPase and permease components; SAM-dependent methyltransferase; beta-1,4-N-acetylglucosaminyltransferase
**MIT9313**	ISL1	91775..126554	34779	28	15 glycosyl transferases or LPS/cell-envelope biogenesis related genes
	ISL2	267637..306506	38869	33	putative L-cysteine/cystine lyase; phage integrase; Ser/Thr protein phosphatase; DNA polymerase III beta subunit; Indole-3-glycerol phosphate synthase; profilin; lysyl hydrolase; fatty acid desaturase, type 2; NifU-like protein; malate:quinone oxidoreductase; Hemolysin-type calcium-binding region:RTX N-terminal domain; GTP-binding protein LepA; 2 pilins, HK
	ISL3	316426..351256	34830	29	SAM (and some other nucleotide) binding motif:TPR repeat; resolvase, N terminal domain; glycyl-tRNA synthetase, α subunit; porin; peptidase family M20/M25/M40; hydroxylase; iron ABC transporter, substrate binding protein; ComEC/Rec2-related protein; phage integrase; zinc finger, C3HC4 type (RING finger); site-specific recombinase; 3 TPR repeat pronteins; Staphylococcus nuclease (SNase) homologue; cation-dependent mannose-6-phosphate; TPR repeat; Kelch motif
	ISL4	524676..539884	15208	17	NADH-Ubiquinone/plastoquinone; glutamate decarboxylase; GCN5-related N-acetyltransferase; short-chain dehydrogenase/reductase (SDR) superfamily; TPR repeat; malic enzyme; DNA polymerase III beta subunit; integrase Zinc binding domain
	ISL5	905025..973231	68206	67	C-type lectin domain; DNA-3-methyladenine glycosylase; SAP domain; pyrimidine dimer DNA glycosylase; HK; DNA gyrase B; photosystem I reaction centre subunit VI; NADH Dehydrogenase (complex I) subunit; serine/threonine specific protein phosphatase:Purple acid phosphatase; HAMP domain; mechanosensitive ion channel, MscS family; isochorismatase hydrolase family; large-conductance mechanosensitive channel mscL; Ferric uptake regulator family; putative glucosylglycerolphosphate phosphatase; RecF protein:ABC transporter; product regulatory protein, luxR family; short-chain dehydrogenase/reductase (SDR) superfamily; CDP-alcohol phosphatidyltransferase; transglycosylase SLT domain; O-Acetyl homoserine sulfhydrylase; homoserine O-succinyltransferase; alkylated DNA repair protein; 'chromo' (CHRromatin Organization MOd; Staphylococcus nuclease (SNase); Guanylate-binding protein, C-terminal; SAP domain
	ISL6	986272..1128137	141865	143	Band 7 protein; DAACS family symporter for glutamate/aspartate/dicarboxylates; pfkB family carbohydrate kinase; Bacterial outer membrane protein; SAP domain; Carbamoyl-phosphate synthase L chain; Hemolysin-type calcium-binding region:RTX N-terminal domain; TPR repeat; thermophilic metalloprotease (M29); similar to zeta-carotene desaturase; flagella basal body rod protein; Kinesin motor domain; Bacterial cytochrome ubiquinol oxidase; DNA polymerase (viral) N-terminal; 2 ABC transporter, multidrug efflux family; Outer membrane efflux protein; pectate lyase; kinesin motor domain; 2 hli; tRNA synthetase class I (E and Q); ABC transporter, substrate binding protein, phosphate; RR phosphate; porin; multidrug efflux transporter, MFS family; glyceraldehyde 3-phosphate dehydrogenase; ArsR family; Alkyl hydroperoxide reductase/Thiol specific antioxidant/ Mal allergens family; putative similar to phytochelatin synthase; NUDIX hydrolase; signal peptidase I; aspartate aminotransferase; Glucose-6-phosphate dehydrogenase, C-ter; Dienelactone hydrolase; Ets-domain; lactate/malate dehydrogenase, α/β; putative proline/betaine transporter, MFS family; glyceraldehyde 3-phosphate dehydrogenase; light-harvesting complex protein;
	ISL7	1238310..1242806	4496	6	phosphofructokinase, 3 hli
	ISL8	1642692..1679953	37261	30	thymidylate synthase; thioredoxin reductase; Helix-turn-helix protein, copG family; sodium:solute symporter, ESS family; Pentapeptide repeats; Serine/threonine specific protein phosphatase; Transthyretin precursor (formerly preal; oxidoreductase; NAD binding site; Carbamoyl-phosphate synthase small chain; phosphate-binding protein; Cytochrome P450 enzyme; Eukaryotic-type carbonic anhydrase; PA-phosphatase related phosphoesterase; ABC-transport system; RNA methylase
	ISL9	1796035..1800723	4688	8	TPR repeat; site-specific recombinase; NADH-Ubiquinone/plastoquinone
	ISL10	2162129..2165967	3838	7	-
	ISL11	2228519..2250153	21634	27	Alanine racemase; FYVE zinc finger; Kelch motif; tRNA synthetase class I; Aldo/keto reductase family; [2Fe-2S] binding domain; Phage integrase; Formyl transferase; bifunctional ornithine acetyltransferase/N-acetylglutamate synthase protein; dephospho-CoA kinase
