# Supplementary material for: Unraveling the genomic mosaic of a ubiquitous genus of marine cyanobacteria
Source: Genome Biol. 2008 May 28;9(5):R90. doi: 10.1186/gb-2008-9-5-r90 (PMC2441476; doi:10.1186/gb-2008-9-5-r90)
Supplement: Additional data file 7 — The 122 core protein families showing a phylogeny divergent from the consensus core protein distance tree shown in Figure 4a (that is, for which at least one event of LGT has occurred), with bipartition supported by bootstrap values ≥ 99%. [file gb-2008-9-5-r90-S7.pdf]

Core protein families showing a phylogeny divergent from the consensus core protein distance tree

Cluster N° in Cyanorak	Gene name	Product
198	miaB	2-methylthioadenine synthetase
858		4-hydroxy-3-methylbut-2-en-1yl diphosphate synthase
1142		ABC transporter, membrane component, involved in Fe-S cluster assembly SufD
1137		ABC-type uncharacterized transport system membrane and ATPase component
1051	bioA	Adenosylmethionine-8-amino-7-oxononanoate aminotransferase
414	alaS	Alanyl-tRNA synthetase
415	speA	Arginine decarboxylase
1122	atpI	ATP synthase A chain
1119	atpF	ATP synthase B chain
1118	atpD	ATP synthase delta chain
663	cobO	ATP:corrinoid adenosyltransferase
366	recN	ATPase involved in DNA repair RecN
1196	crtR	Beta-carotene hydroxylase
385		Branched-chain amino acid aminotransferase
684	csoSCA	Carboxysomal carbonic anhydrase
683	csoS2	Carboxysome shell protein CsoS2
740	crtH	Carotenoid isomerase
459		CinA ortholog, predicted molybdopterin binding domain
8103	cobN	Aerobic cobaltochelatase CobN subunit
688	cobQ	Cobyric acid synthase
352		Conserved hypothetical protein
399		Conserved hypothetical protein
403		Conserved hypothetical protein
1439		Conserved hypothetical protein
1444		Conserved hypothetical protein
1474		Conserved hypothetical protein
1184		Conserved hypothetical protein
1232		Conserved hypothetical protein
1346		Conserved hypothetical protein
1554		Conserved hypothetical protein
1580		Conserved hypothetical protein
2542		Conserved hypothetical protein
833		Conserved protein/domain typically associated with flavoprotein oxygenases, DIM6/NTAB family
285	rnc	Ribonuclease III
495	fusA	Elongation factor EF-G
422	eno	Enolase
261	fabI	Enoyl-[acyl-carrier-protein] reductase (NADH)
367	uvrA	Excinuclease ATPase subunit (Excinuclease ABC subunit A)
749	recB	Exodeoxyribonuclease V beta chain RecB
748	recC	Exodeoxyribonuclease V gamma chain RecC
845		Exoribonuclease R/ribonuclease II
1120	atpG	F0F1-ATP synthase b' subunit
43		Fatty-acid desaturase
134	gltS	Ferredoxin-dependent glutamate synthase
1143	sufC	FeS assembly ATPase SufC
711	folC	bifunctional protein (Folylpolyglutamate synthase / Dihydrofolate synthase)
878		Fructose-1,6-biphosphatase / Sedoheptulose1,7-biphosphate phosphatase
976		Fructose-1,6-bisphosphate aldolase class II
885	chlP	Geranylgeranyl hydrogenase
288	glmS	Glucosamine-6-phosphate synthase
361	purF	Glutamine phosphoribosyl pyrophosphate amidotransferase
203	gst	Glutathione S-transferase
753		Glutathione S-transferase domain fused with a domain of unknown function DUF952
140		Glutathione S-transferase, C-terminal domain
17	gap2	Glyceraldehyde-3-phosphate dehydrogenase
410	gcvP	Glycine cleavage system protein P (pyridoxal-binding)
417		Glycine/D-amino acid oxidase family enzyme
886	glyS	Glycyl-tRNA synthetase, beta subunit
694	lepA	GTP-binding protein LepA
279	ho1	Heme oxygenase
432		Histidinol-phosphate/aromatic aminotransferase related enzyme

Core protein families showing a phylogeny divergent from the consensus core protein distance tree

Cluster N° in Cyanorak	Gene name	Product
499		Hydrogenase/urease accessory protein
396		Conserved hypothetical protein
236	ppnK	Inorganic polyphosphate/ATP-NAD kinase
642	ppa	Inorganic pyrophosphatase
722		Integral membrane protein
702		Integral membrane protein (PIN domain superfamily)
475	ileS	Isoleucyl-tRNA synthetase
678	chlN	Light-independent protochlorophyllide reductase subunit N
259		Lignostilbene-alpha, beta-dioxygenase
1079	lpxB	lipid-A-disaccharide synthase
484		Metal-dependent membrane protease, CAAX family
545	moeB	Molybdopterin synthase sulfurylase
124		multidrug efflux transporter, MFS family
1510		multidrug efflux transporter, MFS family
924		NAD dependent epimerase/dehydratase
406		NAD/FAD-utilizing enzyme apparently involved in cell division
1073	pyrF	Orotidine-5'-phosphate decarboxylase
822	ppiB	Peptidyl-prolyl cis-trans isomerase
812	ccp	Peroxiredoxin
362	purL	Phosphoribosylformylglycinamidine (FGAM) synthase, synthetase domain purL
1992	psaI	Photosystem I reaction center subunit VIII (PsaI)
501	psaL	Photosystem I reaction centre subunit XI (PsaL)
480	psbO	Photosystem II manganese-stabilizing protein (PsbO)
907	psb28	Photosystem II reaction centre Psb28 protein
793		Possible ABC transporter, ATPase component
435		Predicted GTPase
759		Predicted hydrolase or acyltransferase (alpha/beta hydrolase superfamily)
1440		Predicted permease
1354		Predicted serine peptidase (prolyl oligopeptidase family) domain
910	secD	Preprotein translocase SecD subunit
821		Purine nucleoside phosphorylase
8042		Possible ABC multidrug efflux transporter
1014		Possible ABC oligopeptide transporter, substrate binding component
881		Possible ABC transporter, membrane component
1280		Possible CaCA family, sodium/calcium exchanger
949		Possible Glutathione S-transferase, N-terminal domain
804	pykF	Pyruvate kinase
681	rbcL	Ribulose bisphosphate carboxylase, large subunit
303	ahcY	S-adenosylhomocysteine hydrolase
355	rsbU	Serine phosphatase RsbU, regulator of sigma subunit
376	aroE	Shikimate dehydrogenase
202	aroK	Shikimate kinase
589		Sugar kinase, ribokinase family
649		Sugar kinase, ribokinase family
887	sir	Sulfite reductase (Ferredoxin)
928	thrS	Threonyl-tRNA synthetase
660	tal	Transaldolase
291	tktA	Transketolase
890	tsf	Translation elongation factor Ts
1192		Translation initiation factor SUI1
1074	tyrS	Tyrosyl-tRNA synthetase
559		Conserved hypothetical membrane protein
1072		Conserved hypothetical membrane protein
1216		Conserved hypothetical membrane protein
1872		Conserved hypothetical membrane protein
1955		Conserved hypothetical membrane protein
281		Conserved hypothetical protein
739		Conserved hypothetical protein, YdiU family
1420		Uncharacterized FAD-dependent dehydrogenase
384	cobA	Uroporphyrin-III c-methyltransferase
401	valS	Valyl-tRNA synthetase

Green background: genes linked to photosynthesis
